# Safe Synthesis of 4,7-Dibromo[1,2,5]thiadiazolo[3,4-*d*]pyridazine and Its S_N_Ar Reactions

**DOI:** 10.3390/molecules23102576

**Published:** 2018-10-09

**Authors:** Timofey N. Chmovzh, Ekaterina A. Knyazeva, Konstantin A. Lyssenko, Vadim V. Popov, Oleg A. Rakitin

**Affiliations:** 1 N. D. Zelinsky Institute of Organic Chemistry, Russian Academy of Sciences, 119991 Moscow, Russian; tim1661@yandex.ru (T.N.C.); katerina_knyazev@ioc.ac.ru (E.A.K.); 2 Nanotechnology Education and Research Center, South Ural State University, 454080 Chelyabinsk, Russia; popov.ioc@gmail.com; 3 A. N. Nesmeyanov Institute of Organoelement Compounds, Russian Academy of Sciences, 119991 Moscow, Russia; kostya@ineos.ac.ru

**Keywords:** sulfur-nitrogen heterocycles, [1,2,5]thiadiazolo[3,4-*d*]pyridazine, aromatic nucleophilic substitution, X-ray analysis

## Abstract

A safe and efficient synthesis of 4,7-dibromo[1,2,5]thiadiazolo[3,4-*d*]pyridazine from the commercial diaminomaleonitrile is reported. Conditions for selective aromatic nucleophilic substitution of one or two bromine atoms by oxygen and nitrogen nucleophiles are found, whereas thiols formed the bis-derivatives only. Buchwald-Hartwig or Ullmann techniques are successful for incorporation of a weak nitrogen base, such as carbazole, into the [1,2,5]thiadiazolo[3,4-*d*]pyridazine core. The formation of rather stable S…η^2^-(N=N) bound chains in 4,7-bis(alkylthio)-[1,2,5]thiadiazolo[3,4-*d*]pyridines makes these compounds promising for the design of liquid crystals.

## 1. Introduction

Electron-deficient π-conjugated building blocks have been widely applied for the preparation of functional organic dyes and electronic materials [1,2,3]. Heterocycles with high electron affinity have been recently showed to be some of the best strong and synthetically variable acceptors. The role of acceptors is to tune the energy levels of the frontiers orbitals (the highest occupied molecular orbital (E_HOMO_) and the lowest unoccupied molecular orbital (E_LUMO_)) as well as the difference between these energies and, as a consequence, the absorption of the materials based on these molecules [4]. The choice of the acceptor is important for high performance of the materials. Electron-acceptor building blocks (A) should be linked with an at least one electronodonor (D) either directly or through a π-conjugated bridge (π) in small molecules or polymers. Many applications of these materials can be mentioned: dye-sensitized solar cells (DSSCs) [2,5,6], bulk heterojunction solar cells (BHJ) [7,8], n-type organic field-effect transistors [9,10], near infrared absorption and emissions materials [11,12], electrochromic materials [13,14], and many others.

Several heterocyclic acceptors were investigated: benzothiadiazole [15], benzotriazole [16], quinoxaline [17], phthalimide [18], diketopyrrolopyrrole [19], thienopyrazine [20], 1,3,5-triazine [21]. However, there is still a strong demand to new building blocks with high electron deficiency in order to increase light absorption in the near infrared region and ultimately to replace the currently employed and expensive fullerene derivatives.

**Scheme 1:**
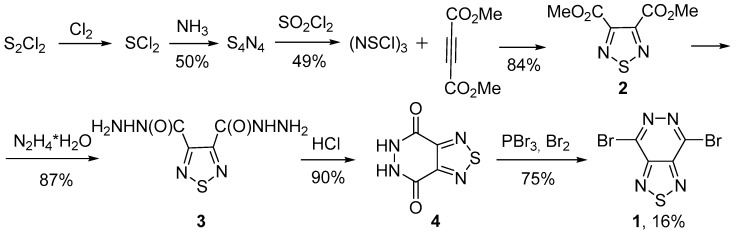
Described synthesis of 4,7-dibromo[1,2,5]thiadiazolo[3,4-*d*]pyridazine (**1**).

Recently we have reported the synthesis of 4,7-dibromo[1,2,5]thiadiazolo[3,4-*d*]pyridazine (**1**) as an electron acceptor with ultrahigh electron deficiency [22] and showed that it can be an important intermediate for the synthesis of the dyes with various possible applications. However, the seven-step synthesis has a few important deficiencies such as the use of shock sensitive explosive tetrasulfur tetranitride (S_4_N_4_) and highly toxic chlorine gas, and a low yield of the target compound (16%) from commercial disulfur dichloride (Scheme 1) which negates the possibility of large-scale synthesis. A key intermediate-dimethyl 1,2,5-thiadiazole-3,4-dicarboxylate (**2**) has to be made under harsh conditions, that can readily be seen as not suitable for commercially viable production of [1,2,5]thiadiazolo[3,4-*d*]pyridazine-based materials. Thus a convenient synthesis of dibromo derivative **1** has great potential for assisting the preparation of a range of photovoltaic materials.

Herein, we describe the safe and efficient synthesis of 4,7-dibromo[1,2,5]thiadiazolo[3,4-d]pyridazine (**1**) and its S_N_Ar reactions as a basis for the preparation of compounds which are of interest as potential photovoltaic materials.

## 2. Results and Discussion

**Scheme 2:**
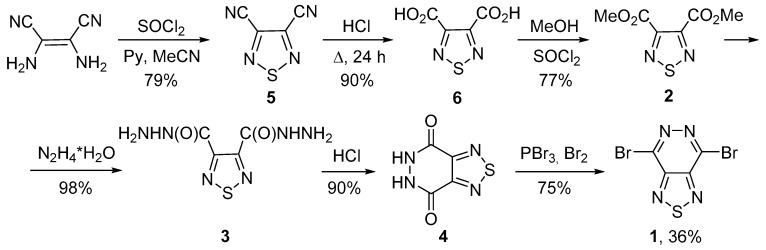
Safe and efficient synthesis of 4,7-dibromo[1,2,5]thiadiazolo[3,4-*d*]pyridazine (**1**).

We have now proposed a shorter (three-step), safe and efficient synthesis of dimethyl 1,2,5-thiadiazole-3,4-dicarboxylate (**2**) from commercially available 2,3-diaminomaleodinitrile (Scheme 2). 1,2,5-Thiadiazole-3,4-dicarbonitrile (**5**) was obtained by treatment of this compound with thionyl chloride [23]. Unfortunately the hydrolysis of dinitrile **5** in basic conditions [23] was unsuccessful, the acid **6** was isolated in only 6% yield. We have found that heating compound **3** under reflux in 6N HCl gave diacid **6** in high yield (90%). Etherification of the diacid **6** with MeOH in the presence of thionyl chloride led to the target diester **2**. Further treatment of the diester **4** with hydrazine hydrate followed by pyridazine cyclisation with hydrochloric acid afforded 5,6-dihydro[1,2,5]thiadiazolo[3,4-d]pyridazine-4,7-dione (**4**) [24]. 

Finally, 4,7-dibromo[1,2,5]thiadiazolo[3,4-*d*]pyridazine (**1**) was prepared in 75% yield by heating 5,6-dihydro[1,2,5]thiadiazolo[3,4-*d*]pyridazine-4,7-dione (**4**) [22] with phosphorus pentabromide (PBr_5_) at 105 °C for 9 h [22] (Scheme 2).

The overall yield of 4,7-dibromo[1,2,5]thiadiazolo[3,4-*d*]pyridazine (**1**) was 36% that is almost three times higher than in the method described by us previously [22]. The other advantages are a shorter route, and the safety of the procedures which can be carried out on a larger synthetic scale as the use of the dangerous tetrasulfur tetranitride and chlorine is avoided.

### 2.1. Aromatic Nucleophilic Substitution in Bromo Derivatives of [1,2,5]thiadiazolo[3,4-d]pyridazine

The nucleophilic substitution in 3,6-dihalosubstituted 1,2-pyridazines fused with electron deficient heterocyclic rings (pyrazine [25], 1,2,3-triazole [26,27] and imidazole [28,29] was poorly investigated and did not give a clear picture on the possibilities for the selective replacement of one or two halogen(s). The situation is complicated due to the hydrolytic instability of 4,7-dibromo[1,2,5]thiadiazolo[3,4-*d*]pyridazine (**1**), and necessary precautions should be taken. The reactivity of oxygen, sulfur- and nitrogen-heteroatom nucleophiles towards dibromo derivative **1** was investigated systematically.

#### 2.1.1. Oxygen Nucleophiles

4,7-Dibromo[1,2,5]thiadiazolo[3,4-*d*]pyridazine (**1**) is readily hydrolyzed by water to give 7-bromo[1,2,5]thiadiazolo[3,4-*d*]pyridazin-4(5H)-one (**7**) (Table 1). 

The rate of the hydrolysis depends on the conditions used. If dibromo derivative **1** was stored in air at room temperature, it was completely converted to the monobromo derivative **7** in 30 days. In wet solvents (for example, in chloroform), the reaction occurred much faster (Entries 2, 3, Table 1). This means that for other reactions (nucleophilic substitution and cross-coupling reactions), it is strongly recommended to avoid even traces of water. Surprisingly, dibromothiadiazolopyridazine **1** gave in the reaction with MeOH the same 7-bromo[1,2,5]thiadiazolo[3,4-*d*]pyridazin-4(5H)-one (**7**) in high yield (Entry 6, Table 1). Less nucleophilic phenol did not react with dibromide **1** (Entries 10, 11, Table 1). Treatment of dibromide **1** with NaOMe in MeOH led selectively to displacement of one or two bromine atoms, depending on the quantity of the base used (Entries 7, 8, Table 1). Reaction of dibromide **1** with sodium phenoxide in THF gave monosubstituted product **8b** in high yields (Entries 12–14, Table 1). To obtain 4,7-diphenoxy[1,2,5]thiadiazolo[3,4-*d*]pyridazine (**9b**), it was necessary to use a strong aprotic dipolar solvent, viz., DMF, at 90 °C (Entry 17, Table 1). It is necessary to mention that monobromo derivatives **8** are hydrolytically stable and can be kept at room temperature for months without noticeable changes.

#### 2.1.2. Sulfur Nucleophiles

Dibromo derivative **1** successfully reacted upon treatment with thiophenol at room temperature in various organic solvents (CHCl_3_, THF, MeCN and DMF) to give dimercapto derivative **10a**, even if we used one equivalent of the thiol (Table 2). The reaction in an aprotic dipolar solvent (DMF) was facilitated and occurred much faster than in a less polar organic solvent (chloroform). The formation of the monosubstituted thiol was detected by TLC monitoring of the reaction mixtures in chloroform, and mono-adducts underwent fast transformation to dithiol **10a**.

Our attempts to isolate mono-substituted derivatives were unsuccessful; the use of one equivalent of a base and thiol led to a mixture of disubstituted derivative **10a** and starting material **1**. Inverse addition and lowering the concentration of the reagents did not also yield the monosubtituted products. Although the presence of a base did not facilitate the nucleophilic substitution by thiols (compare Entries 3 and 6, Table 2), bis(phenylthio) derivative **10a** was formed in a bit higher yield if sodium hydride was used. The reaction was extended to other thiols (hexanethiol and undecanothiol), and bis-thiols **10b**, **c** were isolated in high yields.

#### 2.1.3. Nitrogen Nucleophiles

Treatment of 4,7-dibromo[1,2,5]thiadiazolo[3,4-*d*]pyridazine (**1**) with morpholine in MeCN at room temperature for 3 h gave selectively mono-aminated derivative **11a** in good yield. This reaction was investigated thoroughly in order to achieve the best yield of this product (Table 3). It was found that the nature of the solvent did not affect the yield of the final product significantly, changing the reaction velocity only. According to TLC data (Silica), morpholine reacted with dibromo derivative **1** slower in MeOH (completed in 6 h, Table 3, Entry 1), and more rapidly in DMF (0.5 h, Table 3, Entry 9), and in all cases mono-aminated product **11a** was formed at room temperature. Disubstituted product **12a** was not detected even if an excess of morpholine was used.

Upon heating the reaction mixtures at 80 °C in DMF or in MeCN with two equivalents of morpholine in the presence of Et_3_N, diaminated product **12a** was formed. To complete the reaction in MeCN, the reaction mixture had to be refluxed for 30 h, whereas in DMF, heating for 20 h was required (Table 3, Entries 13, 14). The best yield and most convenient reaction conditions for the synthesis of unsymmetrical compound **11a** involved the treatment of dibromo derivative **1** with one equiv. of morpholine and Et_3_N in CH_2_Cl_2_ at room temperature, while for disubstituted compound **12a**—heating with two equivalents morpholine and Et_3_N in MeCN at 80 °C.

Furthermore, we explored the application of the optimized reaction conditions to other primary and secondary amines and achieved high yields of mono- and disubstituted thiadiazolopyridazines **11**-**12a–j** (Table 4). Carbazole and diphenylamine did not react with 4,7-dibromo-[1,2,5]thiadiazolo[3,4-*d*]pyridazine (**1**). Our attempts to force these reactions by using sodium salts of these amines (prepared in situ from carbazole or diphenylamine and NaH) were also unsuccessful—dibromothiadiazolopyridazine **1** decomposed and did not react with sodium carbazol-9-ide or sodium diphenylamide in THF or DMF even under heating (ca. 60 °C, 3 h).

**Scheme 3:**
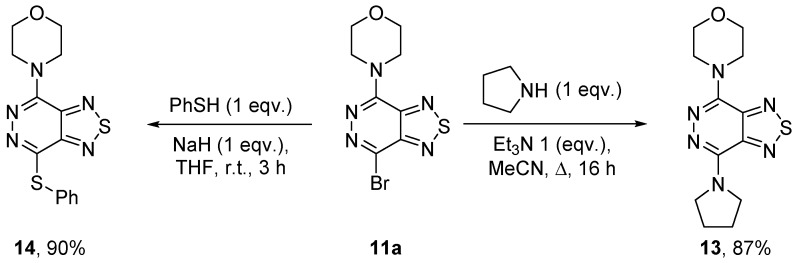
Synthesis of unsymmetrical disubstituted [1,2,5]thiadiazolo[3,4-*d*]pyridazines.

In order to obtain unsymmetrical disubstituted [1,2,5]thiadiazolo[3,4-*d*]pyridazines, the aromatic nucleophilic substitution in 4-(7-bromo[1,2,5]thiadiazolo[3,4-*d*]pyridazin-4-yl)morpholine (**11a**) was studied. It was found that the presence of a morpholine group did not affect the reactivity of the second bromine atom, which can be easily substituted by pyrrolidine or thiophenole under practically the same conditions as for dibromo derivative **1**, and unsymmetrical derivatives **13**, **14** were obtained in high yields (Scheme 3).

### 2.2. Cross-Couplings Based on the Buchwald-Hartwig and Ullmann Techniques—Synthesis of Mono- and Bis(9H-carbazol-9-yl)[1,2,5]thiadiazolo[3,4-d]pyridazines

It was found during our work that weak amines, such as carbazole and diphenylamine, did not react with bromo[1,2,5]thiadiazolo[3,4-*d*]pyridazines. Meanwhile, these two aminoaryl moieties have often been incorporated into benzothiadiazole molecules possessing high photovoltaic properties [30,31,32,33,34,35]. To introduce such amino building blocks into a heterocyclic ring, the Buchwald-Hartwig [30,34,35] and Ullmann [31,32,36,37] methodologies are often employed. The behavior of 4,7-dibromo[1,2,5]thiadiazolo[3,4-*d*]pyridazine (**1**) and 4-(7-bromo-[1,2,5]thiadiazolo[3,4-*d*]pyridazin-4-yl)morpholine (**11a**) was investigated using these protocols. 4,7-Dibromo[1,2,5]thiadiazolo[3,4-*d*]pyridazine (**1**) was found to be inert under all the conditions examined; treatment with diphenylamine or carbazole in the presence of Pd(0) catalysts (Pd(OAc)_2_, Pd_2_(dba)_3_), various ligands (dppf, BINAP, XPhos or Bu^t^_3_P) and Cs_2_CO_3_ in toluene or DMF at temperatures from 110 °C to 140 °C with prolonged (12–48 h) heating led to partial decomposition of the starting compound.

The reaction of 4-(7-bromo-[1,2,5]thiadiazolo[3,4-*d*]pyridazin-4-yl)morpholine (**11a**) with carbazole under Buchwald-Hartwig or Ullmann conditions resulted in 4-(7-(9*H*-carbazol-9-yl)-[1,2,5]thiadiazolo[3,4-*d*]pyridazin-4-yl)morpholine (**15**) in moderate to low yields. The results are summarized in Table 5. Refluxing of the reaction mixtures and microwave irradiation without any ligand were not successful, the starting material was recovered in high yield. Using XPhos ligand in the Buchwald-Hartwig reaction and DMEDA in the Ullmann reaction gave carbazole derivative **15**. In both cases, microwave irradiation gave better results than prolonged heating in the corresponding solvent (Table 5).

Diphenylamine was found to be inert to the treatment with 4-(7-bromo-[1,2,5]thiadiazolo[3,4-*d*]pyridazin-4-yl)morpholine (**11a**) even under these conditions.

**Scheme 4:**
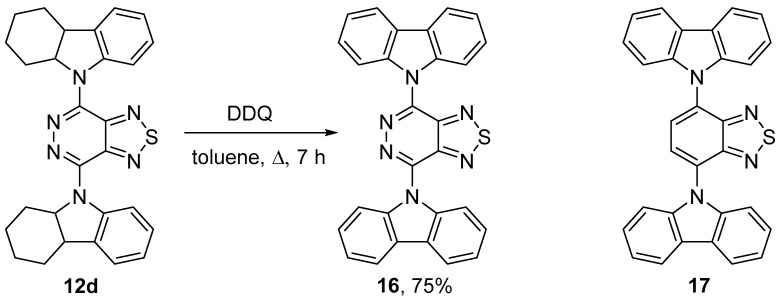
Synthetic route to 4,7-di(9*H*-carbazol-9-yl)[1,2,5]thiadiazolo[3,4-*d*]pyridazine (**16**).

Recently it was found that 4,7-di(9*H*-carbazol-9-yl)benzo[*c*][1,2,5]thiadiazole (**17**) can be considered as an excellent candidate for a highly efficient red thermally activated delayed fluorescence emitter (TADF), and can also act as a high efficiency organic light-emitting diode (OLED) [30], since this compound has a small energy gap which is compatible with a large fluorescence rate. 4,7-di(9*H*-Carbazol-9-yl)[1,2,5]thiadiazolo[3,4-*d*]pyridazine (**16**), which seems to be a promising candidate for a similar application, could not be obtained from dibromo derivative **1** because it did not react either under aromatic nucleophilic substitution conditions or under Buchwald-Hartwig or Ullmann conditions. We have found that this compound can be successfully obtained from its bis(hexahydrocarbazolyl) derivative **12d** by dehydrogenation with DDQ by a known procedure (see ref. [28]), affording the target product **16** in high yield (Scheme 4). The fluorescent and photophysical properties of the new OLED are now under investigation.

## 3. X-Ray Analysis

The geometry of the [1,2,5]thiadiazolo[3,4-*d*]pyridazine moiety in **10b** and **16** is rather close to the expected one (see ref. [31]) with the only exception concerning some shortening of the C(3A)-C(7A) bond. It should be noted that in [1,2,5]thiadiazolo[3,4-d]pyridazine (**10b**), the biscycle is planar, while in **16** it is bent with a dihedral angle of ~9.5° between the thiadiazole and pyridazine rings. Such a conformation of **16** can be the result of crystal packing effects.

**Figure 1:**
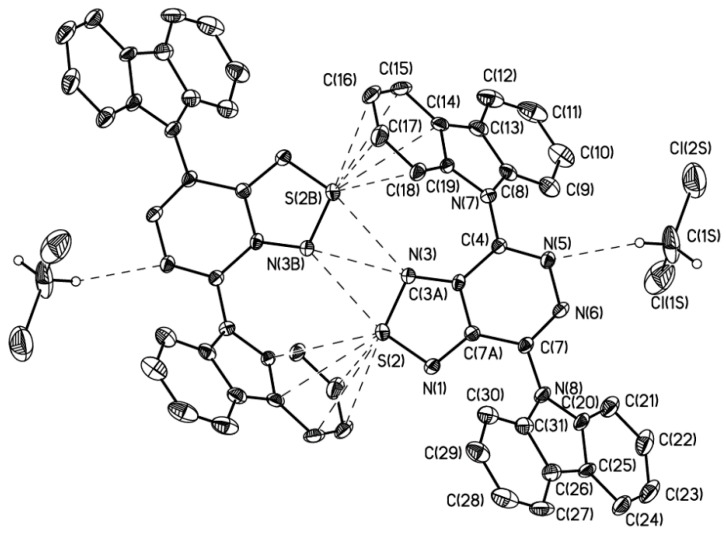
Dimers in the crystal of **16** in representation of atoms by thermal ellipsoids (*p* = 50%).

Indeed each of the independent molecules (A, B) in the crystal of **16** participates in the formation of almost identical centrosymmetric A…A and B…B dimers, in which the molecules are assembled by shortened S(2)…N(3) (S…N 3.203(2) Å, N(3)…S(2)-N(1) 164.0(1)°) and N(3)…N(3) (N…N 2.963(3), N(3)….N(3)-C(3A) 165.7(3)°) interactions as well as by the rather rare S…π interaction of η^6^-type (S…C 3.336(3)–3.455(3) Å, S…C_centroid_ 3.087(2)3) with the 6-membered carbazole ring (Figure 1). In turn, these A…A and A….B dimers are assembled into chains of the (A…A)…(B….B) type by stacking interactions between pyridazine (interplane distance 3.1 Å) and carbazole rings (3.2 and 3.4 Å). Finally, one of the pyridazine nitrogen atoms participates only in the weak C–H…N interaction (H…N 2.32 Å, CHN 148°) with a CH_2_Cl_2_ solvent molecule.

**Figure 2:**
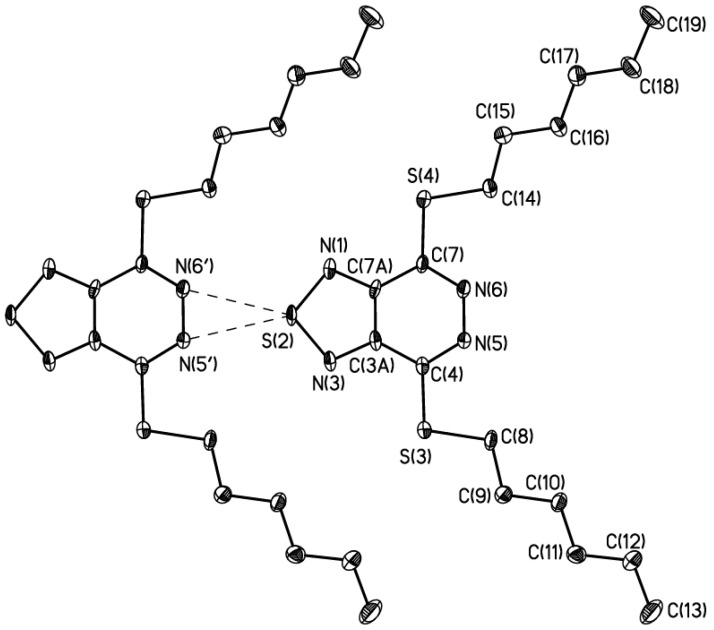
Fragment of the S…η^2^-(N=N) bound chains in a crystal of **10b** in representation of atoms by thermal ellipsoids (*p* = 50%).

In contrast, in the crystal of **10b**, the main “actors” in the self-assembly are the pyridazine nitrogen atoms which form very rare symmetrical S…η^2^-(N=N) interactions (S(2)…N(5) 2.992(2), S(2)…N(6) 3.015(2) Å, N(5)…S(2)N(1) 142.3(3), N(6)…S(2)N(3) 143.4(3)°) that bound molecules into almost planar chains (Figure 2). Like in **16**, there are two independent molecules (A, B) in the crystal of **10b**; each of them forms separate almost identical A…A…A and B…B…B planar chains. The S-alkyl substituents in **9b** are characterized by all-*trans* conformation, and this hydrophobic shell links the chains by weak C–H…π and C–H…H–C interactions into a 3-dimensional framework. One can expect that formation of such S…η^2^-(N=N) bound chains will be rather stable for various S-Alk substituents that make such compounds interesting for the design of liquid crystals.

To analyse the character of the shortened contacts in **10b** and **16**, we used the topological analysis of electron density function (ρ(**r**)) within Bader’s “atom in molecule theory” [38]. The ρ(**r**) function was obtained from the PBE/6-311++G* single point calculations (see Supplementary Materials) for the above mentioned dimers in the crystals of **10b** and **16**. According to the critical point (CP), search both S…N interaction are characterized by the presence of CP (3,−1) of ρ(**r**) and thus may be considered as attractive interactions (Figure S1) [38]. Their energies estimated by Espinosa et al. using the CEML method [39,40] are equal to ~3.0 kcal/mol each. Based on the analysis of the ELF function, we can consider them in terms of n-σ* interactions as the transfer from the nitrogen electron lone pairs to the S-N σ*-orbital.

In contrast, the same procedure for **16** revealed that only the N(3)…N(3) interaction is characterized by CP(3,−1). Furthermore, despite the η^6^-type of interaction of S(2) with the carbazole ring, only one S(2)…C(14) contact (3.340(2) Å) is characterized by the presence of CP(3,−1). According to the analysis of ELF, we can assume that this contact is the only one for which the electron lone pair of the S(2) atom has the appropriate orientation with respect to the carbons of the aromatic ring (Figure S4). The energies of N…N and S…C interactions are equal to 2.2 and 1.5 kcal/mol, respectively.

## 4. Experimental Section

### 4.1. General Information

Powdered anhydrous Na_2_SO_4_ was used for drying organic extracts and all volatiles were removed under reduced pressure. All reaction mixtures and column eluents were monitored by TLC using commercial aluminium backed thin layer chromatography (TLC) plates (Kieselgel 60 F_254_ Merck, Kenilworth, NJ, USA). The plates were observed under UV light at 254 nm. Melting points were determined on a Kofler hot-stage apparatus and are uncorrected. Solvents used for recrystallization are indicated after the melting point. ^1^H and ^13^C-NMR spectra were taken with a Bruker AM-300 machine (at 300.1 and 75.5 MHz) or Bruker DRX500 machine (at 500.1 and 125.8 MHz) or Bruker AV600 machine (at 600.1 and 150.9 MHz) with TMS as the standard (Bruker, Billerica, MA, USA). *J* values are given in Hz. MS spectra (EI, 70 eV) were obtained with a MAT INCOS 50 instrument (Thermo Finnigan LLC, San Jose, CA, USA). High-resolution MS spectra were measured on a Bruker MICROTOF II instrument using electrospray ionization (ESI, Bruker). The measurement was operated in a positive ion mode (interface capillary voltage −4500 V) or in a negative ion mode (3200 V); mass range was from *m*/*z* 50 to 3000 Da; external or internal calibration was done with Electrospray Calibrant Solution (Fluka Chemicals Ltd., Gillingham, UK). A syringe injection was used for solutions in acetonitrile, methanol, or water (flow rate 3 μL/min). Nitrogen was applied as a dry gas; interface temperature was set at 180 °C. IR spectra were measured with a Bruker “Alpha-T” instrument (Bruker) in KBr pellets. The reagents were purchased from commercial sources and used as received. All synthetic operations were performed under a dry argon atmosphere. Solvents were purified by distillation from the appropriate drying agents.

Data collection for single crystals of **10b** and **16** was performed with a Bruker Smart Apex II CCD diffractometer (MoKα radiation, λ = 0.71073 Å, graphite monochromator). Frames were integrated with the Bruker (2005) SAINT v7.23A software package using a narrow-frame algorithm, and a semiempirical absorption correction was applied with the SADABS program [41] using intensity data of the equivalent reflections. All the structures were solved by the direct method and refined by the least-squares in anisotropic full-matrix approximation on F^2^_hkl_. The OH hydrogen atoms were localized from differential Fourier-syntheses of electron density, and the rest ones were calculated geometrically and refined in isotropic approximation using the riding model with the SHELX software package [42]. Detailed crystallographic information is provided in Table 6 and as Electronic Supplementary Information in CIF format that can be obtained free of charge via http://www.ccdc.cam.ac.uk/conts/retrieving.html or from the Cambridge Crystallographic Data Centre, 12, Union Road, Cambridge CB2 1EZ, UK; fax: 44-1223-336033 using the reference CCDC numbers 1839518-1839519.

### 4.2. 4,7-Dibromo[1,2,5]thiadiazolo[3,4-d]pyridazine *(**1**)*

#### 4.2.1. 1,2,5-Thiadiazole-3,4-dicarbonitrile (**5**)

Thionyl chloride (1210 mg, 10.18 mmol) was added dropwise to a solution of diaminomaleonitrile (1.0 g, 9.26 mmol) in MeCN (10 mL) at 0 °C. The reaction mixture was stirred at room temperature for 3 h, solvent was evaporated. The residue was dissolved in CH_2_Cl_2_ (25 mL), washed by 1.5N HCl and dried over MgSO_4_. Evaporation of the solvent gave title compound **5** 1.0 g (79%) as a yellow solid. The spectral data correspond to the literature [23].

#### 4.2.2. 1,2,5-Thiadiazole-3,4-dicarboxylic Acid (**6**)

A mixture of 1,2,5-thiadiazole-3,4-dicarbonitrile (**5**, 2.5 g, 18.38 mmol) and 6N HCl (27 mL) was refluxed for 24 h and cooled to room temperature. The precipitate was filtered and dried. Yield of **6** 2.878 g (90%). The spectral data correspond to the literature [23].

#### 4.2.3. Dimethyl 1,2,5-thiadiazole-3,4-dicarboxylate (**2**)

Thionyl chloride (732 mg, 6.15 mmol) was added dropwise to a solution of 1,2,5-thiadiazole-3,4-dicarboxylic acid (**6**, 2.0 g, 11.49 mmol) in MeOH (20 mL) at 0 °C. The reaction mixture was refluxed for 4 h, the solvent was evaporated. The residue was dissolved in CH_2_Cl_2_ (40 mL), washed with water and dried over MgSO_4_. Evaporation of the solvent gave title compound **2** 1.80 g (77%) as a colourless oil. The spectral data correspond to the literature [43].

#### 4.2.4. [1,2,5]Thiadiazole-3,4-dicarboxylic Acid Dihydrazide (**3**)

To a solution of dimethyl 1,2,5-thiadiazole-3,4-dicarboxylate (**2**, 4.2 g, 0.02 mol) in ethanol (20 mL) 3.7 mL of 80% hydrazine hydrate was added. An orange-red solid immediately separated from the reaction mixture, which was set aside overnight at room temperature and then filtered to give [1,2,5]thiadiazole-3,4-dicarboxylic acid dihydrazide **3**. Yield 4.0 g (98%), orange solid. The spectral data correspond to the literature [24].

#### 4.2.5. 5,6-Dihydro[1,2,5]thiadiazolo[3,4-d]pyridazine-4,7-dione (**4**)

[1,2,5]Thiadiazole-3,4-dicarboxylic acid dihydrazide (**3**, 1.2 g, 0.006 mol) was added to 80% formic acid (20 mL). The mixture was heated under reflux for 5 h. After cooling the precipitate was filtered, dissolved in 5% aqueous sodium hydroxide and recrystallised from acetic acid to give 5,6-dihydro[1,2,5]thiadiazolo[3,4-*d*]pyridazine-4,7-dione (**4**). Yield 1.1 g (90%), white powder. Mp > 300 °C, lit. [44] Mp > 360 °C. IR *ν*_max_ (KBr, cm^−1^): 3381, 3283, 1670, 1630, 1440, 1389, 1270, 1107, 860, 522. ^1^H-NMR (300 MHz, DMSO-*d*_6_): δ 12.04 (2H, s). ^13^C-NMR (75 MHz, DMSO-*d*_6_): δ·150.23, 151.47. HRMS (ESI-TOF), *m*/*z*: calcd for C_4_H_2_N_4_O_2_S [M + H]^+^, 170.9971, found, 170.9976. MS (EI, 70 eV), *m*/*z* (*I*, %): 170 (M^+^, 64), 140 (6), 84 (23), 58 (34), 29 (100).

#### 4.2.6. 4,7-Dibromo[1,2,5]thiadiazolo[3,4-d]pyridazine (**1**)

5,6-Dihydro[1,2,5]thiadiazolo[3,4-*d*]pyridazine-4,7-dione (**4**, 650 mg, 3.82 mmol) was added to PBr_5_ formed by addition of bromine (1.18 mL, 22.92 mmol) to PBr_3_ (2.16 mL, 22.92 mmol) at 0 °C. The mixture was stirred for 9 h at 105 °C. The resulting mixture was cooled to the room temperature, poured into ice, washed with CCl_4_, extracted with CHCl_3_ (3 × 40 mL) and dried over MgSO_4_. The CHCl_3_ was evaporated under reduced pressure. The residue was purified by column chromatography (silica gel Merck 60, CH_2_Cl_2_) to give the title compound **1**. Yield 845 mg (75%), yellow solid, R_f_ = 0.5, CH_2_Cl_2_. Mp = 199–200 °C. IR *ν*_max_ (KBr, cm^−1^): 1369, 1361, 1343, 1257, 959, 863, 504. ^13^C-NMR (75 MHz, CDCl_3_): δ 142.5, 149.6. HRMS (ESI-TOF), *m*/*z*: calcd for C_4_^81^Br_2_HN_4_S [M + H]^+^, 296.8262, found, 296.8269. MS (EI, 70 eV), *m*/*z* (%): 298 ([M + 2]^+^, 22), 296 (M^+^, 49), 294 ([M − 2]^+^, 28), 217 (27), 215 (28), 136 (52), 84 (67), 32 (100).

### 4.3. Reaction of 4,7-dibromo[1,2,5]thiadiazolo[3,4-d]pyridazine *(**1**) with* H_2_O

To 4,7-dibromo[1,2,5]thiadiazolo[3,4-*d*]pyridazine (**1**, 50 mg, 0.17 mmol) was added H_2_O (6 mg, 0.34 mmol) in CHCl_3_ (15 mL). The reaction mixture was stirred at room temperature for 30 h. On completion (monitored by TLC), the mixture was poured into water, and extracted with EtOAc (2 × 25 mL). The extract was dried over MgSO_4_. The solvent was evaporated under reduced pressure. The residue was purified by column chromatography (silica gel Merck 60, EtOAc) to give 7-bromo[1,2,5]thiadiazolo[3,4-*d*]pyridazin-4(5H)-one (**7**). Yield 32 mg (82%), orange solid, R_f_ = 0.12 (CH_2_Cl_2_). Mp = 222–224 °C. IR *ν*_max_ (KBr, cm^−1^): 3189, 3147, 3073, 3030, 2991, 2886, 1691, 1669, 1450, 1419, 1289, 1157, 975, 859, 844, 675, 614, 508. ^1^H-NMR (300 MHz, DMSO-*d*_6_): δ 13.22 (s, 1H). ^13^C-NMR (75 MHz, DMSO-*d*_6_): δ 123.6, 150.5, 153.8, 156.8. HRMS (ESI-TOF), *m*/*z*: calcd. for C_4_H^79^BrN_4_OSNa [M + Na]^+^, 254.8947, found, 254.8939. MS (EI, 70 eV), *m*/*z* (*I*, %): 235 ([M + 2]^+^, 4), 234 ([M + 1]^+^, 100), 233 (M^+^, 3), 232 ([M − 1]^+^, 96), 177 (15), 125 (8), 46 (40).

### 4.4. Reaction of 4,7-dibromo[1,2,5]thiadiazolo[3,4-d]pyridazine *(**1**)* with MeOH

Dry MeOH (15 mL) was added to 4,7-dibromo[1,2,5]thiadiazolo[3,4-*d*]pyridazine (**1**, 50 mg, 0.17 mmol) in CHCl_3_ (15 mL). The reaction mixture was stirred at room temperature for 16 h. On completion (monitored by TLC), the mixture was poured into water and extracted with EtOAc (2 × 25 mL). The extract was dried over MgSO_4_. The solvent was evaporated under reduced pressure. The residue was purified by column chromatography (silica gel Merck 60, EtOAc) to give 7-bromo[1,2,5]thiadiazolo[3,4-*d*]pyridazin-4(5H)-one (**7**). Yield 30 mg (78%).

### 4.5. 4-Bromo-7-methoxy[1,2,5]thiadiazolo[3,4-d]pyridazine *(**8a**)*

4,7-Dibromo[1,2,5]thiadiazolo[3,4-*d*]pyridazine (**1**, 50 mg, 0.17 mmol) was added to a solution of MeONa (9 mg, 0.17 mmol) in dry MeOH (3 mL) at room temperature with stirring. The reaction mixture was stirred at room temperature for 6 h. On completion (monitored by TLC), poured into water (10 mL) and extracted with CH_2_Cl_2_ (3 × 30 mL). The combined organic layers were washed with brine, dried over MgSO_4_, filtered, and concentrated under reduced pressure. The crude product was purified by column chromatography (CH_2_Cl_2_/Hexane, 1:1, *v*/*v*) to afford 34 mg (80%) of target compound **8a** as a white solid, Rf = 0.5 (CH_2_Cl_2_). Mp = 145–147 °C. IR *ν*_max_ (KBr, cm^−1^): 2954, 1508, 1446, 1435, 1387, 1364, 1349, 1323, 1154, 955, 864, 676, 514. ^1^H-NMR (300 MHz, CDCl_3_): δ 4.38 (s, 3H). ^13^C-NMR (75 MHz, CDCl_3_): δ 56.3, 136.0, 143.3, 151.8, 158.1. HRMS (ESI-TOF), *m*/*z*: calcd. for C_5_H_4_^79^BrN_4_OS [M + H]^+^, 246.9284, found, 246.9272. MS (EI, 70 eV), *m*/*z* (*I*, %): 248 ([M + 1]^+^, 20), 247 (M^+^, 10), 246 ([M − 1]^+^, 19), 245 ([M − 2]^+^, 8), 137 (100), 85 (20), 46 (35).

### 4.6. 4-Bromo-7-phenoxy[1,2,5]thiadiazolo[3,4-d]pyridazine *(**8b**)*

Sodium hydride (3 mg, 0.13 mmol) was added to a solution of phenol (12 mg, 0.13 mmol) in dry THF (3 mL) at 0 °C with stirring. The reaction mixture was stirred at 0 °C for 30 min, then 4,7-dibromo[1,2,5]thiadiazolo[3,4-*d*]pyridazine (**1**, 40 mg, 0.13 mmol) was added. The mixture was stirred for 8 h at room temperature. On completion (monitored by TLC), the mixture was poured into water and extracted with EtOAc (3 × 30 mL). The combined organic layers were washed with brine, dried over MgSO_4_, filtered, and concentrated under reduced pressure. The crude product was purified by column chromatography (CH_2_Cl_2_) to afford 32 mg (80%) of target compound **8b** as a white solid, R_f_ = 0.7 (CH_2_Cl_2_). Mp = 106–108 °C. IR *ν*_max_ (KBr, cm^−1^): 1590, 1416, 1361, 1344, 1326, 1257, 1193, 1052, 959, 865, 798, 757, 701, 516, 506. ^1^H-NMR (300 MHz, CDCl_3_): δ 7.32–7.40 (m, 3H), 7.48–7.53 (m, 2H). ^13^C-NMR (75 MHz, CDCl_3_): δ 121.4, 126.4, 129.9, 137.0, 143.3, 152.3, 152.32, 158.1. HRMS (ESI-TOF), *m*/*z*: calcd. for C_10_H_6_^79^BrN_4_OS [M + H]^+^, 308.9440, found, 308.9448. MS (EI, 70 eV), *m*/*z* (*I*, %): 311 ([M + 2]^+^, 8), 310 ([M + 1]^+^, 75), 309 (M^+^, 72), 308 ([M − 1]^+^, 72), 307 ([M − 2]^+^, 12), 91 (100).

### 4.7. 4,7-Dimethoxy[1,2,5]thiadiazolo[3,4-d]pyridazine *(**9a**)*

4,7-Dibromo[1,2,5]thiadiazolo[3,4-*d*]pyridazine (**1**, 50 mg, 0.17 mmol) was added to a solution of MeONa (18 mg, 0.34 mmol) in dry MeOH (3 mL) at room temperature with stirring. The reaction mixture was stirred at room temperature for 24 h. On completion (monitored by TLC), the mixture was poured into water and extracted with CH_2_Cl_2_ (3 × 30 mL). The combined organic layers were washed with brine, dried over MgSO_4_, filtered, and concentrated under reduced pressure. The crude product was purified by column chromatography (CH_2_Cl_2_) to afford 28 mg (82%) of target compound **9a** as a white solid, R_f_ = 0.1 (CH_2_Cl_2_). Mp = 165–167 °C. IR *ν*_max_ (KBr, cm^−1^): 2957, 2853, 1487, 1408, 1368, 1346, 1254, 1187, 1094, 1072, 961, 862, 764, 684, 519. ^1^H-NMR (300 MHz, CDCl_3_): δ 4.28 (s, 6H). ^13^C-NMR (75 MHz, CDCl_3_): δ 55.4, 146.0, 155.4. HRMS (ESI-TOF), *m*/*z*: calcd for C_6_H_7_N_4_O_2_S [M + H]^+^, 199.0284, found, 199.0290. MS (EI, 70 eV), *m*/*z* (*I*, %): 199 ([M + 1]^+^, 10), 198 (M^+^, 98), 197 ([M − 1]^+^, 70).

### 4.8. 4,7-Diphenoxy[1,2,5]thiadiazolo[3,4-d]pyridazine *(**9b**)*

Sodium hydride (6 mg, 0.26 mmol) was added to a solution of phenol (24 mg, 0.26 mmol) in dry DMF (3 mL) at 0 °C with stirring. The reaction mixture was stirred at 0 °C for 30 min, then 4,7-dibromo[1,2,5]thiadiazolo[3,4-*d*]pyridazine (**1**, 40 mg, 0.13 mmol) was added. The mixture was stirred for 6 h at 90 °C. On completion (monitored by TLC), the mixture was poured into water (10 mL) and extracted with EtOAc (3 × 30 mL). The combined organic layers were washed with brine, dried over MgSO_4_, filtered, and concentrated under reduced pressure. The crude product was purified by column chromatography (CH_2_Cl_2_) to afford 29 mg (69%) of target compound **9b** as a white solid, R_f_ = 0.5 (CH_2_Cl_2_). Mp = 134–136 °C. IR *ν*_max_ (KBr, cm^−1^): 1458, 1439, 1368, 1261, 1247, 1188, 1088, 1069, 1026, 870, 810, 691, 518. ^1^H-NMR (300 MHz, CDCl_3_): δ 7.30–7.37 (m, 6H), 7.42–7.47 (m, 4H). ^13^C-NMR (75 MHz, CDCl_3_): δ 121.6, 126.0, 129.7, 146.3, 152.6, 155.9. HRMS (ESI-TOF), *m*/*z*: calcd. for C_16_H_11_N_4_O_2_S [M + H]^+^, 323.0597, found, 323.0592. MS (EI, 70 eV), *m*/*z* (*I*, %): 323 ([M + 1]^+^, 30), 322 (M^+^, 100), 321 ([M − 1]^+^, 32), 91 (15), 77 (31).

### 4.9. General Procedure for the Reaction of 4,7-dibromo[1,2,5]thiadiazolo[3,4-d]pyridazine *(**1**)* with Thiols

Sodium hydride (8 mg, 0.34 mmol) was added to a solution of thiol (0.34 mmol) in dry THF (15 mL) at 0 °C with stirring. The reaction mixture was stirred at 0 °C for 30 min, then 4,7-dibromo[1,2,5]thiadiazolo[3,4-*d*]pyridazine (**1**, 50 mg, 0.17 mmol) was added. The mixture was stirred for 3–4 h at room temperature. On completion (monitored by TLC), the mixture was poured into water (20 mL) and extracted with EtOAc (3 × 35 mL). The combined organic layers were washed with brine, dried over MgSO_4_, filtered, and concentrated under reduced pressure. The crude product was purified by column chromatography.

#### 4.9.1. 4,7-Bis(phenylthio)[1,2,5]thiadiazolo[3,4-d]pyridazine (**10a**)

Yellow solid, 51 mg (85%), R_f_ = 0.8 (CH_2_Cl_2_). Mp = 186–188 °C. Eluent—CH_2_Cl_2_/hexane, 1:1 (*v*/*v*). IR *ν*_max_ (KBr, cm^−1^): 3060, 3048, 1473, 1439, 1383, 1275, 1068, 1022, 978, 860, 750, 705, 688, 640, 564, 503. ^1^H-NMR (300 MHz, CDCl_3_): δ 7.42–7.45 (m, 6H), 7.68–7.71 (m, 4H). ^13^C-NMR (75 MHz, CDCl_3_): δ 126.4, 129.6, 129.9, 135.5, 147.9, 155.8. HRMS (ESI-TOF), *m*/*z*: calcd for C_16_H_11_N_4_S_3_ [M + H]^+^, 355.0140, found, 355.0138. MS (EI, 70 eV), *m*/*z* (*I*, %): 355 ([M + 1]^+^, 27), 354 (M^+^, 26), 353 ([M − 1]^+^, 98), 109(100).

#### 4.9.2. 4,7-Bis(hexylthio)[1,2,5]thiadiazolo[3,4-d]pyridazine (**10b**)

Yellow solid, 56 mg (90%), R_f_ = 0.7 (CH_2_Cl_2_). Mp = 81–83 °C. Eluent—CH_2_Cl_2_/hexane, 1:1 (*v*/*v*). IR *ν*_max_ (KBr, cm^−1^): 2954, 2925, 2858, 1468, 1395, 1286, 1258, 1208, 1042, 987, 852, 721, 644, 551, 511. ^1^H-NMR (300 MHz, CDCl_3_): δ 0.91 (t, *J* = 7.0 Hz, 6H), 1.32–1.38 (m, 8H), 1.51–1.57 (m, 4H), 1.86 (p, *J* = 7.4 Hz, 4H), 3.49 (t, *J* = 7.4 Hz, 4H). ^13^C-NMR (75 MHz, CDCl_3_): δ 14.0, 22.5, 28.5, 28.6, 29.7, 31.3, 148.0, 155.0. HRMS (ESI-TOF), *m*/*z*: calcd for C_16_H_26_N_4_S_3_Na [M + Na]^+^, 393.1212, found, 393.1221. MS (EI, 70 eV), *m*/*z* (*I*, %): 372 ([M + 2]^+^, 10), 371 ([M + 1]^+^, 48), 370 (M^+^, 65), 323 (68), 286 (98), 202 (100).

#### 4.9.3. 4,7-Bis(dodecylthio)[1,2,5]thiadiazolo[3,4-d]pyridazine (**10c**)

Green solid, 80 mg (88%), R_f_ = 0.65 (CH_2_Cl_2_). Mp = 89–91 °C. Eluent—CH_2_Cl_2_/hexane, 1:1 (*v*/*v*). IR *ν*_max_ (KBr, cm^−1^): 2955, 2921, 2852, 1471, 1396, 1286, 1270, 1242, 1215, 1192, 1033, 988, 852, 836, 717, 646, 552, 511. ^1^H-NMR (300 MHz, CDCl_3_): δ 0.87–0.92 (m, 6H), 1.28–1.34 (m, 32H), 1.39–1.62 (m, 6H), 1.84–1.89 (m, 2H), 2.50–2.58 (m, 2H), 3.47–3.53 (m, 2H). ^13^C-NMR (75 MHz, CDCl_3_): δ 14.2, 22.7, 24.7, 28.4, 29.1, 29.4, 29.6, 29.68, 29.7, 29.73, 32.0, 34.1, 148.1, 155.1. HRMS (ESI-TOF), *m*/*z*: calcd. for C_28_H_51_N_4_S_3_ [M + H]^+^, 539.3270, found, 539.3259. MS (EI, 70 eV), *m*/*z* (*I*, %): 540 ([M + 2]^+^, 10), 539 ([M + 1]^+^, 28), 538 (M^+^, 30), 370 (40), 337 (76), 43 (100).

### 4.10. General Procedure for the Preparation of Mono-Aminated Products ***11***

Amine (0.17 mmol) and Et_3_N (17mg, 0.17 mmol) were added to a solution of 4,7-dibromo[1,2,5]thiadiazolo[3,4-*d*]pyridazine (**1**, 50 mg, 0.17 mmol) in dry CH_2_Cl_2_ (10 mL) at room temperature with stirring. The mixture was stirred at room temperature for 4 h. Then the mixture was poured into water (10 mL) and extracted with CH_2_Cl_2_ (3 × 35 mL). The combined organic layers were washed with brine, dried over MgSO_4_, filtered, and concentrated under reduced pressure. The crude product was purified by column chromatography.

#### 4.10.1. 4-(7-Bromo[1,2,5]thiadiazolo[3,4-d]pyridazin-4-yl)morpholine (**11a**)

Orange solid, 44 mg (86%), R_f_ = 0.2 (CH_2_Cl_2_). Mp = 158–160 °C. Eluent—CH_2_Cl_2_/EtOAc, 1:1 (*v*/*v*). IR *ν*_max_ (KBr, cm^−1^): 2972, 2924, 2868, 1541, 1463, 1445, 1422, 1299, 1276, 1253, 1113, 1028, 953, 892, 503. ^1^H-NMR (300 MHz, CDCl_3_): δ 3.91–3.94 (m, 4H), 4.34–4.38 (m, 4H). ^13^C-NMR (75 MHz, CDCl_3_): δ 47.0, 66.8, 130.7, 143.7, 151.2, 152.3. HRMS (ESI-TOF), *m*/*z*: calcd. for C_8_H_9_^79^BrN_5_OS [M + H]^+^, 301.9706, found, 301.9704. MS (EI, 70 eV), *m*/*z* (*I*, %): 304([M + 2]^+^, 9), 303 ([M + 1]^+^, 45), 302 (M^+^, 8), 301 ([M − 1]^+^, 44), 222 (100), 137(30).

#### 4.10.2. 4-Bromo-7-(piperidin-1-yl)[1,2,5]thiadiazolo[3,4-d]pyridazine (**11b**)

Yellow solid, 41 mg (82%), R_f_ = 0.2 (CH_2_Cl_2_). Mp = 116–118 °C. Eluent—CH_2_Cl_2_/EtOAc, 1:1 (*v*/*v*). IR *ν*_max_ (KBr, cm^−1^): 2940, 2924, 2858, 1539, 1462, 1451, 1424, 1284, 1267, 1223, 1131, 1025, 952, 889, 848, 504. ^1^H-NMR (300 MHz, CDCl_3_): 1.76–1.80 (m, 6H), 4.29–4.32 (m, 4H). ^13^C-NMR (75 MHz, CDCl_3_): δ 24.6, 26.1, 48.0, 129.2, 143.8, 151.1, 152.3. HRMS (ESI-TOF), *m*/*z*: calcd. for C_9_H_11_^79^BrN_5_S [M + H]^+^, 299.9913, found, 299.9917. MS (EI, 70 eV), *m*/*z* (*I*, %): 302 ([M + 2]^+^, 6), 301 ([M + 1]^+^, 45), 300 (M^+^, 5). 299 ([M − 2]^+^, 44), 220 (100), 84 (43).

#### 4.10.3. 4-Bromo-7-(pyrrolidin-1-yl)[1,2,5]thiadiazolo[3,4-d]pyridazine (**11c**)

Yellow solid, 39 mg (81%), R_f_ = 0.2 (CH_2_Cl_2_). Mp = 155–157 °C. Eluent—CH_2_Cl_2_/EtOAc, 1:1 (*v*/*v*). IR *ν*_max_ (KBr, cm^−1^): 2953, 2923, 2863, 1548, 1454, 1420, 1292, 1230, 1154, 1057, 959, 903, 861, 840, 505. ^1^H-NMR (300 MHz, CDCl_3_): 2.04–2.23 (m, 4H), 3.83–4.36 (m, 4H). ^13^C-NMR (75 MHz, CDCl_3_): δ 29.7, 49.4, 128.0, 144.1, 150.8, 151.1. HRMS (ESI-TOF): calcd for C_8_H_9_^79^BrN_5_S [M + H]^+^, 285.9757, found, 285.9759. MS (EI, 70 eV), *m*/*z* (*I*, %):286 ([M + 2]^+^, 8), 287 ([M + 1]^+^, 85), 286 (M^+^, 80). 208 (90), 137 (30), 70 (100).

#### 4.10.4. 4-Bromo-7-(2,3,4,4a-hexahydro-1H-carbazol-9(9aH)-yl)[1,2,5]thiadiazolo[3,4-d]pyridazine (**11d**)

Red solid, 56 mg (85%), R_f_ = 0.4 (CH_2_Cl_2_). Mp = 196–198 °C. Eluent—CH_2_Cl_2_/hexane, 1:1 (*v*/*v*). IR *ν*_max_ (KBr, cm^−1^): 2923, 2856, 1511, 1459, 1409, 1355, 1309, 1270, 1088, 880, 751, 506. ^1^H-NMR (300 MHz, CDCl_3_): δ 1.33–1.42 (m, 3H), 1.66–1.69 (m, 2H), 1.91–2.02 (m, 1H), 2.16–2.20 (m, 1H), 2.46 (d, *J* = 14.0 Hz, 1H), 3.66–3.71 (m, 1H), 5.73–5.81 (m, 1H), 7.17 (t, *J* = 7.3 Hz, 1H), 7.30 (d, *J* = 7.3 Hz, 1H). 7.33 (t, *J* = 8.1 Hz, 1H), 8.71 (d, *J* = 8.1 Hz, 1H). ^13^C-NMR (75 MHz, CDCl_3_): δ 20.9, 22.8, 24.2, 28.1, 40.2, 63.6, 120.4, 122.5, 124.5, 127.5, 130.8, 135.1, 142.7, 143.3, 149.8, 151.1. HRMS (ESI-TOF), *m*/*z*: calcd. for C_16_H_15_^79^BrN_5_S [M + H]^+^, 388.0226, found, 388.0229. MS (EI, 70 eV), *m*/*z* (*I*, %): 390 ([M + 2]^+^, 55), 389 ([M + 1]^+^, 70), 388 (M^+^, 55), 387 ([M − 1]^+^, 68), 308 (90), 172 (100), 130 (80).

#### 4.10.5. 4-Bromo-7-(1,3,3a,8b-tetrahydrocyclopenta[b]indol-4(2H)-yl)[1,2,5]thiadiazolo[3,4-d]pyridazine (**11e**)

Red solid, 52 mg (82%), R_f_ = 0.4 (CH_2_Cl_2_). Mp = 178–180 °C. Eluent—CH_2_Cl_2_/hexane, 1:1 (*v*/*v*). IR *ν*_max_ (KBr, cm^−1^): 2960, 2924, 2855, 1507, 1457, 1410, 1364, 1307, 1262, 1088, 884, 749, 509. ^1^H-NMR (300 MHz, CDCl_3_): 1.43–1.52 (m, 1H), 1.71–1.82 (m, 2H), 2.07–2.31 (m, 3H), 4.07–4.13 (m, 1H), 5.94–6.02 (m, 1H), 7.15 (t, *J* = 7.3 Hz, 1H), 7.28 (d, *J* = 7.3 Hz, 1H), 7.31 (t, *J* = 8.1 Hz, 1H), 8.85 (d, *J* = 8.1 Hz, 1H). ^13^C-NMR (75 MHz, CDCl_3_): δ 23.7, 34.1, 36.7, 45.8, 67.6, 118.9, 124.0, 124.5, 127.6, 130.9, 136.2, 143.5, 143.7, 150.0, 151.0. HRMS (ESI-TOF), *m*/*z*: calcd for C_15_H_13_^79^BrN_5_S [M + H]^+^, 374.0070, found, 374.0063. MS (EI, 70 eV), *m*/*z* (*I*, %): 376 ([M + 2]^+^, 8), 375 ([M + 1]^+^, 26), 374 (M^+^, 10), 373 ([M − 1]^+^, 25), 294 (40), 277 (45), 155 (55). 130 (100).

#### 4.10.6. 4-Bromo-7-(2,3,4,4a-tetrahydro-1H-1,4-methanocarbazol-9(9aH)-yl)-[1,2,5]thiadiazolo[3,4- d]pyridazine (**11f**)

Red solid, 53 mg (79%), R_f_ = 0.4 (CH_2_Cl_2_). Mp = 187–189 °C. Eluent—CH_2_Cl_2_/hexane, 1:1 (*v*/*v*). IR *ν*_max_ (KBr, cm^−1^): 2949, 2871, 1505, 1459, 1409, 1366, 1303, 1250, 1163, 1089, 884, 774, 508. ^1^H-NMR (300 MHz, CDCl_3_): δ 1.08 (d, *J* = 10.3 Hz, 1H), 1.35 (d, *J* = 9.8 Hz, 1H), 1.53–1.76 (m, 4H), 2.41 (d, *J* = 26.5 Hz, 2H), 3.51 (d, *J* = 7.5 Hz, 1H), 5.35 (d, *J* = 7.1 Hz, 1H), 7.05 (t, *J* = 7.3 Hz, 1H), 7.21 (d, *J* = 7.3 Hz, 1H), 7.26 (t, *J* = 8.1 Hz, 1H), 8.80 (d, *J* = 8.1 Hz, 1H). ^13^C-NMR (75 MHz, CDCl_3_): δ 25.4, 27.9, 31.8, 43.5, 43.6, 50.5, 69.4, 118.6, 124.2, 124.3, 127.7, 131.0, 135.2, 143.6, 144.8, 150.1, 150.9. HRMS (ESI-TOF), *m*/*z*: calcd for C_17_H_15_^79^BrN_5_S [M + H]^+^, 400.0226, found, 400.0232. MS (EI, 70 eV), *m*/*z* (*I*, %): 402 ([M + 2]^+^, 6), 401 ([M + 1]^+^, 24), 400 (M^+^, 5), 399 ([M − 1]^+^, 20), 320 (30), 184 (100), 143 (48), 116 (70).

#### 4.10.7. 7-Bromo-*N*-methyl-*N*-phenyl[1,2,5]thiadiazolo[3,4-d]pyridazin-4-amine (**11g**)

Red solid, 43 mg (80%), R_f_ = 0.3 (CH_2_Cl_2_). Mp = 188–190 °C. Eluent—CH_2_Cl_2_/hexane, 1:1 (*v*/*v*). IR *ν*_max_ (KBr, cm^−1^): 2955, 2924, 2852, 1538, 1524, 1494, 1422, 1391, 1361, 1345, 1311, 1282, 1198, 1064, 866, 777, 704, 564, 513. ^1^H-NMR (300 MHz, CDCl_3_): δ 3.76 (s, 3H), 7.20–7.33 (m, 2H), 7.43–7.46 (m, 3H). ^13^C-NMR (75 MHz, CDCl_3_): δ 41.9, 127.0, 127.7, 129.8, 131.4, 143.8, 145.8, 151.1, 152.5. HRMS (ESI-TOF), *m*/*z*: calcd for C_11_H_9_^79^BrN_5_S [M + H]^+^, 321.9757, found, 321.9752. MS (EI, 70 eV), *m*/*z* (*I*, %): 323 ([M + 1]^+^, 18), 322 (M^+^, 98), 321 ([M − 1]^+^, 15), 320 ([M − 2]^+^, 100), 77 (6), 28 (8).

#### 4.10.8. 7-Bromo-*N*-cyclohexyl[1,2,5]thiadiazolo[3,4-d]pyridazin-4-amine (**11h**)

Yellow solid, 45 mg (85%), R_f_ = 0.1 (CH_2_Cl_2_). Mp = 125–127 °C. Eluent—CH_2_Cl_2_/EtOAc, 1:1 (*v*/*v*). IR *ν*_max_ (KBr, cm^−1^): 2933, 2852, 1568, 1522, 1448, 1415, 1386, 1314, 1143, 1091, 960, 864, 835, 510. ^1^H-NMR (300 MHz, CDCl_3_): 1.28–1.50 (m, 5H), 1.70–1.82 (m, 3H), 2.12–2.41 (m, 2H), 4.24–4.45 (m, 1H), 5.66–5.90 (m, 1H). ^13^C-NMR (75 MHz, CDCl_3_): δ 24.7, 25.5, 32.6, 50.3, 129.2, 143.6, 149.9, 150.8. HRMS (ESI-TOF), *m*/*z*: calcd for C_10_H_13_^79^BrN_5_S [M + H]^+^, 314.0070, found, 314.0067. MS (EI, 70 eV), *m*/*z* (*I*, %): 316 ([M + 2]^+^, 5), 315 ([M + 1]^+^, 45), 314 (M^+^, 44).234 (100), 137 (3), 55 (15), 18 (46).

#### 4.10.9. 7-Bromo-*N*-phenyl[1,2,5]thiadiazolo[3,4-d]pyridazin-4-amine (**11i**)

Red solid, 41 mg (80%), R_f_ = 0.2 (CH_2_Cl_2_). Mp = 144–146 °C. Eluent—CH_2_Cl_2_/EtOAc, 1:1 (*v*/*v*). IR *ν*_max_ (KBr, cm^−1^): 2924, 1601, 1569, 1531, 1485, 1419, 1338, 1130, 872, 758, 501. ^1^H-NMR (300 MHz, DMSO-*d*_6_): δ 7.12 (t, *J* = 7.2 Hz, 1H), 7.41 (dd, *J* = 7.7, 7.2 Hz, 2H), 8.07 (d, *J* = 7.7 Hz, 2H), 10.27 (s, 1H). ^13^C-NMR (75 MHz, DMSO-*d*_6_): δ 119.9, 121.7, 123.9, 129.0, 131.5, 139.5, 144.2, 150.7. HRMS (ESI-TOF), *m*/*z*: calcd for C_10_H_7_^79^BrN_5_S [M + H]^+^, 307.9604, found, 307.9600. MS (EI, 70 eV), *m*/*z* (*I*, %): 309 ([M + 1]^+^, 24), 308 (M^+^, 100), 307 ([M − 1]^+^, 22), 306 ([M − 2]^+^, 90), 247 (46).

#### 4.10.10. 7-Bromo-*N*-(tert-butyl)[1,2,5]thiadiazolo[3,4-d]pyridazin-4-amine (**11j**)

Yellow solid, 36 mg (75%), R_f_ = 0.2 (CH_2_Cl_2_). Mp = 119–121 °C. Eluent—CH_2_Cl_2_/EtOAc, 1:1 (*v*/*v*). IR *ν*_max_ (KBr, cm^−1^): 2966, 2853, 1570, 1528, 1472, 1418, 1390, 1358, 1224, 1213, 1155, 1075, 957, 507. ^1^H-NMR (300 MHz, DMSO-*d*_6_): δ 1.65 (s, 9H), 5.80 (s, 1H). ^13^C-NMR (75 MHz, DMSO-*d*_6_): δ 28.6, 53.5, 129.5, 144.1, 149.8, 150.9. HRMS (ESI-TOF), *m*/*z*: calcd for C_8_H_11_^79^BrN_5_S [M + H]^+^, 287.9913, found, 287.9906. MS (EI, 70 eV), *m*/*z* (*I*, %): 290 ([M + 2]^+^, 25), 289 ([M + 1]^+^, 28), 288 (M^+^, 8), 287 ([M − 1]^+^, 40), 231 (100), 152 (60), 57 (80).

### 4.11. General Procedure for the Preparation of Di-Aminated Products ***12***

Amine (0.34 mmol) and Et_3_N (34 mg, 0.34 mmol) were added with stirring to a solution of 4,7-dibromo[1,2,5]thiadiazolo[3,4-*d*]pyridazine (**1**, 50 mg, 0.17 mmol) in dry MeCN (15 mL). The mixture was stirred at reflux for 10–30 h. Then the mixture was poured into water (25 mL) and extracted with EtOAc (3 × 35 mL). The combined organic layers were washed with brine, dried over MgSO_4_, filtered, and concentrated under reduced pressure. The crude product was purified by column chromatography.

#### 4.11.1. 4,7-Dimorpholino[1,2,5]thiadiazolo[3,4-d]pyridazine (**12a**)

Dark red solid, 45 mg (87%), R_f_ = 0.1 (CH_2_Cl_2_). Mp = 235–237 °C. Eluent—CH_2_Cl_2_/EtOAc, 1:2 (*v*/*v*). IR *ν*_max_ (KBr, cm^−1^): 2967, 2927, 2853, 1469, 1445, 1363, 1315, 1276, 1264, 1113, 910, 891, 526, 503. ^1^H-NMR (300 MHz, CDCl_3_): δ 3.90–3.93 (m, 8H), 3.99–4.03 (m, 8H). ^13^C-NMR (75 MHz, CDCl_3_): δ 47.8, 66.8, 146.6, 150.4. HRMS (ESI-TOF), *m*/*z*: calcd for C_12_H_17_N_6_O_2_S [M + H]^+^, 309.1128, found, 309.1128. MS (EI, 70 eV), *m*/*z* (*I*, %): 309 ([M + 2]^+^, 23), 308 (M^+^, 100), 307 ([M − 1]^+^, 50), 251 (25), 147 (12), 73 (5).

#### 4.11.2. 4,7-Di(piperidin-1-yl)[1,2,5]thiadiazolo[3,4-d]pyridazine (**12b**)

Dark red solid, 44 mg (85%), R_f_ = 0.1 (CH_2_Cl_2_). Mp = 104–106 °C. Eluent—CH_2_Cl_2_/EtOAc, 1:2 (*v*/*v*). IR *ν*_max_ (KBr, cm^−1^): 2918, 2846, 1463, 1435, 1304, 1233, 990, 938, 871, 554. ^1^H-NMR (300 MHz, CDCl_3_): 1.69–1.83 (m, 12H), 3.86–4.01 (m, 8H). ^13^C-NMR (75 MHz, CDCl_3_): δ 24.9, 25.9, 48.8, 147.1, 150.7. HRMS (ESI-TOF), *m*/*z*: calcd for C_14_H_21_N_6_S [M + H]^+^, 305.1543, found, 305.1541. MS (EI, 70 eV), *m*/*z* (*I*, %): 306 ([M + 2]^+^, 20), 305 ([M + 1]^+^, 98), 304 (M^+^, 100), 228 (70), 84 (80).

#### 4.11.3. 4,7-Bis(2,3,4,4a-tetrahydro-1H-carbazol-9(9aH)-yl)[1,2,5]thiadiazolo[3,4-d]pyridazine (**12d**)

Violet solid, 73 mg (90%), R_f_ = 0.6 (CH_2_Cl_2_). Mp = 239–241 °C. Eluent—CH_2_Cl_2_/Hexane, 1:1 (*v*/*v*). IR *ν*_max_ (KBr, cm^−1^): 2927, 2853, 1526, 1475, 1435, 1270, 1164, 1132, 877, 752, 574. ^1^H-NMR (300 MHz, CDCl_3_): δ 1.36–1.54 (m, 6H), 1.61–1.64 (m, 4H), 1.91–2.00 (m, 2H), 2.08–2.14, 2.37 (d, *J* = 13.2 Hz, 2H), 3.63–3.67 (m, 2H), 5.50–5.59 (m, 2H), 7.05 (t, *J* = 7.3 Hz, 2H), 7.24–7.29 (m, 4H), 8.27 (d, *J* = 8.1 Hz, 2H). ^13^C-NMR (75 MHz, CDCl_3_): δ 21.3, 22.6, 24.9, 27.7, 40.3, 63.4, 117.2, 122.0, 122.3, 127.0, 134.3, 144.2, 146.1, 147.0. HRMS (ESI-TOF), *m*/*z*: calcd for C_28_H_29_N_6_S [M + H]^+^, 481.2169, found, 481.2150. MS (EI, 70 eV), *m*/*z* (*I*, %): 482 ([M + 2]^+^, 8), 481 ([M + 1]^+^, 30), 480 (M^+^, 100), 172 (60), 130 (50).

#### 4.11.4. *N*^4^,*N*^7^-Diphenyl[1,2,5]thiadiazolo[3,4-d]pyridazine-4,7-diamine (**12i**)

Dark red solid, 42 mg (78%), R_f_ = 0.1 (CH_2_Cl_2_). Mp = 249–251 °C. Eluent—CH_2_Cl_2_/EtOAc, 1:2 (*v*/*v*). IR *ν*_max_ (KBr, cm^−1^): 2916, 2852, 1594, 1544, 1497, 1457, 1434, 1261, 1243, 882, 751, 687, 499. ^1^H-NMR (300 MHz, DMSO-*d*_6_): δ 7. 13 (t, *J* = 7.2 Hz, 2H), 7.31 (s, 2H), 7.44 (dd, *J* = 7.2, 7.4 Hz, 5H), 7.98 (d, *J* = 7.4 Hz, 3H). ^13^C-NMR (75 MHz, DMSO-*d*_6_): δ 119.3, 122.8, 129.2, 139.2, 145.4, 145.6. HRMS (ESI-TOF), *m*/*z*: calcd. for C_16_H_13_N_6_S [M + H]^+^, 321.0917, found, 321.0917. MS (EI, 70 eV), *m/z* (*I*, %): 321 ([M + 1]^+^, 52), 320 (M^+^, 65), 319 ([M − 1]^+^, 100). 144 (26), 77 (52).

### 4.12. 4-(7-(Pyrrolidin-1-yl)[1,2,5]thiadiazolo[3,4-d]pyridazin-4-yl)morpholine *(**13**)*

Amine **10c** (11.3 mg, 0.16 mmol) and Et_3_N (16.6 mg, 0.16 mmol) were added to a solution of 4-(7-bromo-[1,2,5]thiadiazolo[3,4-*d*]pyridazin-4-yl)morpholine (**11a**, 50 mg, 0.17 mmol) in dry MeCN (10 mL) at room temperature with stirring. The mixture was stirred at reflux for 16 h, then poured into water (20 mL) and extracted with CH_2_Cl_2_ (3 × 35 mL). The combined organic layers were washed with brine, dried over MgSO_4_, filtered, and concentrated under reduced pressure. The crude product was purified by column chromatography (CH_2_Cl_2_/EtOAc, 1:1, *v*/*v*) to afford 40 mg (87%) of target compound **13** as a dark red solid, R_f_ = 0.1 (CH_2_Cl_2_). Mp = 199–201 °C. IR *ν*_max_ (KBr, cm^−1^): 2977, 2919, 2862, 2821, 1551, 1472, 1445, 1342, 1330, 1261, 1243, 1117, 1024, 918, 890, 518. ^1^H-NMR (300 MHz, CDCl_3_): δ 2.04–2.09 (m, 4H), 3.83–3.86 (m, 4H), 3.91–3.94 (m, 4H), 3.98–4.03 (m, 4H). ^13^C-NMR (75 MHz, CDCl_3_): δ 25.5, 48.4, 48.9, 66.9, 146.7, 146.8, 148.9, 149.0. HRMS (ESI-TOF), *m*/*z*: calcd. for C_12_H_17_N_6_OS [M + H]^+^, 293.1179, found, 293.1171. MS (EI, 70 eV), *m*/*z* (*I*, %): 293 ([M + 1]^+^, 15), 292 (M^+^, 100), 291 ([M − 1]^+^, 48). 263 (43), 234 (48), 70 (80), 41 (60).

### 4.13. 4-(7-(Phenylthio)[1,2,5]thiadiazolo[3,4-d]pyridazin-4-yl)morpholine *(**14**)*

Sodium hydride (3.8 mg, 0.16 mmol) was added to a solution of thiol **8a** (18 mg, 0.17 mmol) in dry THF (10 mL) at 0 °C with stirring. The reaction mixture was stirred at 0 °C for 30 min, then 4-(7-bromo-[1,2,5]thiadiazolo[3,4-d]pyridazin-4-yl)morpholine (**11a**, 50 mg, 0.16 mmol) was added. The mixture was stirred for 3 h at room temperature. On completion (monitored by TLC), the mixture was poured into water and extracted with EtOAc (3 × 35 mL). The combined organic layers were washed with brine, dried over MgSO_4_, filtered, and concentrated under reduced pressure. The crude product was purified by column chromatography (CH_2_Cl_2_/Hexane, 2:1, *v*/*v*) to afford 43 mg (90%) of target compound **14** as a dark red solid, R_f_ = 0.3 (CH_2_Cl_2_). Mp = 142–144 °C. IR *ν*_max_ (KBr, cm^−1^): 2957, 2923, 2871, 1520, 1442, 1421, 1305, 1277, 1263, 1112, 1065, 1028, 977, 896, 863, 757, 672, 504. ^1^H-NMR (300 MHz, CDCl_3_): δ 3.87–3.90 (m, 4H), 4.24–4.27 (m, 4H), 7.37–7.43 (m, 3H), 7.65–7.68 (m, 2H). ^13^C-NMR (75 MHz, CDCl_3_): δ 47.0, 66.8, 128.4, 129.1, 129.3, 134.7, 143.7, 148.3, 151.0, 151.5. HRMS (ESI-TOF), *m*/*z*: calcd for C_14_H_14_N_5_OS_2_ [M + H]^+^, 332.0634, found, 332.0629. MS (EI, 70 eV), *m*/*z* (*I*, %): 332 ([M + 1]^+^, 25), 331 (M^+^, 52), 330 ([M − 1]^+^, 100), 222 (65), 86 (77).

### 4.14. 4-(7-(9H-Carbazol-9-yl)[1,2,5]thiadiazolo[3,4-d]pyridazin-4-yl)morpholine *(**15**)*

#### 4.14.1. By Buchwald-Hartwig Reaction

A mixture of 4-(7-bromo-[1,2,5]thiadiazolo[3,4-*d*]pyridazin-4-yl)morpholine (**11a**, 50 mg, 0.17 mmol), carbazole (43 mg, 0,25 mmol), Cs_2_CO_3_ (110 mg, 0.34 mmol), Pd(OAc)_2_ (10% mmol), and XPhos (5% mmol) in toluene (3 mL) was degassed by argon in a microwave vial. The resulting mixture was heated under microwave irradiation at 111 °C for 30 min. On completion (monitored by TLC), the mixture was poured into water and extracted with CH_2_Cl_2_ (3 × 25 mL). The combined organic layers were washed with brine, dried over MgSO_4_, filtered, and concentrated under reduced pressure. The crude product was purified by column chromatography (CH_2_Cl_2_/Hexane, 2:1, *v*/*v*) to afford 26 mg (40%) of target compound **15** as a dark red solid, R_f_ = 0.4 (CH_2_Cl_2_). Mp = 182–184 °C. IR *ν*_max_ (KBr, cm^−1^): 2956, 2924, 2853, 1541, 1534, 1491, 1467, 1446, 1335, 1298, 1277, 1260, 1230, 1116, 1026, 908, 885, 751, 723, 509. ^1^H-NMR (300 MHz, CDCl_3_): δ 4.02–4.05 (m, 4H), 4.50–4.54 (m, 4H), 7.35–7.48 (m, 6H), 8.18 (d, *J* = 7.1 Hz, 2H). ^13^C-NMR (75 MHz, CDCl_3_): δ 47.1, 67.0, 111.6, 120.3, 121.2, 124.5, 126.0, 140.3, 142.6, 145.4, 148.6, 152.1. HRMS (ESI-TOF), *m*/*z*: calcd for C_20_H_17_N_6_OS [M + H]^+^, 389.1179, found, 389.1180. MS (EI, 70 eV), *m*/*z* (*I*, %): 390 ([M + 2]^+^, 10), 389 ([M + 1]^+^, 65), 388 (M^+^, 100), 387 ([M − 1]^+^, 12), 331 (45), 222 (11).

#### 4.14.2. By Ullmann Reaction

A mixture of 4-(7-bromo-[1,2,5]thiadiazolo[3,4-*d*]pyridazin-4-yl)morpholine (**11a**, 50 mg, 0.17 mmol), carbazole (43 mg, 0.25 mmol), CuI (3 mg, 0.017 mmol), DMEDA (0.7 mg, 0.0085 mmol) in dioxane (3 mL) and water (1 mL) was degassed by argon in a microwave vial. The resulting mixture was heated under microwave irradiation at 100 °C for 10 min. On completion (monitored by TLC), the mixture was poured into water and extracted with CH_2_Cl_2_ (3 × 25 mL). The combined organic layers were washed with brine, dried over MgSO_4_, filtered, and concentrated under reduced pressure. The crude product was purified by column chromatography (CH_2_Cl_2_/Hexane, 2:1, *v*/*v*) to afford 24 mg (37%) of target compound **15** as a dark red solid.

### 4.15. 4,7-Di(9H-carbazol-9-yl)[1,2,5]thiadiazolo[3,4-d]pyridazine *(**16**)*

2,3-Dichloro-5,6-dicyano-1,4-benzoquinone (74 mg, 0.32 mmol) was added to a solution of amine **12d** (60 mg, 0.13 mmol) in toluene (12 mL). The mixture was refluxed for 7 h, diluted to with EtOAc (30 mL), washed with aq. NaHSO_3_, Na_2_CO_3_, water, and brine, dried over MgSO_4_, and concentrated under reduced pressure. The crude product was purified by column chromatography (CH_2_Cl_2_/Hexane, 2:1, *v*/*v*) to afford 46 mg (75%) of target compound **16** as a dark red solid, R_f_ = 0.4 (CH_2_Cl_2_). Mp > 260 °C. IR *ν*_max_ (KBr, cm^−1^): 3044, 2921, 2851, 1599, 1490, 1479, 1446, 1332, 1264, 1223, 1151, 865, 739, 717, 511. ^1^H-NMR (300 MHz, CDCl_3_): δ 7.43–7.54 (m, 8H), 7.86 (d, *J* = 7.8 Hz, 4H), 8.20 (d, *J* = 7.2 Hz, 4H). ^13^C-NMR (75 MHz, CDCl_3_): δ 113.1, 120.7, 122.9, 125.8, 126.8, 140.0, 148.4, 148.5. HRMS (ESI-TOF), *m*/*z*: calcd for C_28_H_17_N_6_S [M + H]^+^, 469.1230, found, 469.1247. MS (EI, 70 eV), *m*/*z* (*I*, %): 470 ([M + 2]^+^, 10), 469 ([M + 1]^+^, 30), 468 (M^+^, 100), 387 ([M − 1]^+^, 12), 302 (80), 168 (45), 148 (28).

## 5. Conclusions

The convenient and safe synthesis of dimethyl 1,2,5-thiadiazole-3,4-dicarboxylate from commercially available 2,3-diaminomaleodinitrile was proposed; this clears the way for large-scale preparation of 4,7-dibromo[1,2,5]thiadiazolo[3,4-*d*]pyridazine. The conditions for selective S_N_Ar substitution of one and two bromine atoms in 4,7-dibromo[1,2,5]thiadiazolo[3,4-*d*]pyridazine by oxygen and nitrogen nucleophiles were found. In the case of thiols, the reaction cannot be stopped at the mono-thiols stage, and bis-thiols were formed in high yields regardless of the reaction conditions. Buchwald-Hartwig or Ullmann reactions were successfully employed for the incorporation of a weak nitrogen substituent, e.g., carbazole, to the [1,2,5]thiadiazolo[3,4-*d*]pyridazine system. Very rare symmetrical S…η^2^-(N=N) interactions were discovered by X-ray analysis of 4,7-bis(hexylthio)-[1,2,5]thiadiazolo[3,4-d]pyridazine; the stability of these interactions may be used for the construction of liquid crystalline materials.

## Figures and Tables

**Table 1:**
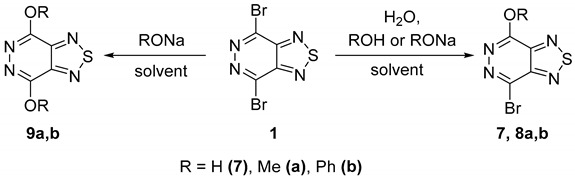
Reaction of 4,7-dibromo[1,2,5]thiadiazolo[3,4-*d*]pyridazine **1** with *O*-nucleophiles.

Entry	Reagent (eqv.)	Solvent	Temp. (°C)	Time (h)	Yield (%)
1	H_2_O (excess)	H_2_O	25	30	**7** (75)
2	H_2_O (1)	CHCl_3_	25	30	**7** (80)
3	H_2_O (2)	CHCl_3_	25	30	**7** (82)
4	H_2_O (1)	CHCl_3_	60	20	**7** (75)
5	H_2_O (2)	CHCl_3_	60	20	**7** (77)
6	MeOH (excess)	CHCl_3_	25	16	**7** (78)
7	MeONa (1)	MeOH	25	6	**8a** (80)
8	MeONa (2)	MeOH	25	24	**9a** (82)
9	MeONa (2)	MeOH	64	6	**9a** (70)
10	PhOH (1)	THF	25	8	- *
11	PhOH (1)	DMF	25	8	- *
12	PhONa (1)	THF	25	8	**8b** (80)
13	PhONa (2)	THF	25	8	**8b** (78)
14	PhONa (2)	THF	60	6	**8b** (74)
15	PhONa (1)	DMF	25	8	**8b** (70)
16	PhONa (2)	DMF	25	8	**8b** (73)
17	PhONa (2)	DMF	90	6	**9b** (69)

* Starting dibromo derivative **1** was isolated in nearly quantitative yield.

**Table 2:**
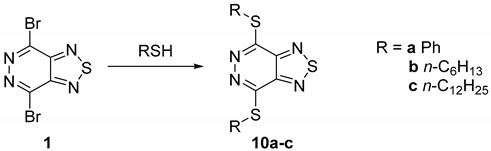
Reaction of 4,7-dibromo[1,2,5]thiadiazolo[3,4-*d*]pyridazine **1** with *S*-nucleophiles.

Entry	RSH (equiv.)	Solvent	Base (equiv.)	Time (h)	Yield (%)
1	**a** (1)	CHCl_3_	-	6	**10a** (45) *
2	**a** (2)	CHCl_3_	-	7	**10a** (78)
3	**a** (2)	THF	-	4	**10a**(80)
4	**a** (2)	MeCN	-	4	**10a** (76)
5	**a** (2)	DMF	-	1	**10a** (77)
6	**a** (2)	THF	NaH (2)	3	**10a** (85)
7	**b** (2)	THF	NaH (2)	3	**10b** (90)
8	**c** (2)	THF	NaH (2)	4	**10c** (88)

* The starting dibromo derivative **1** was isolated in 52% yield.

**Table 3:**
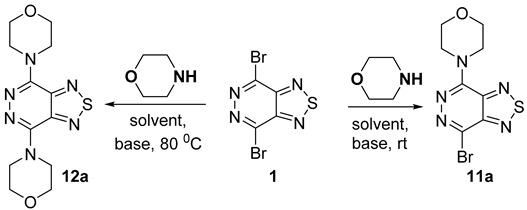
Nucleophilic substitution of 4,7-dibromo[1,2,5]thiadiazolo[3,4-*d*]pyridazine **1** by morpholine.

Entry	Morpholine (eqv.)	Solvent	Base (eqv.)	Temp. (°C)	Time (h)	Yields (%)	
						11a	12a
1	2	MeOH	-	25	6	79	0
2	4	MeOH	-	25	12	78	0
3	2	CH_2_Cl_2_	-	25	4	82	0
4	4	CH_2_Cl_2_	-	25	10	83	0
**5**	**1**	**CH_2_Cl_2_**	**Et_3_N (1)**	**25**	**4**	**86**	**0**
6	2	MeCN	-	25	3	80	0
7	4	MeCN	-	25	6	82	0
8	1	MeCN	Et_3_N (1)	25	3	81	0
9	2	DMF	-	25	0.5	84	0
10	4	DMF	-	25	2	84	0
11	1	DMF	Et_3_N (1)	25	0.5	82	0
12	2	CHCl_3_	Et_3_N (2)	61	50	0	70
**13**	**2**	**MeCN**	**Et_3_N (2)**	**80**	**30**	**0**	**87**
14	2	DMF	Et_3_N (2)	80	20	0	82

**Table 4:**
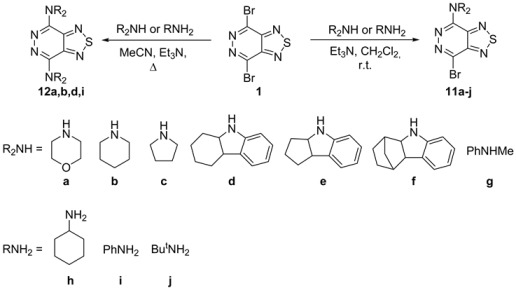
Reaction of 4,7-dibromo[1,2,5]thiadiazolo[3,4-*d*]pyridazine (**1**) with amines.

Entry	Amine (eqv.)	Solvent	Base (eqv.)	Temp. (°C)	Time (h)	Yields (%)
1	**a** (1)	CH_2_Cl_2_	Et_3_N (1)	25	4	**11a** (86)
2	**b** (1)	CH_2_Cl_2_	Et_3_N (1)	25	4	**11b** (82)
3	**c** (1)	CH_2_Cl_2_	Et_3_N (1)	25	4	**11c** (81)
4	**d** (1)	CH_2_Cl_2_	Et_3_N (1)	25	4	**11d** (85)
5	**e** (1)	CH_2_Cl_2_	Et_3_N (1)	25	4	**11e** (82)
6	**f** (1)	CH_2_Cl_2_	Et_3_N (1)	25	4	**11f** (79)
7	**g** (1)	CH_2_Cl_2_	Et_3_N (1)	25	4	**11g** (80)
8	**h** (1)	CH_2_Cl_2_	Et_3_N (1)	25	4	**11h** (85)
9	**i** (1)	CH_2_Cl_2_	Et_3_N (1)	25	4	**11i** (80)
10	**j**(1)	CH_2_Cl_2_	Et_3_N (1)	25	4	**11j** (75)
11	**a** (2)	MeCN	Et_3_N (2)	80	30	**12a** (87)
12	**b** (2)	MeCN	Et_3_N (2)	80	30	**12b** (85)
13	**d** (2)	MeCN	Et_3_N (2)	80	10	**12d** (90)
14	**i** (2)	MeCN	Et_3_N (2)	80	30	**12i** (78)

**Table 5:**
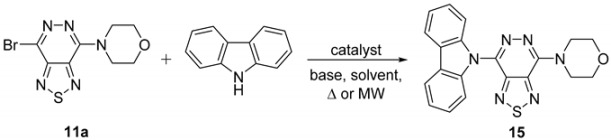
Buchwald-Hartwig and Ullmann coupling of 4-(7-bromo[1,2,5]thiadiazolo[3,4-*d*]pyridazin-4-yl)morpholine **11a** with carbazole.

Entry	Catalyst (mol%)	Ligand (mol%)	Solvent	Temp. (°C)	Time (h)	15, Yield (%)
1	Pd_2_(dba)_3_ (10)	-	toluene	111	12	decomp.
2	Pd(OAc)_2_ (10)	-	toluene	111 *	0.25	decomp.
3	Pd_2_(dba)_3_ (10)	XPhos (5)	toluene	111	12	5
4	Pd(OAc)_2_ (10)	XPhos (5)	toluene	111	12	8
5	Pd_2_(dba)_3_ (10)	XPhos (5)	Toluene	111 *	0.5	20
6	Pd(OAc)_2_ (10)	XPhos (5)	toluene	111 *	0.5	40
7	CuI (10)	DMEDA (5)	dioxane/H_2_O 3:1	100	5	30
8	CuI (10)	DMEDA (5)	dioxane/H_2_O 3:1	100 *	0.17	37

* In microwave oven at MW = 300 W.

**Table 6:** Crystallographic data for **10b** and **16**.

	10b	16
Chemical formula	C_16_H_26_N_4_S_3_	C_28_H_16_N_6_S, 0.5 (CH_2_Cl_2_)
Formula weight (g mol^−1^)	370.59	510.99
Temperature (K)	120	120
Crystal system	Monoclinic	Triclinic
Space group	C2/c	P-1
a (Å)	22.221(5)	9.646(2)
b (Å)	7.5589(16)	12.785(3)
c (Å)	35.127(7)	18.575(4)
α (°)	90	83.824(5)
β (°)	104.940(11)	89.837(5)
γ (°)	90	89.640(5)
V (Å^3^)	5701(2)	2277.3(8)
Z/Z’	12/1.5	4/2
d_calc_ (g cm^–3^)	1.295	1.490
μ(MoKα)	3.95	2.93
2Θmax	56	58
Reflns. Collected/unique	31,479/6870	28,461/12,099
Observed reflns [I > 2σ(I)]	3287	6756
R_int_(I)	0.0843	0.0957
R_1_(F^2^)	0.0737	0.0689
wR_2_	0.2052	0.1932
GOF	1.022	1.043
Δρ_min_/Δρ_max_	−0.416/0.939	−0.812/1.049

## Supplementary Materials

The following are available online. Characterization data including ^1^H and ^13^C-NMR spectra for novel compounds and X-ray crystallographic figures.

## References

[1] Parker T.C., Dan Patel D.G., Moudgil K., Barlow S., Risko C., Brédas J.-L., Reynolds J.R., Marder S.R. (2015). Heteroannulated acceptors based on benzothiadiazole. Mater. Horiz..

[2] Knyazeva E.A., Rakitin O.A. (2016). Influence of structural factors on the photovoltaic properties of dye-sensitized solar cells. Russ. Chem. Rev..

[3] Zhang X., Grätzel M., Hua J. (2016). Donor design and modification strategies of metal-free sensitizers for highly-efficient n-type dye-sensitized solar cells. Front. Optoelectron..

[4] Takimiya K., Osaka I., Nakano M. (2014). π-Building Blocks for Organic Electronics: Revaluation of “Inductive” and “Resonance” Effects of π-Electron Deficient Units. Chem. Mater..

[5] Wu Y., Zhu W. (2013). Organic sensitizers from D–p–A to D–A–p–A: Effect of the internal electron-withdrawing units on molecular absorption, energy levels and photovoltaic performances. Chem. Soc. Rev..

[6] Duan C., Huang F., Cao Y. (2012). Recent development of push–pull conjugated polymers for bulk-heterojunction photovoltaics: Rational design and fine tailoring of molecular structures. J. Mater. Chem..

[7] Pron A., Leclerc M. (2013). Imide/amide based π-conjugated polymers for organic electronics. Prog. Polym. Sci..

[8] Lee C.P., Li C.T., Ho K.C. (2017). Use of organic materials in dye-sensitized solar cells. Mater. Today.

[9] Zhao Y., Guo Y., Liu Y. (2013). 25th Anniversary Article: Recent Advances in n-Type and Ambipolar Organic Field-Effect Transistors. Adv. Mater..

[10] Guo X., Facchetti A., Marks T.J. (2014). Imide- and Amide-Functionalized Polymer Semiconductors. Chem. Rev..

[11] Zhang Y., Zou J., Cheuh C.C., Yip H.L., Jen A.K.Y. (2012). Significant Improved Performance of Photovoltaic Cells Made from a Partially Fluorinated Cyclopentadithiophene/Benzothiadiazole Conjugated Polymer. Macromolecules.

[12] Stuart A.C., Tumbleston J.R., Zhou H., Li W., Liu S., Ade H., You W. (2013). Fluorine Substituents Reduce Charge Recombination and Drive Structure and Morphology Development in Polymer Solar Cells. J. Am. Chem. Soc..

[13] Van Mullekom H.A.M., Vekemans J., Havinga E.E., Meijer E.W. (2001). Developments in the chemistry and band gap engineering of donor–acceptor substituted conjugated polymers. Mater. Sci. Eng. R.

[14] Roncali J. (2007). Molecular Engineering of the Band Gap of π-Conjugated Systems: Facing Technological Applications. Macromol. Rapid Commun..

[15] Velusamy M., Thomas K.R.J., Lin J.T., Hsu Y.C., Ho K.C. (2005). Organic Dyes Incorporating Low-Band-Gap Chromophores for Dye-Sensitized Solar Cells. Org. Lett..

[16] Cui Y., Wu Y.Z., Lu X.F., Zhang X., Zhou G., Miapeh F.B., Zhu W.H., Wang Z.S. (2011). Incorporating Benzotriazole Moiety to Construct D-A-π-A Organic Sensitizers for Solar Cells: Significant Enhancement of Open-Circuit Photovoltage with Long Alkyl Group. Chem. Mater..

[17] Pei K., Wu Y.Z., Wu W.J., Zhang Q., Chen B.Q., Tian H., Zhu W.H. (2012). Constructing Organic D–A–p-A-Featured Sensitizers with a Quinoxaline Unit for High-Efficiency Solar Cells: The Effect of an Auxiliary Acceptor on the Absorption and the Energy Level Alignment. Chem. Eur. J..

[18] Li W.Q., Wu Y.Z., Zhang Q., Tian H., Zhu W.H. (2012). D-A-π-A Featured Sensitizers Bearing Phthalimide and Benzotriazole as Auxiliary Acceptor: Effect on Absorption and Charge Recombination Dynamics in Dye-Sensitized Solar Cells. ACS Appl. Mater. Interfaces.

[19] Qu S.Y., Wu W.J., Hua J.L., Kong C.Y., Long T., Tian H. (2010). New Diketopyrrolopyrrole (DPP) Dyes for Efficient Dye-Sensitized Solar Cells. J. Phys. Chem. C.

[20] Lu X.F., Zhou G., Wang H., Feng Q.Y., Wang Z.S. (2012). Near infrared thieno[3,4-b]pyrazine sensitizers for efficient quasi-solid-state dye-sensitized solar cells. Phys. Chem. Chem. Phys..

[21] Liu J., Wang K., Xu F., Tang Z., Zheng W., Zhang J., Li C., Yu T., You X. (2011). Synthesis and photovoltaic performances of donor–π–acceptor dyes utilizing 1,3,5-triazine as π spacers. Tetrahedron Lett..

[22] Chmovzh T.N., Knyazeva E.A., Mikhalchenko L.V., Golovanov I.S., Amelichev S.A., Rakitin O.A. (2018). Synthesis of 4,7-dibromo derivative of ultrahigh electron-deficient [1,2,5]thiadiazolo[3,4-*d*]pyridazine heterocycle and its cross-coupling reactions, Eur. J. Org. Chem. Eur. J. Org. Chem..

[23] Warren J.D., Lee V.J., Angier R.B. (1979). Synthesis of 5,8-dihydroxynaphtho[2,3-c][1,2,5]thiadiazole-6,9-dione and 6,9-dihydroxybenzo[g]quinoxaline-5,10-dione. J. Heterocycl. Chem..

[24] Mataka S., Takahashi K., Yamada Y., Tashiro M. (1979). Sulfur nitride in organic chemistry. 6 Preparation of 3,4-disubstituted 1,2,5-thiadiazoles by the reaction of sulfur nitride with acetylenes. J. Heterocycl. Chem..

[25] Kolb H.C., Bennani Y.L., Sharpless K.B. (1993). Short and practical syntheses of (*R*)-(−)-carnitine and (*R*)-(−)-γ-amino-β-hydroxybutyric acid (GABOB). Tetrahedron Asymmetry.

[26] Biagi G., Giorgi I., Livi O., Manera C., Scartoni V., Betti L., Giannaccini G., Lucacchini A. (1999). 1,2,3-Triazolo[4,5-d]pyridazines: Part VI. New 1-substituted-4-amino derivatives and their affinity towards A1 and A2A adenosine receptors. Il Farm..

[27] Biagi G., Giorgi I., Livi O., Scartoni V., Velo S., Martini C., Senatore G., Barili P.L. (1995). 1,2,3-Triazole[4,5-d]pyridazines. 4. Preparation and adenosine receptor-binding of new 4 and/or 7 aminoderivatives. Il Farm..

[28] Miller-Moslin K., Peukert S., Jain R.K., McEwan M.A., Karki R., Llamas L., Yusuff N., He F., Li Y., Sun Y. (2009). 1-Amino-4-benzylphthalazines as Orally Bioavailable Smoothened Antagonists with Antitumor Activity. J. Med. Chem..

[29] Carbon J.A. (1958). The Preparation of Several 4-Substituted Imidazo[4,5-d]pyridazines as Possible Purine Antimetabolites. J. Am. Chem. Soc..

[30] Ni F., Wu Z., Zhu Z., Chen T., Wu K., Zhong C., An K., Wei D., Ma D., Yang C. (2017). Teaching an old acceptor new tricks: Rationally employing 2,1,3-benzothiadiazole as input to design a highly efficient red thermally activated delayed fluorescence emitter. J. Mater. Chem. C.

[31] Jung D., Thirupathaiah B., Lee E., Kwon G., Kim C., Seo S. (2016). Synthesis and Characterization of Benzothiadiazole Derivatives as Organic Semiconductors for Organic Thin-Film Transistors. J. Nanosci. Nanotechnol..

[32] Misra R., Gautam P. (2014). Tuning of the HOMO–LUMO gap of donor-substituted symmetrical and unsymmetrical benzothiadiazoles. Org. Biomol. Chem..

[33] Hostettler N., Wright I.A., Bozic-Weber B., Constable E.C., Housecroft C.E. (2015). Dye-sensitized solar cells with hole-stabilizing surfaces: “inorganic” *versus* “organic” strategies. RSC Adv..

[34] Ye Q., Chen S., Zhu D., Lu X., Lu Q. (2015). Preparation of aggregation-induced emission dots for long-term two-photon cell imaging. J. Mater. Chem. B.

[35] Liu Q., Liu Y., Wang Y., Ai L., Ouyang X., Han L., Ge Z. (2013). Anthradithiophene-benzothiadiazole-based small molecule donors for organic solar cells. New J. Chem..

[36] Misra R., Gautam P., Mobin S.M. (2013). Aryl-Substituted Unsymmetrical Benzothiadiazoles: Synthesis, Structure, and Properties. J. Org. Chem..

[37] Zhou Y.H., Wang Z.M., Chen M.D., Guo S.L., Zheng Y.X. (2013). Syntheses and photoluminescence properties of rhenium(I) complexes based on dipyrido[3,2-a:2′,3′-c]phenazine derivatives with carbazole moiety. J. Coord. Chem..

[38] Matta C.F., Boyd R.J. (2007). The Quantum Theory of Atoms in Molecules: From Solid State to DNA and Drug Design.

[39] Espinosa E., Molins E., Lecomte C. (1998). Hydrogen bond strengths revealed by topological analyses of experimentally observed electron densities. Chem. Phys. Lett..

[40] Ananyev I.V., Karnoukhova V.A., Dmitrienko A.O., Lyssenko K.A. (2017). Toward a Rigorous Definition of a Strength of Any Interaction Between Bader’s Atomic Basins. J. Phys. Chem. A.

[41] Sheldrick G.M. (2008). SADABS v2008/1, Bruker/Siemens Area Detector Absorption Correction Program.

[42] Sheldrick G.M. (2015). Crystal structure refinement with *SHELXL*. Acta Crystallogr. Sect. A..

[43] Duan X.G., Duan X.-L., Rees C.W., Yue T.Y. (1997). Reaction of trithiazyl trichloride with alkenes and alkynes. J. Chem. Soc. Perkin Trans. 1.

[44] Sekikawa I. (1960). Oxidation of 5-Methyl-2,1,3-benzothiadiazole with Potassium Permanganate. Bull. Chem. Soc. Japan.

