# Supplementary material for: Safe Synthesis of 4,7-Dibromo[1,2,5]thiadiazolo[3,4-d]pyridazine and Its SNAr Reactions
Source: Molecules. 2018 Oct 9;23(10):2576. doi: 10.3390/molecules23102576 (PMC6222427; doi:10.3390/molecules23102576)
Supplement: Supplementary file 1 [file molecules-23-02576-s001.pdf]

Supporting Information

Safe Synthesis of 4,7-Dibromo[1,2,5]thiadiazolo[3,4-*d*]pyridazine and its S_NAr Reactions

Timofey N. Chmovzh [1], Ekaterina A. Knyazeva [1,2], Konstantin A. Lyssenko [3], Vadim V. Popov [2], and Oleg A. Rakitin [1,2,]*

[1] N. D. Zelinsky Institute of Organic Chemistry, Russian Academy of Sciences, 119991 Moscow, Russian Federation; orakitin@ioc.ac.ru (O.A.R.); tim1661@yandex.ru (T.N.C.); katerina_knyazev@ioc.ac.ru (E.A.K.)

[2] Nanotechnology Education and Research Center, South Ural State University, 454080 Chelyabinsk, Russia; rakitinoa@susu.ru (O.A.R.); popov.ioc@gmail.com (V.V.P.)

[5] A. N. Nesmeyanov Institute of Organoelement Compounds, Russian Academy of Sciences, 119991 Moscow, Russia; kostya@ineos.ac.ru

Corresponding author. E-mail: orakitin@ioc.ac.ru

Table of Contents

1. **^{1}H and ^{13}C-NMR spectra** **S2**
2. **X-ray crystallography** **S29**

1. ^{1}H and ^{13}C-NMR spectra

4,7-Dibromo[1,2,5]thiadiazolo[3,4-*d*]pyridazine (1)

^{13}C-NMR(75 MHz)

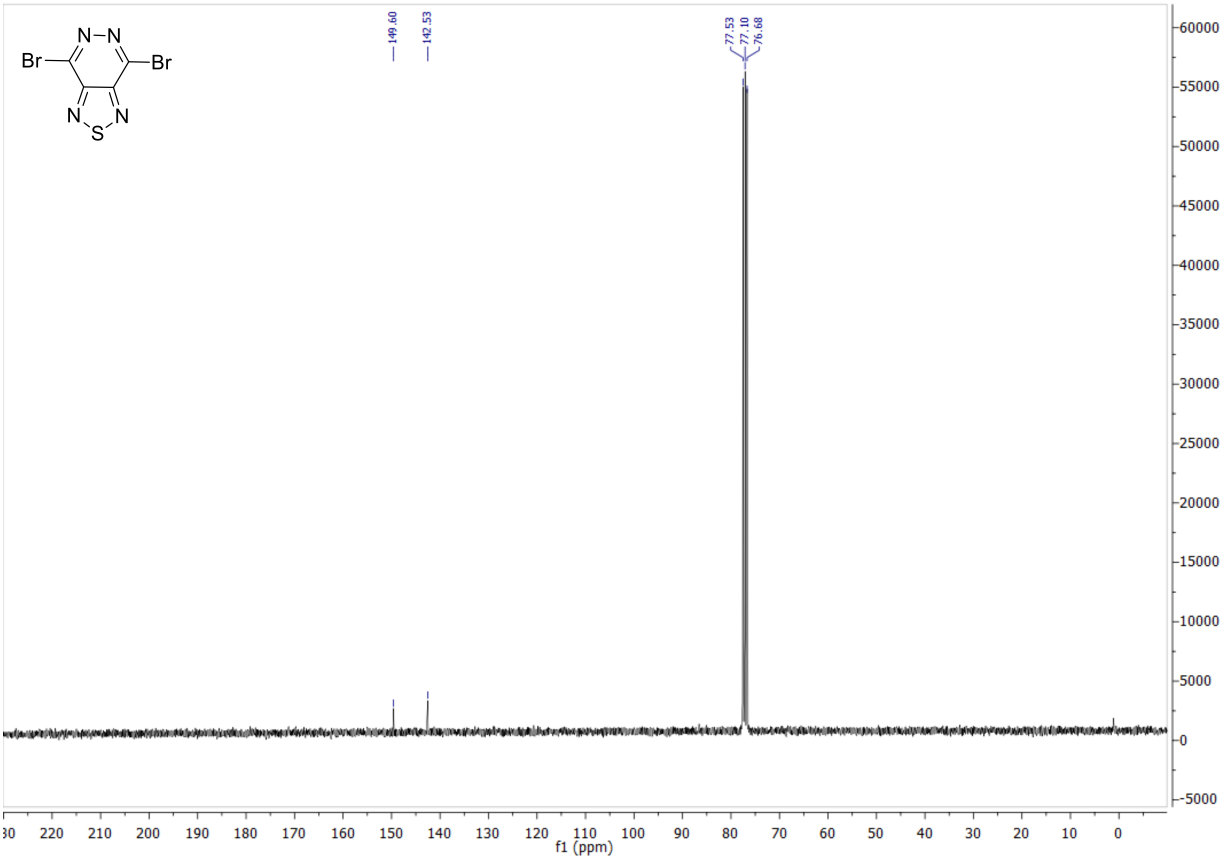

7-Bromo-[1,2,5]thiadiazolo[3,4]pyridazin-4-ol (7)

^{1}H-NMR(300 MHz)

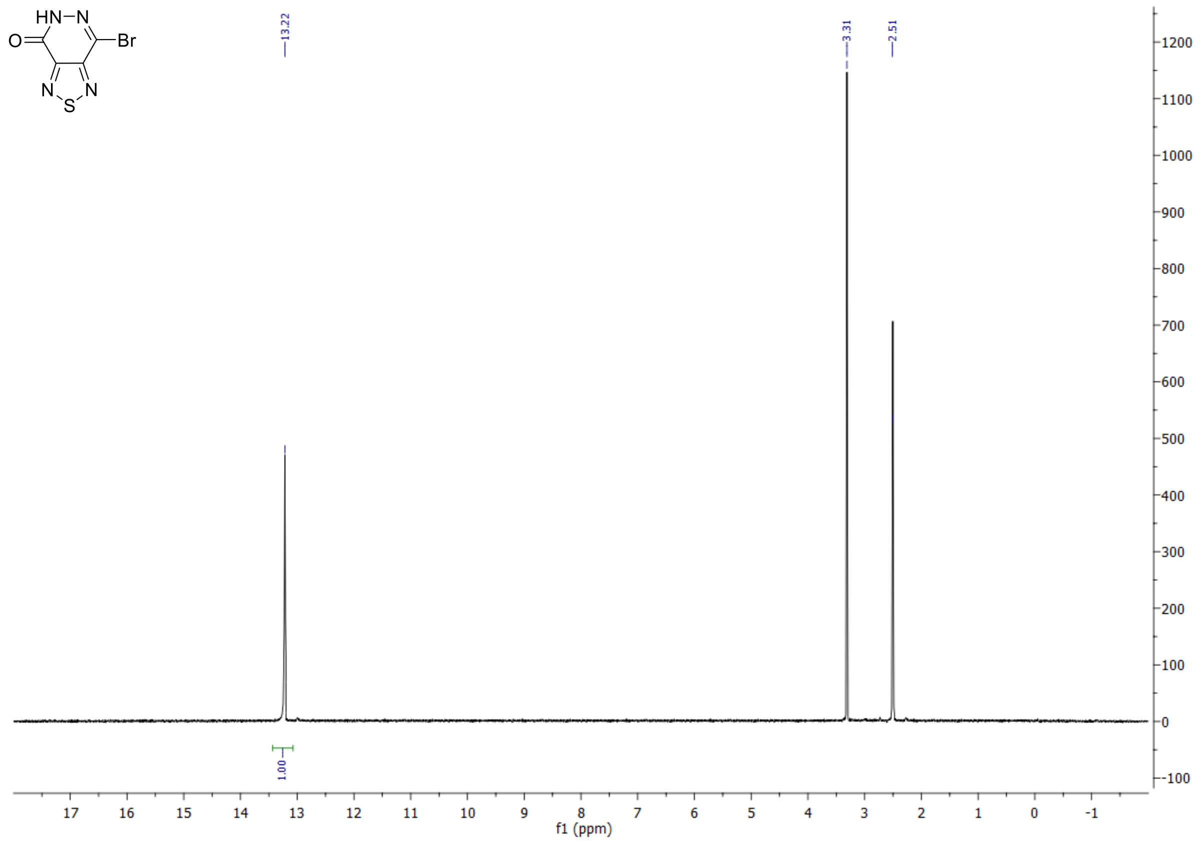

^{13}C-NMR(75 MHz)

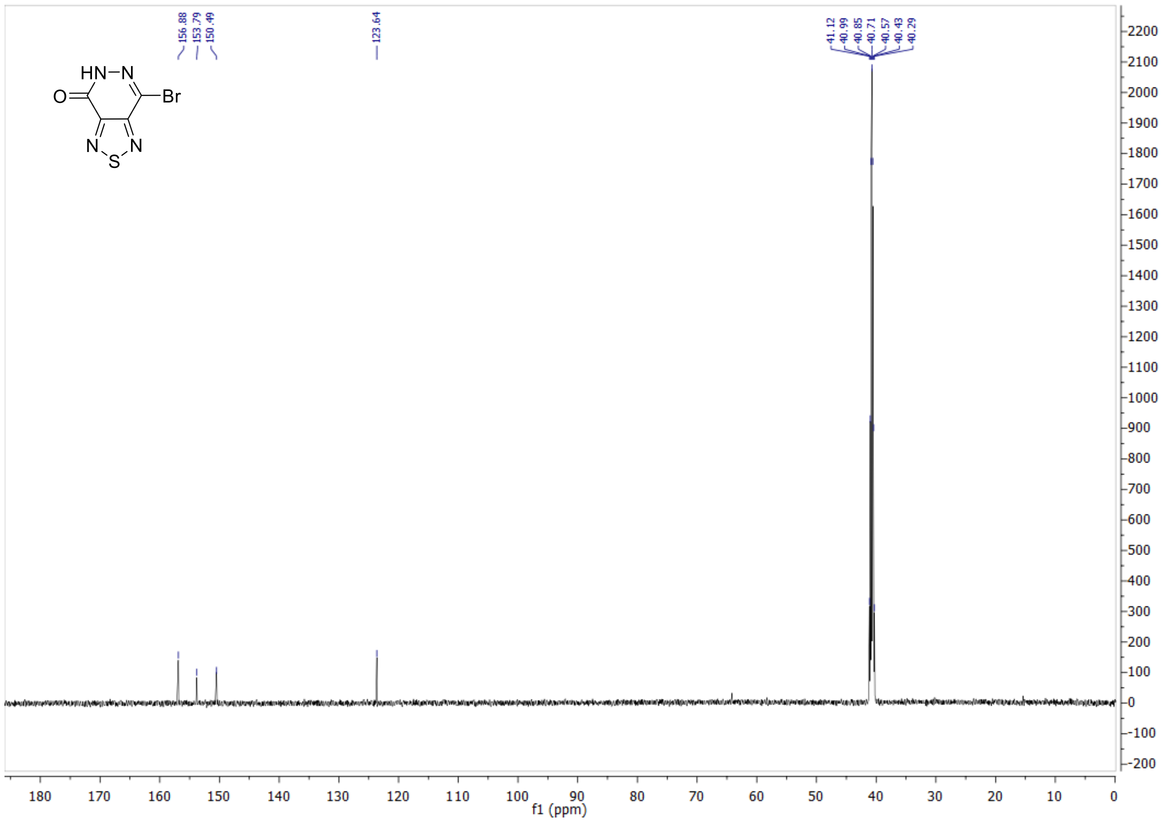

4-Bromo-7-methoxy-[1,2,5]thiadiazolo[3,4-d]pyridazine (8a)

^{1}H-NMR(300 MHz)

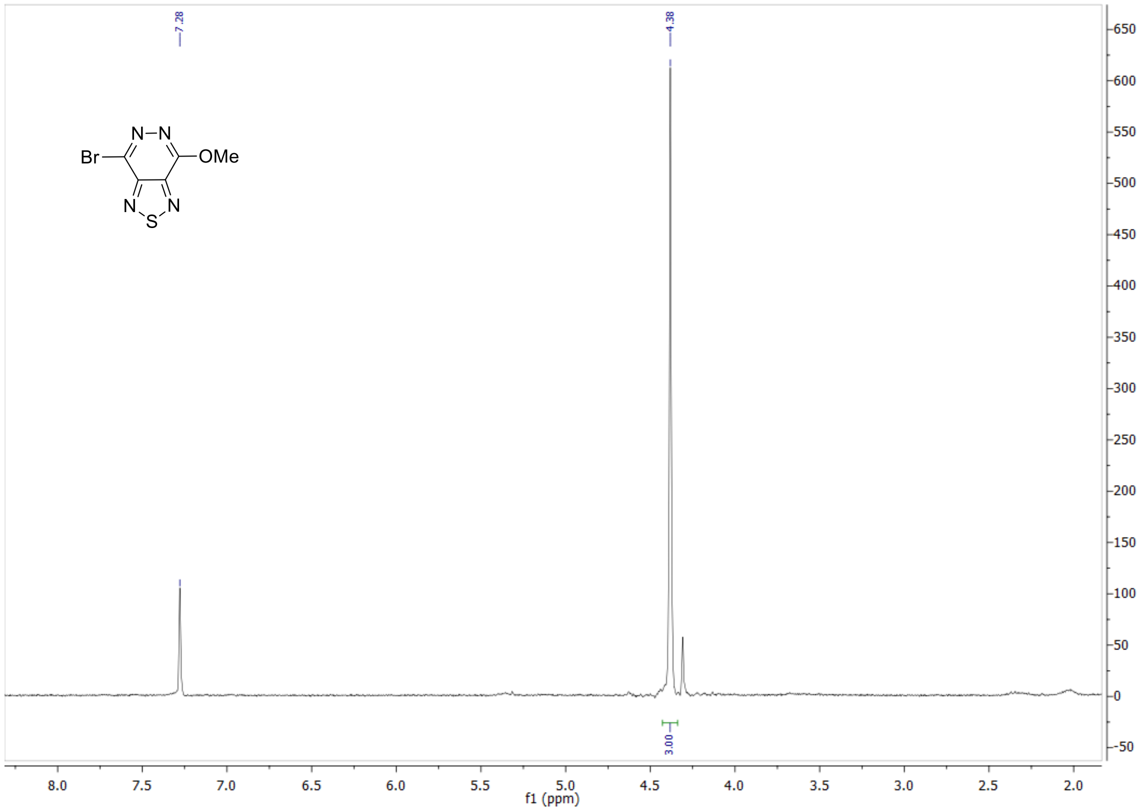

^{13}C-NMR(75 MHz)

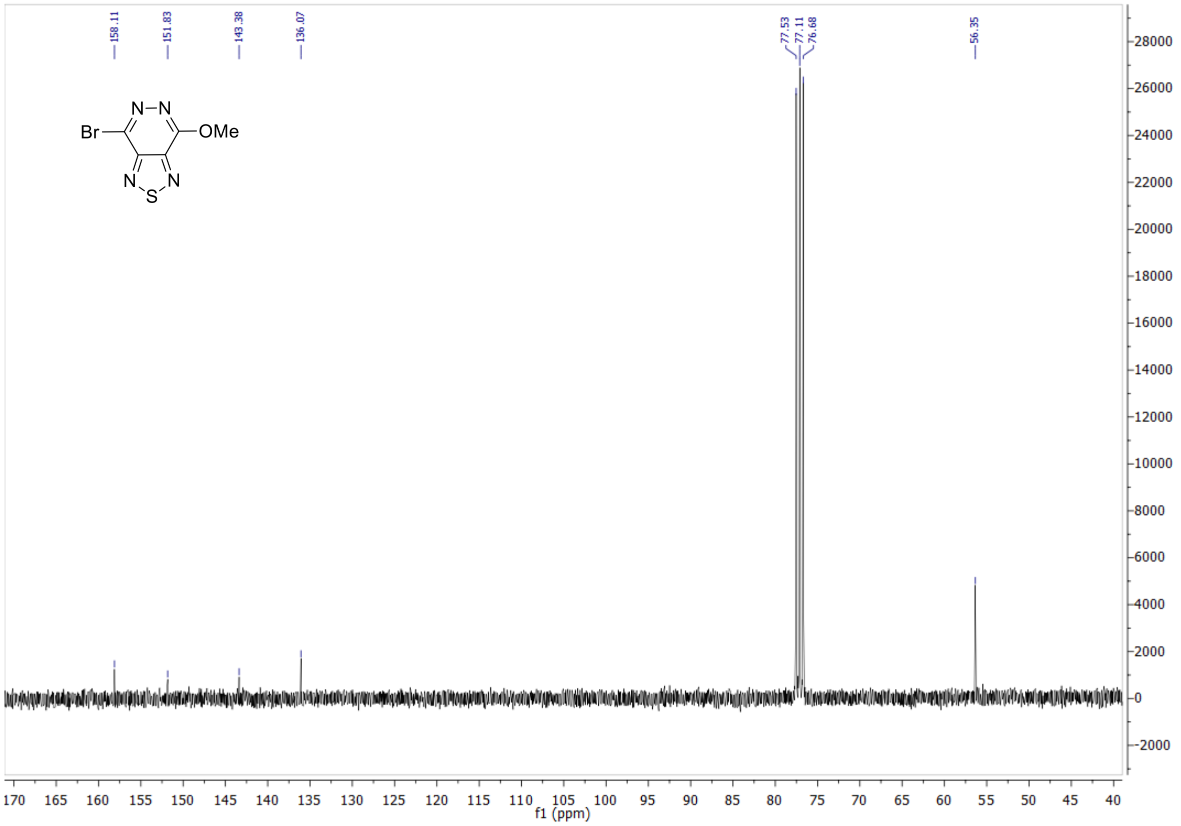

4-Bromo-7-phenoxy-[1,2,5]thiadiazolo[3,4-d]pyridazine (8b)

^{1}H-NMR(300 MHz)

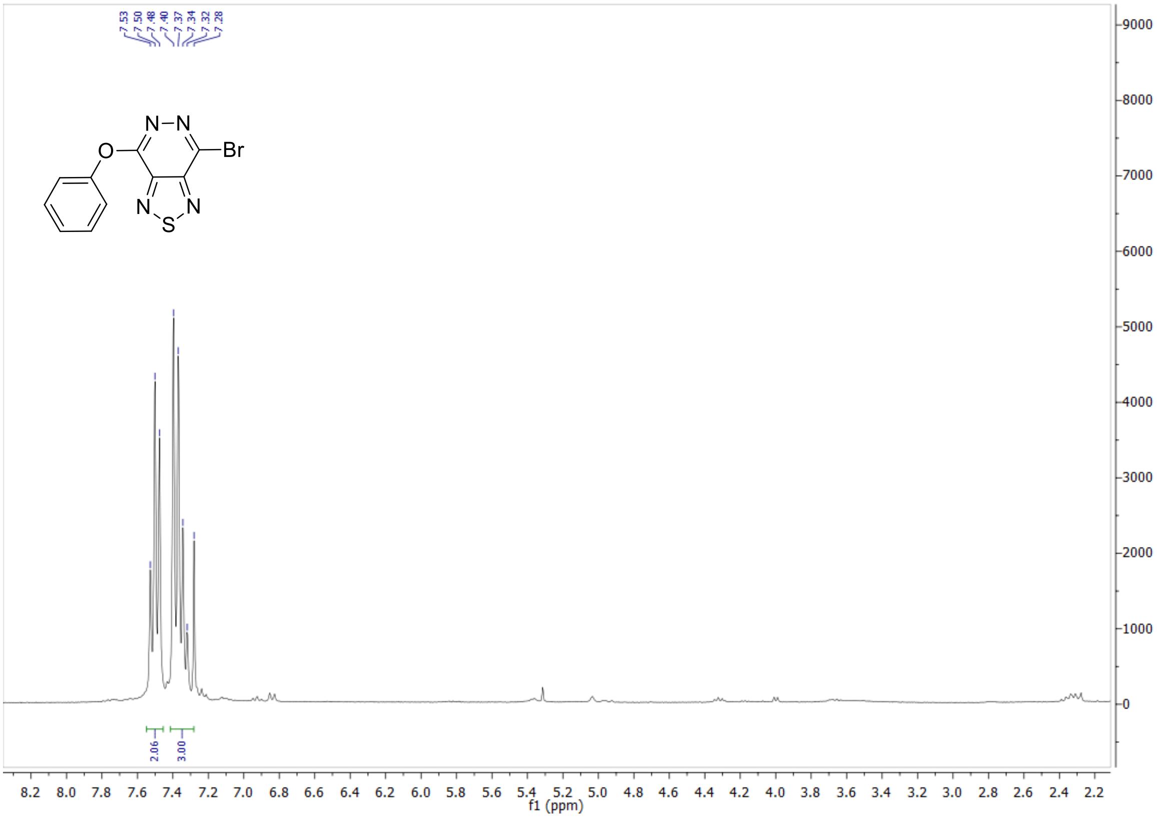

^{13}C-NMR(75 MHz)

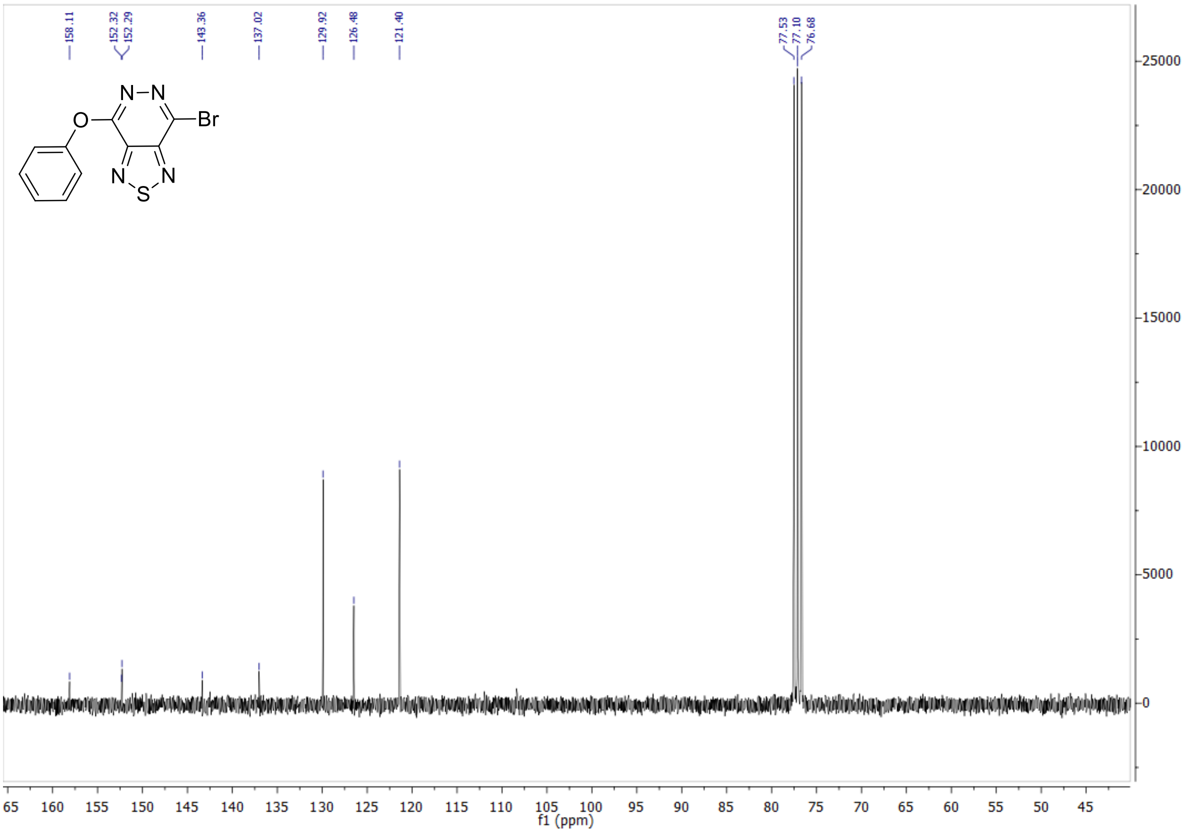

4,7-Dimethoxy-[1,2,5]thiadiazolo[3,4-d]pyridazine (9a)

^{1}H-NMR(300 MHz)

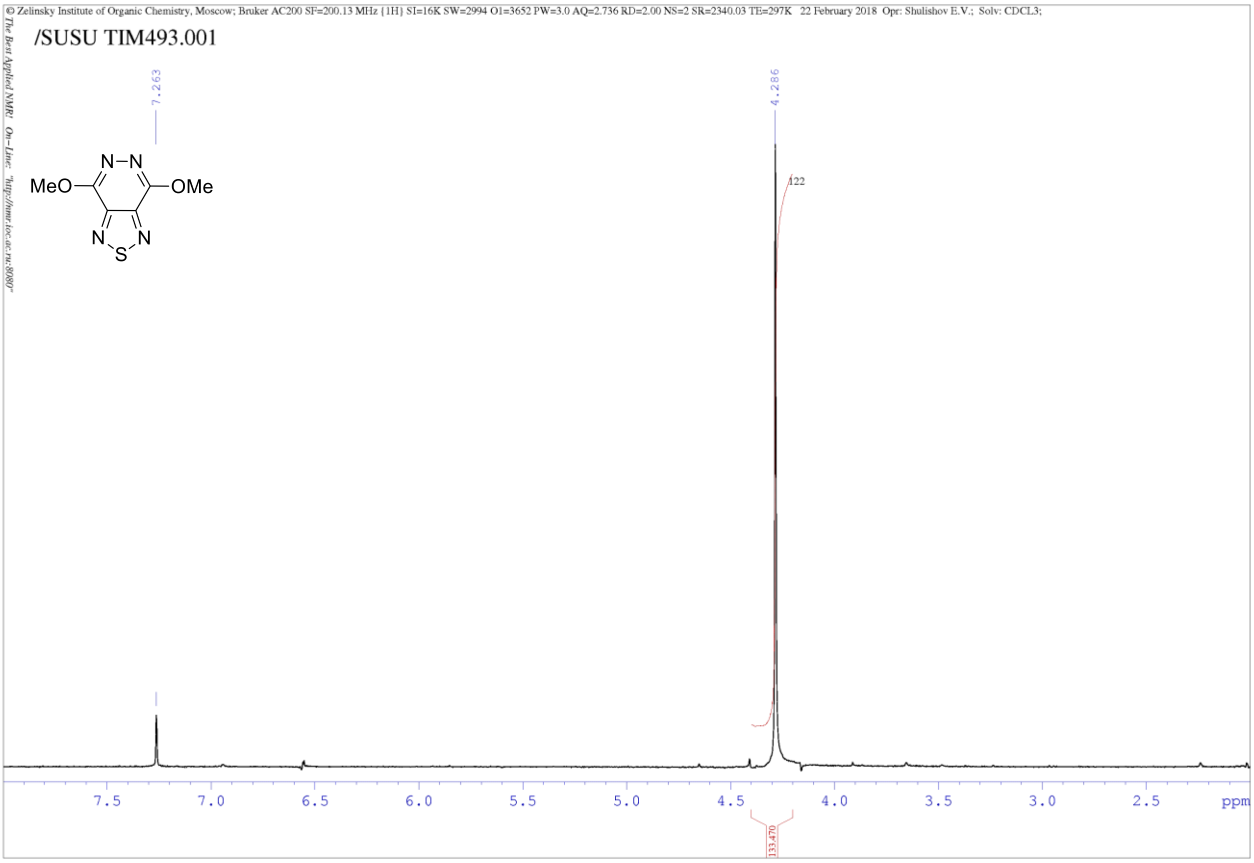

^{13}C-NMR(75 MHz)

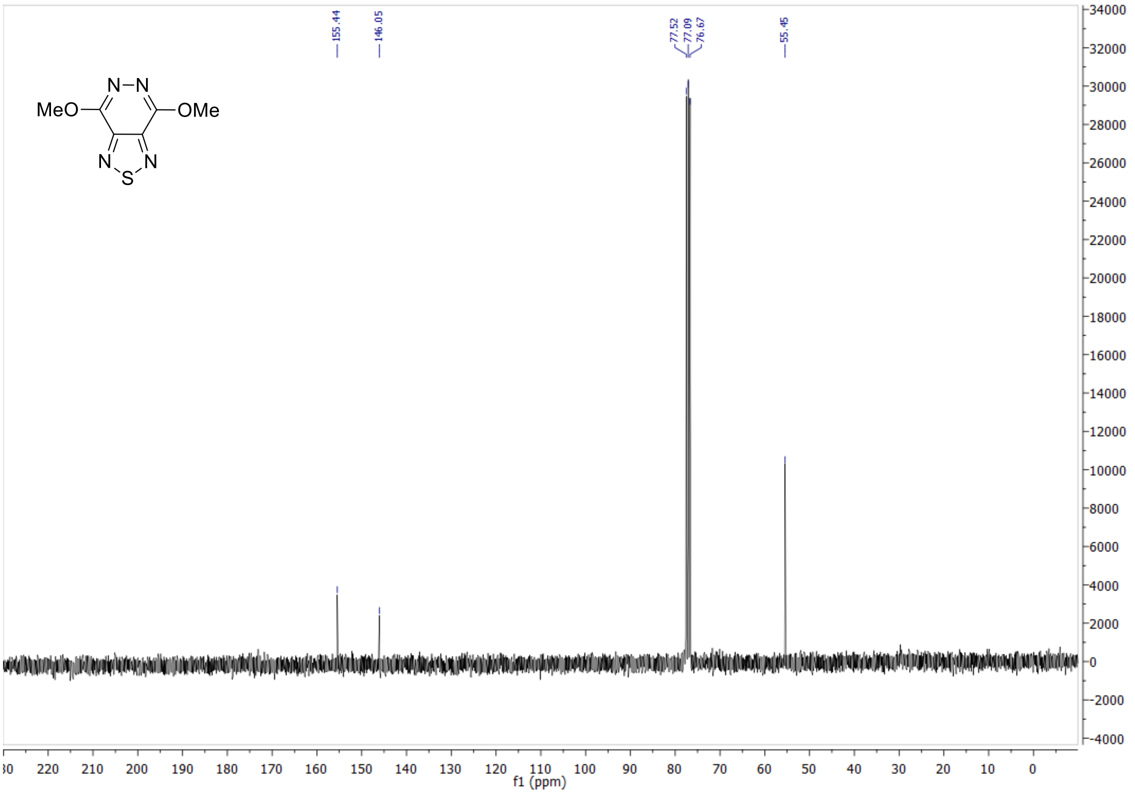

4,7-Diphenoxy-[1,2,5]thiadiazolo[3,4-d]pyridazine (9b)

^{1}H-NMR(300 MHz)

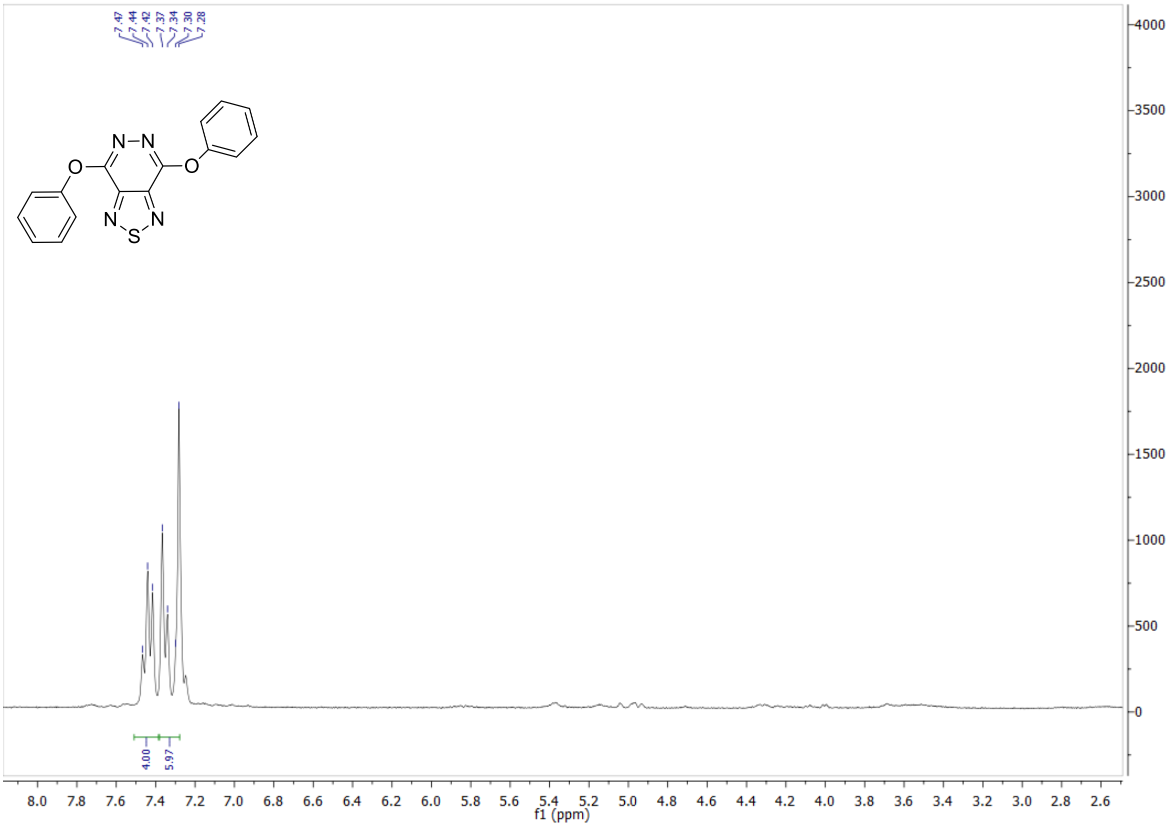

^{13}C-NMR(75 MHz)

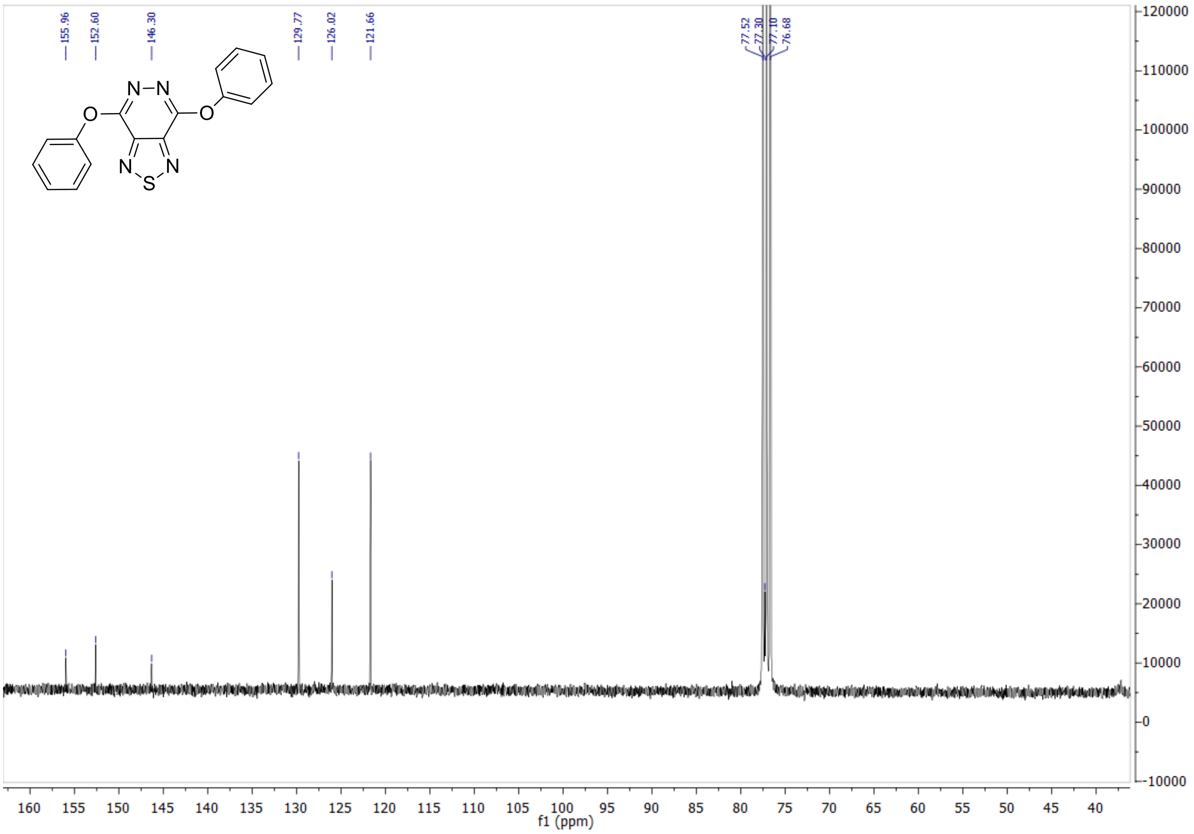

4,7-Bis(phenylthio)-[1,2,5]thiadiazolo[3,4-d]pyridazine (10a)

^{1}H-NMR(300 MHz)

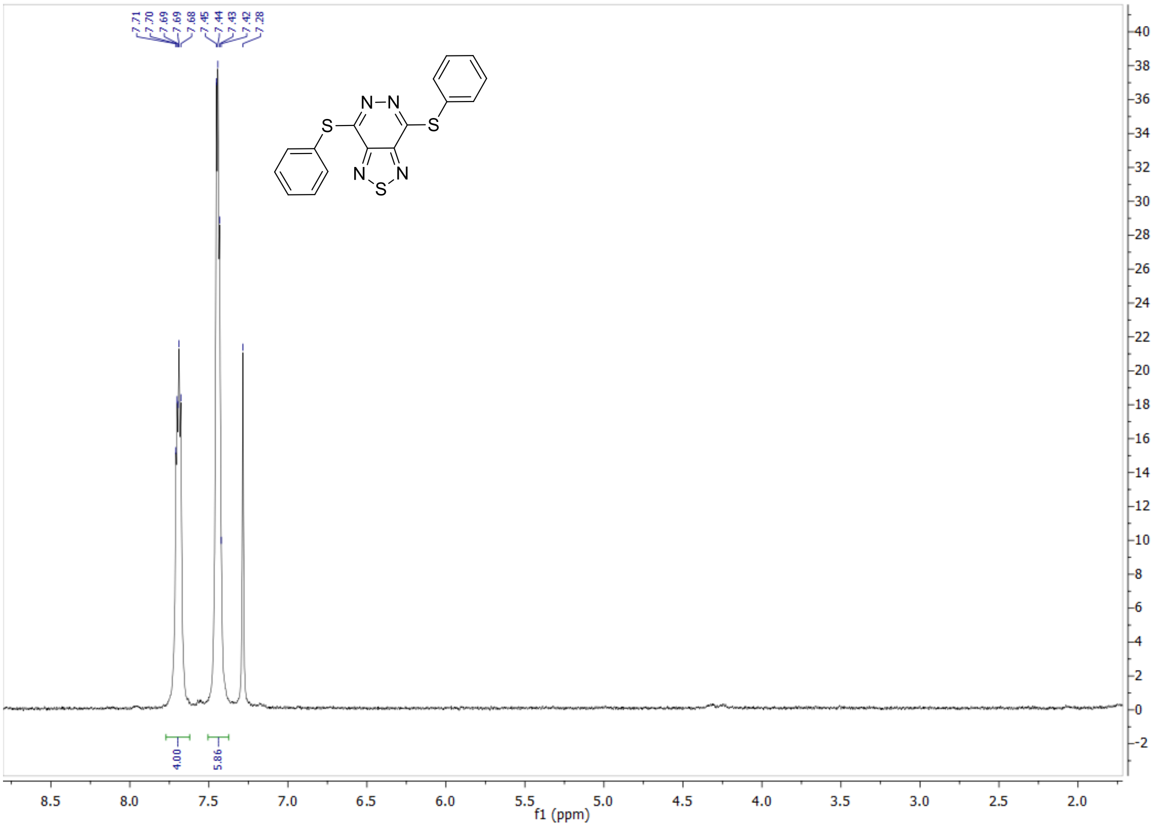

^{13}C-NMR(75 MHz)

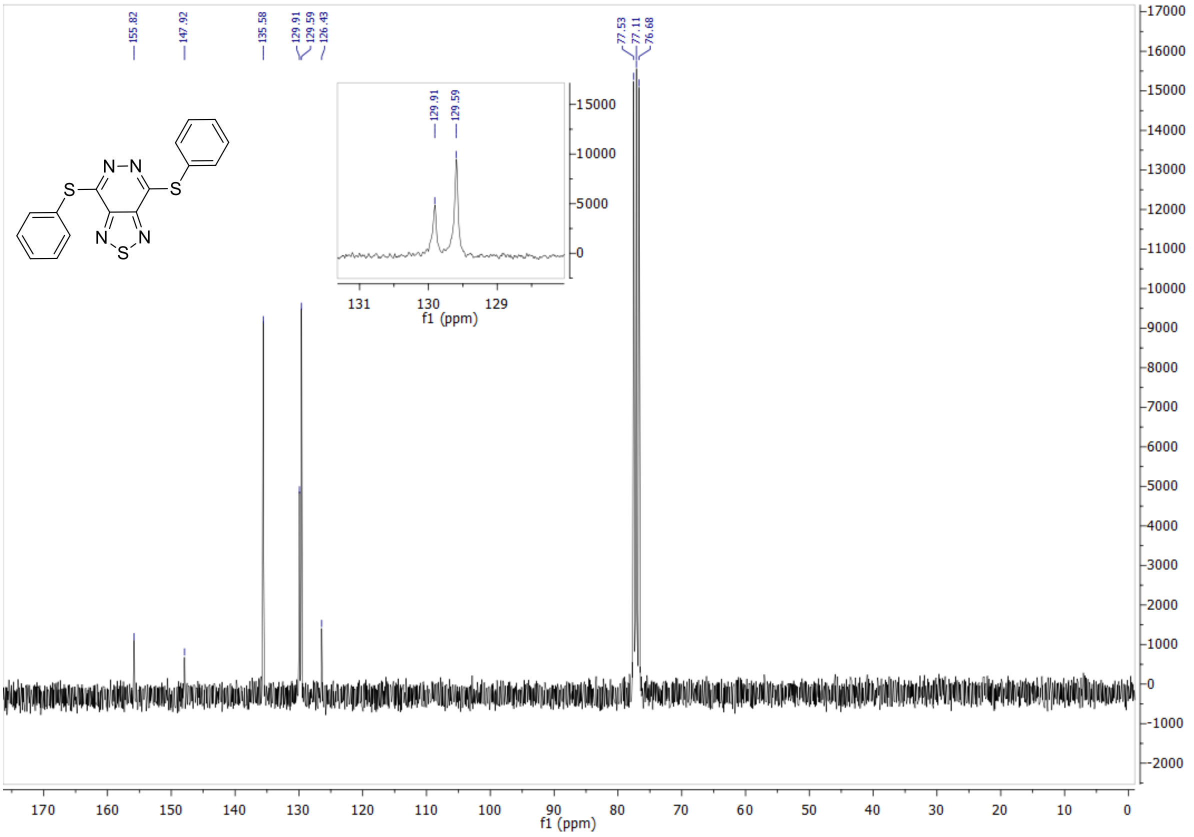

4,7-Bis(hexylthio)-[1,2,5]thiadiazolo[3,4-d]pyridazine (10b)

^{1}H-NMR(300 MHz)

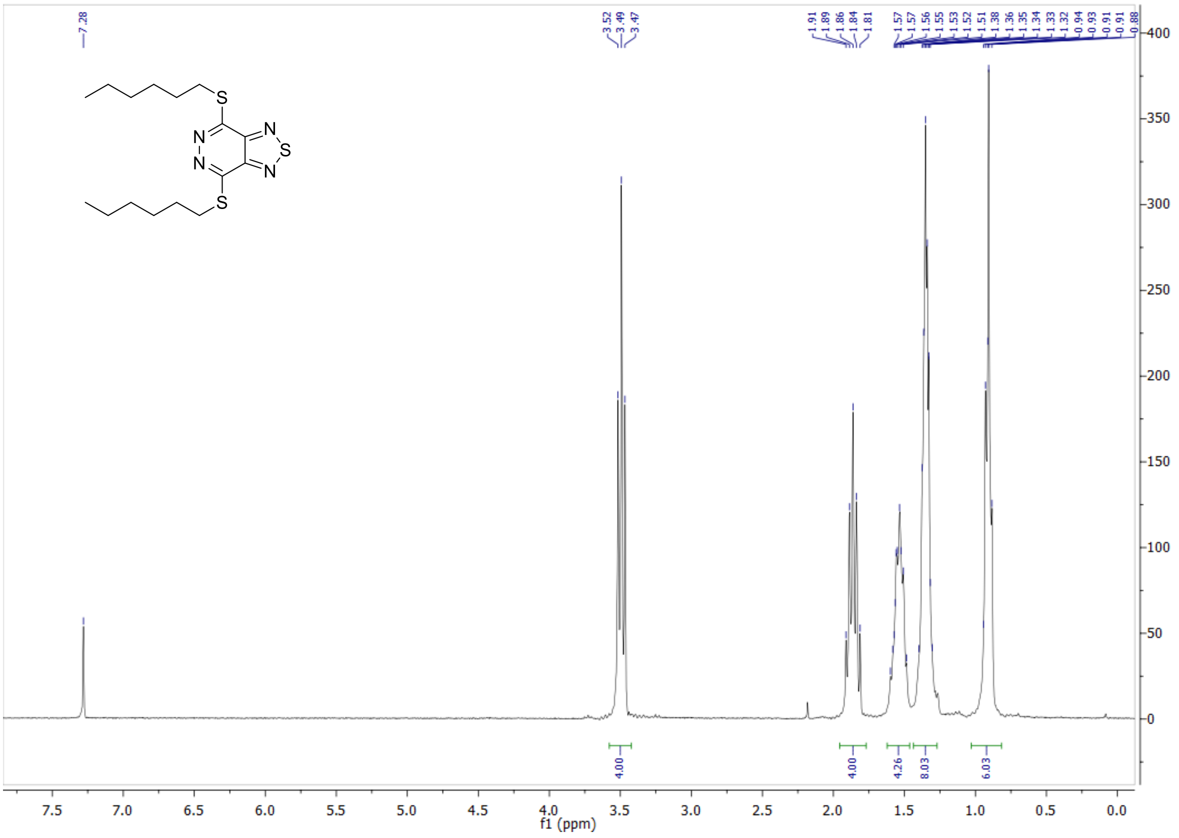

^{13}C-NMR(75 MHz)

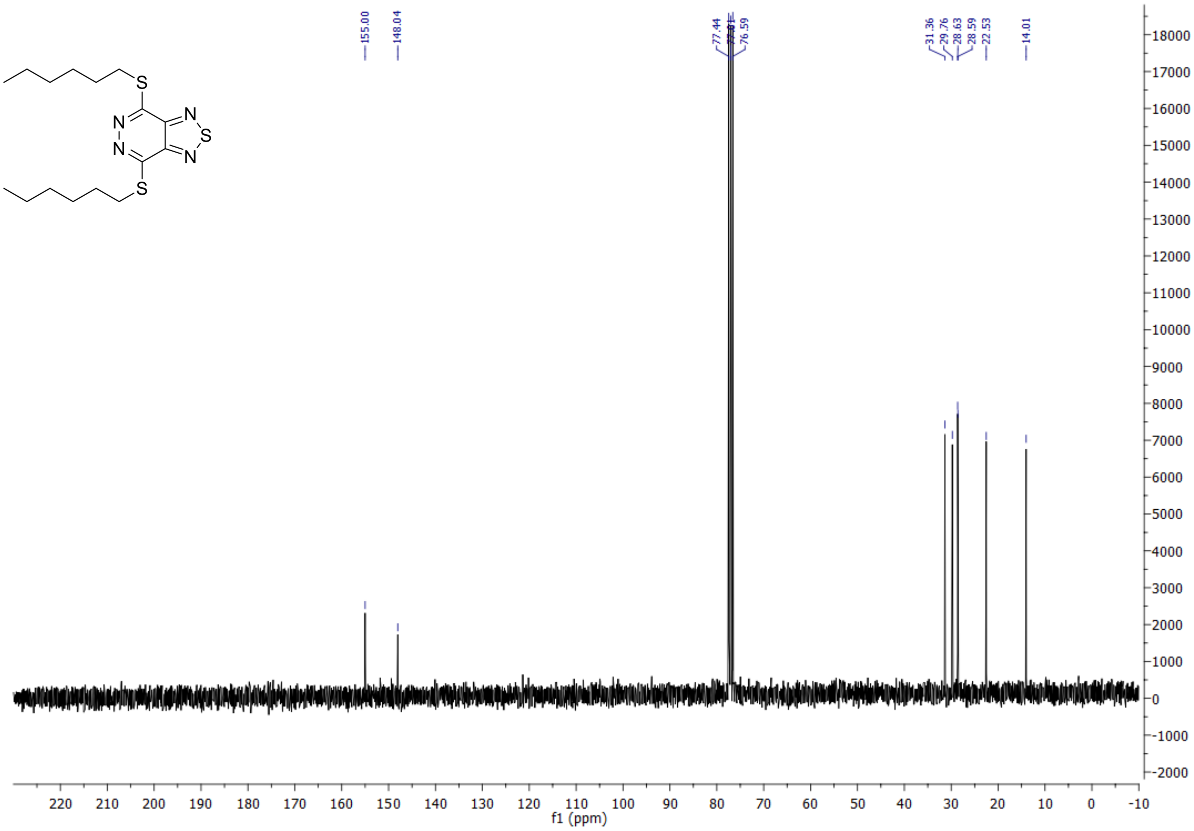

4,7-Bis(dodecylthio)-[1,2,5]thiadiazolo[3,4-d]pyridazine (10c)

^{1}H-NMR(300 MHz)

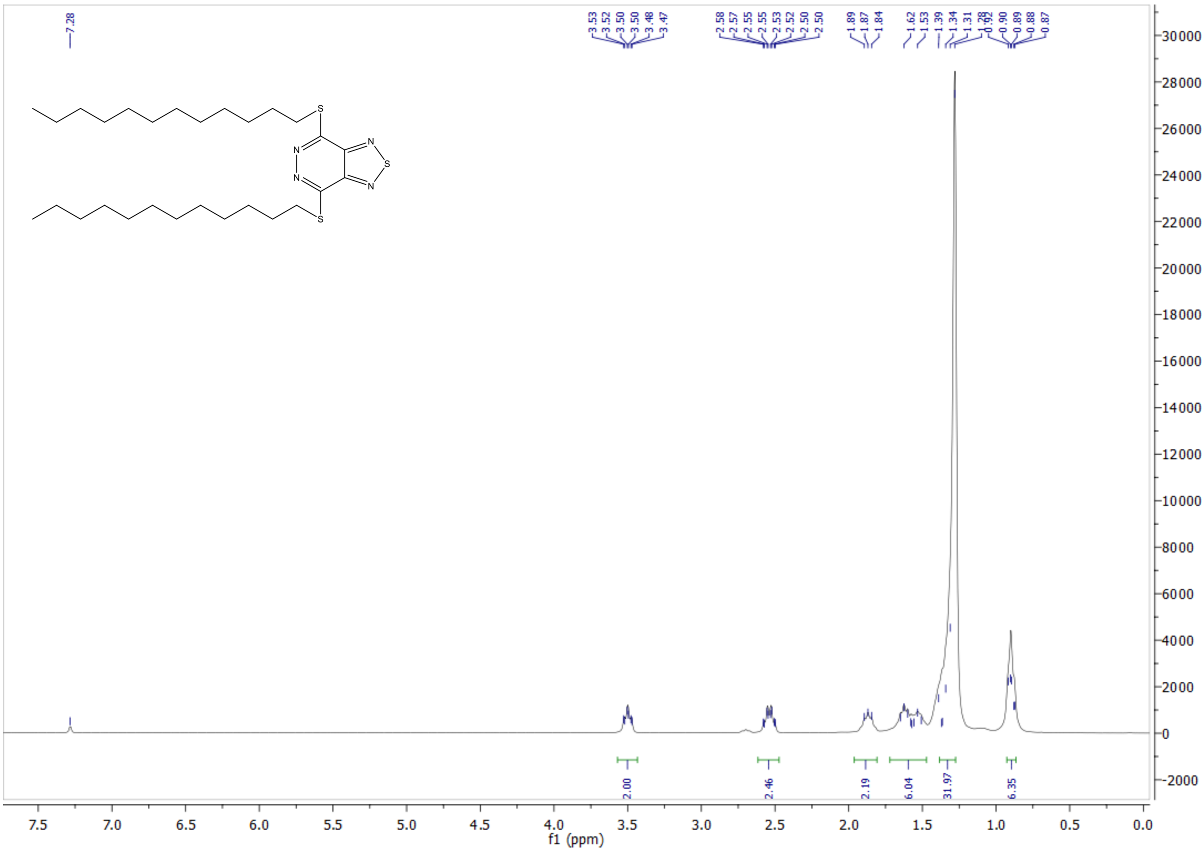

^{13}C-NMR(75 MHz)

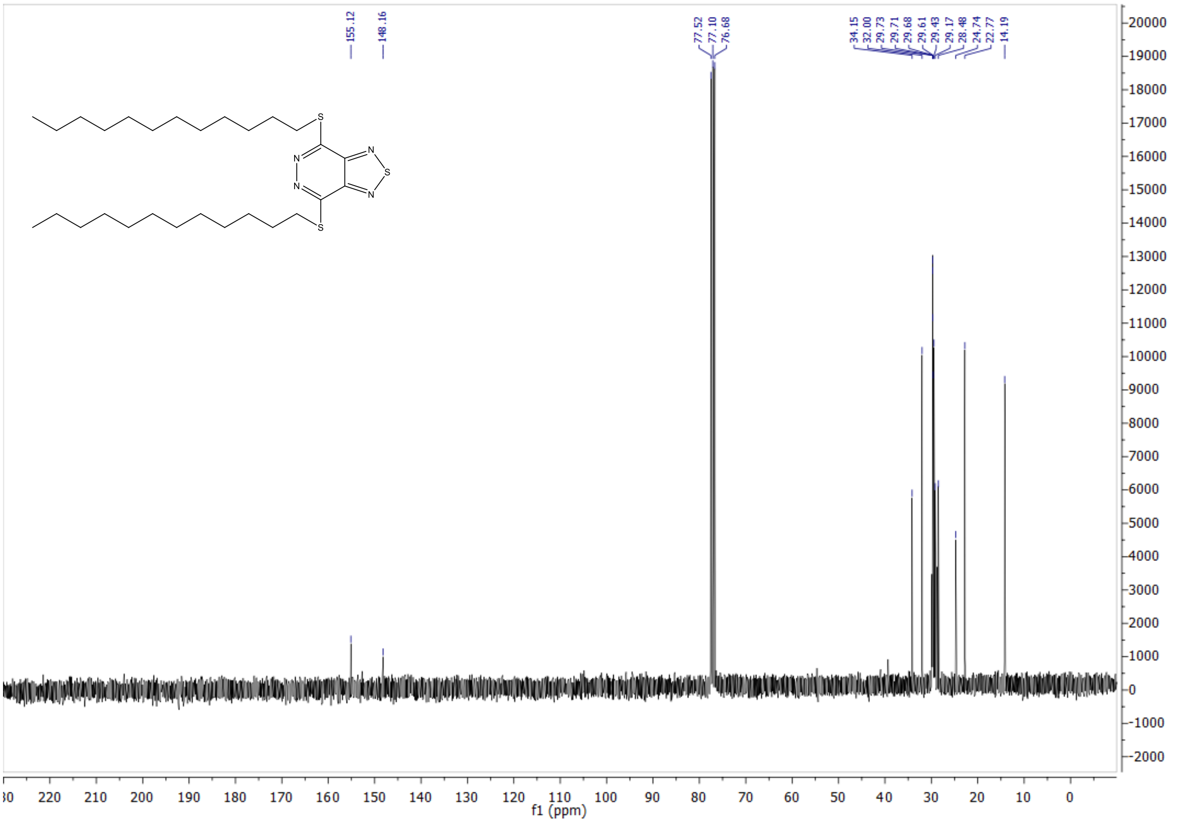

4-(7-Bromo-[1,2,5]thiadiazolo[3,4-d]pyridazin-4-yl)morpholine (11a)

^{1}H-NMR(300 MHz)

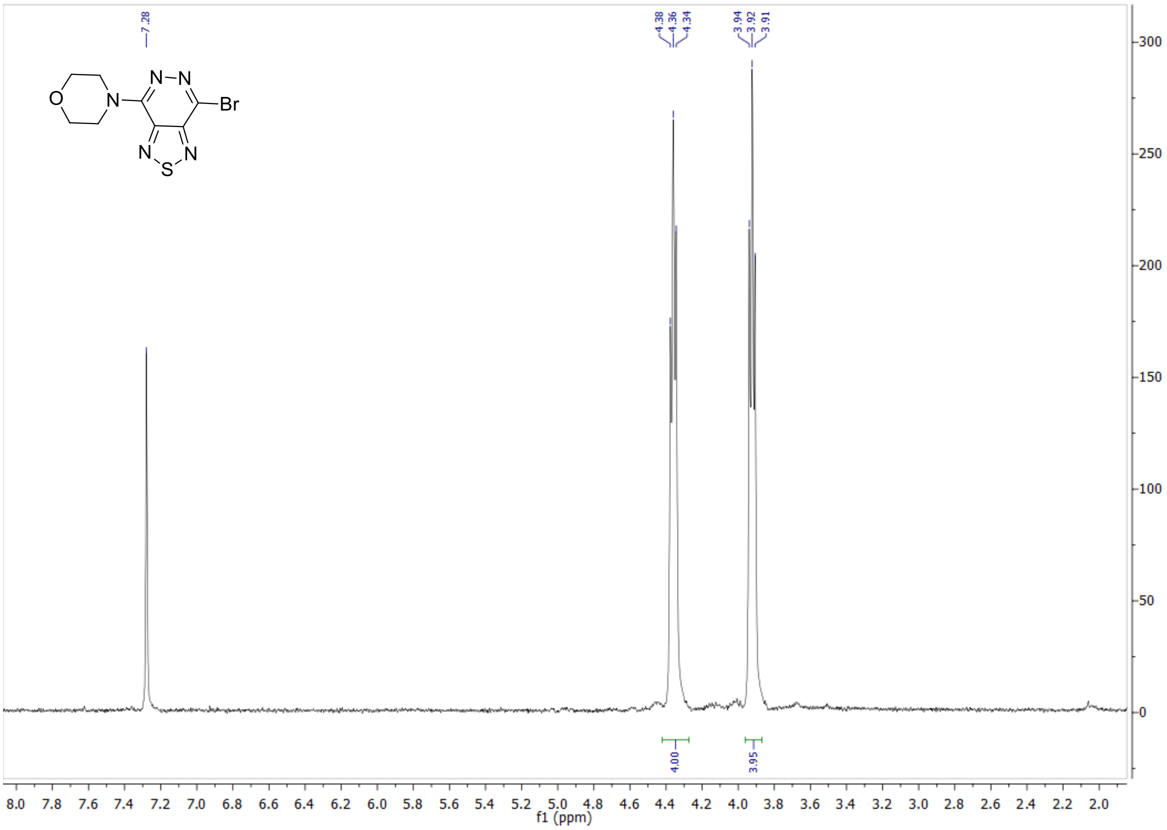

^{13}C-NMR(75 MHz)

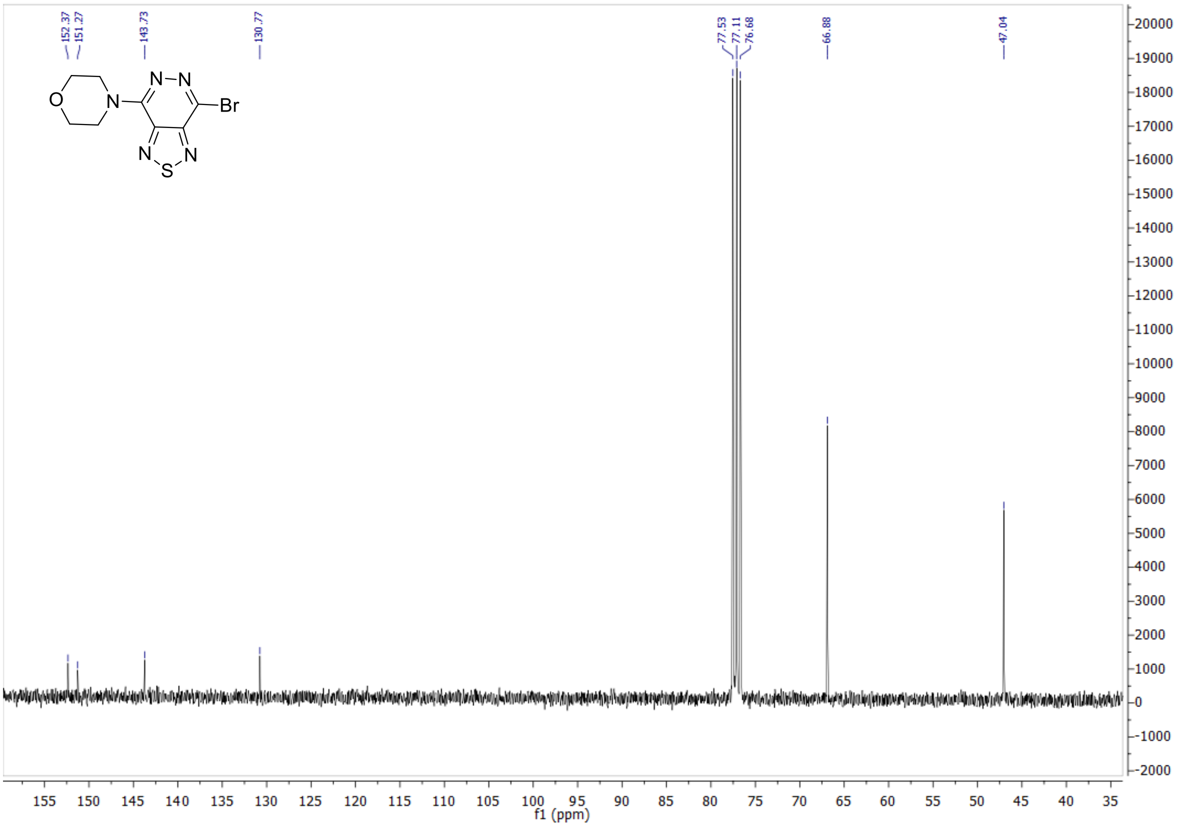

4-Bromo-7-(piperidin-1-yl)-[1,2,5]thiadiazolo[3,4-d]pyridazine (11b)

^{1}H-NMR(300 MHz)

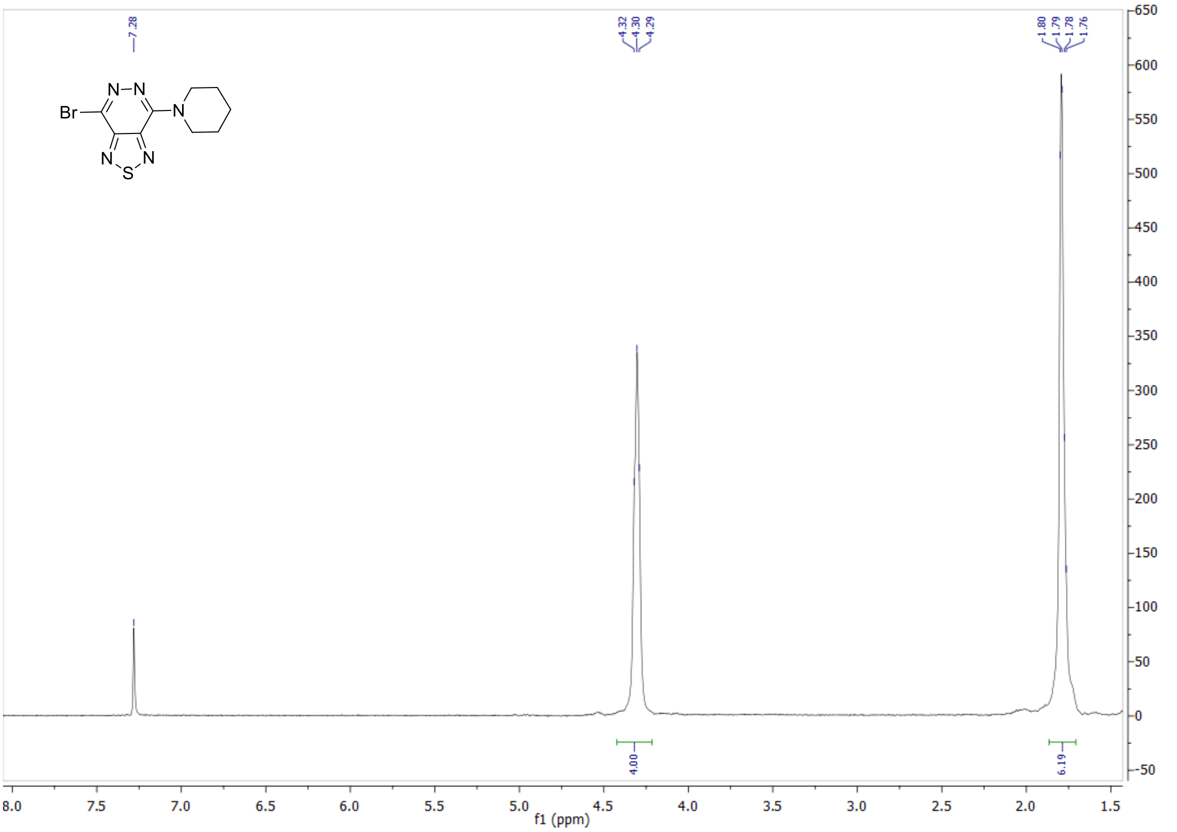

^{13}C-NMR(75 MHz)

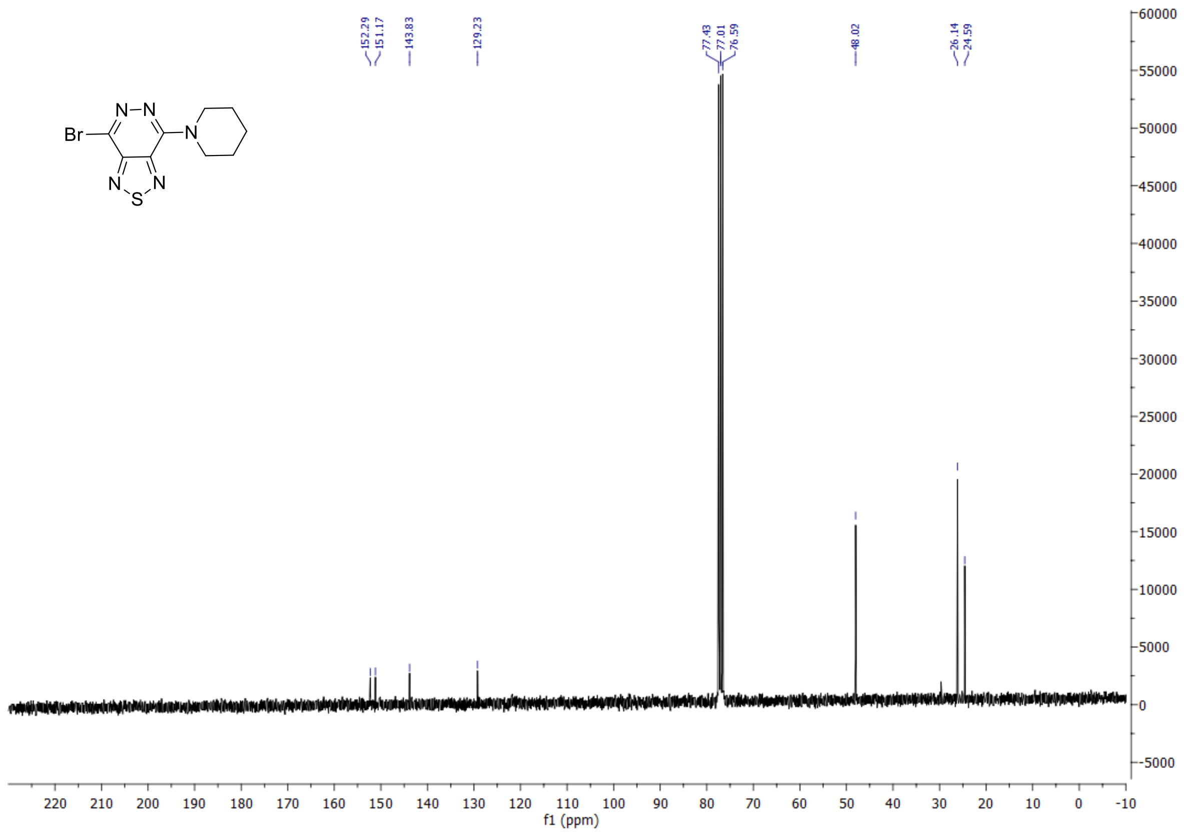

4-Bromo-7-(pyrrolidin-1-yl)-[1,2,5]thiadiazolo[3,4-d]pyridazine (11c)

^{1}H-NMR(300 MHz)

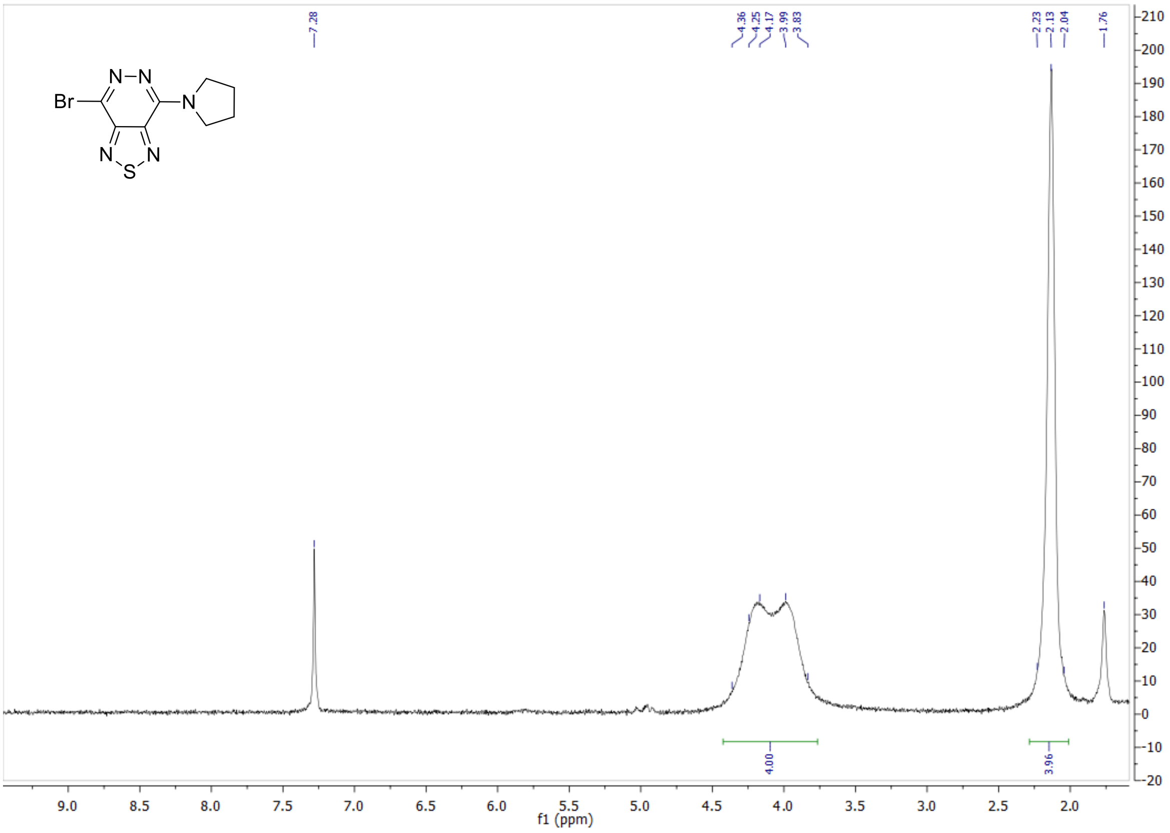

^{13}C-NMR(75 MHz)

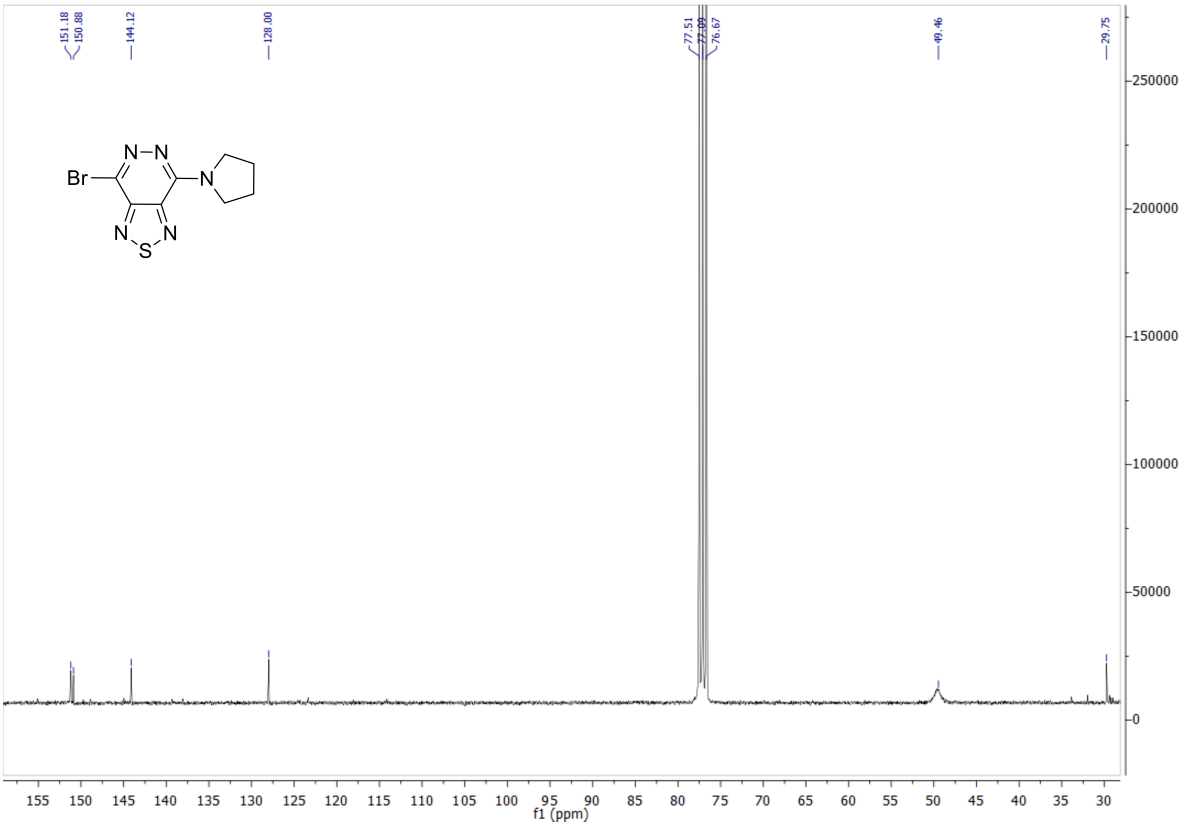

4-Bromo-7-(2,3,4,4a-tetrahydro-1H-carbazol-9(9aH)-yl)-[1,2,5]thiadiazolo[3,4-d]pyridazine (11d)

^{1}H-NMR(300 MHz)

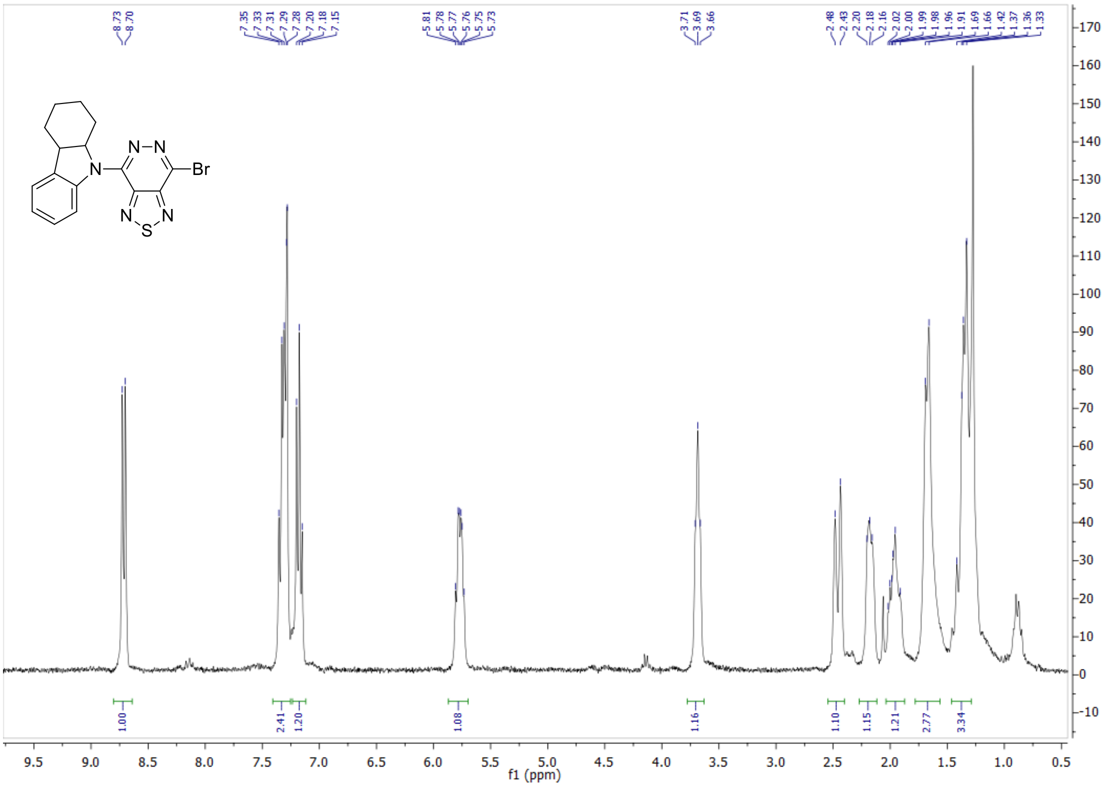

^{13}C-NMR(75 MHz)

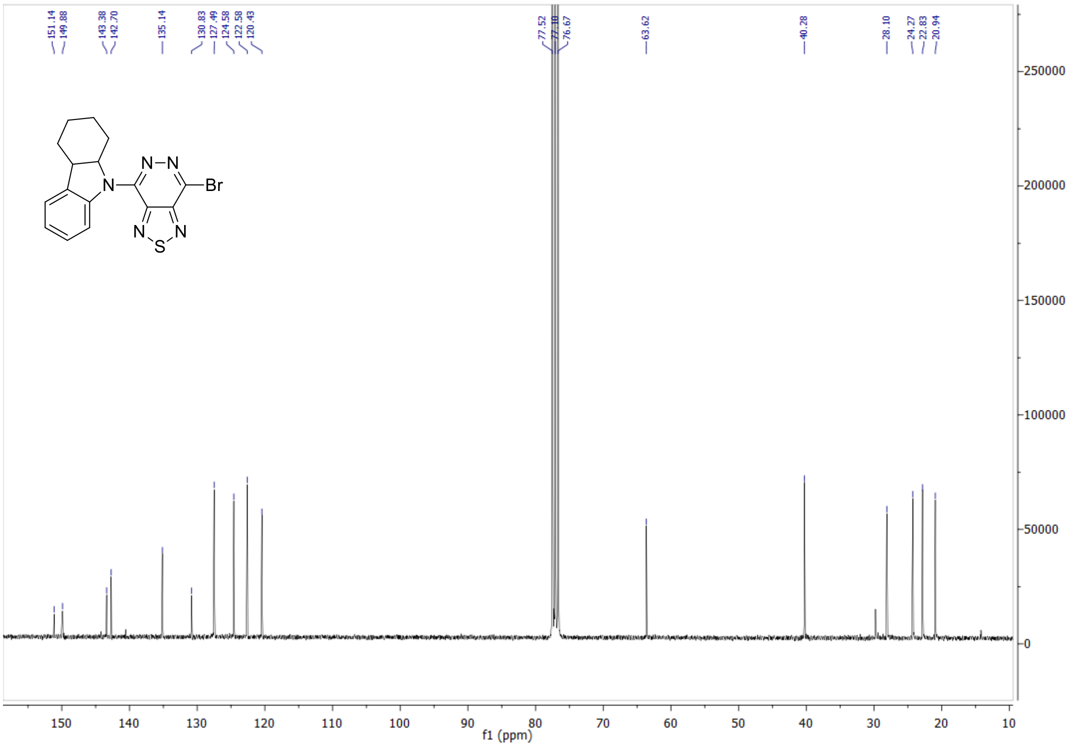

4-Bromo-7-(1,3,3a,8b-tetrahydrocyclopenta[b]indol-4(2H)-yl)-[1,2,5]thiadiazolo[3,4-d]pyridazine (11e)

^{1}H-NMR(300 MHz)

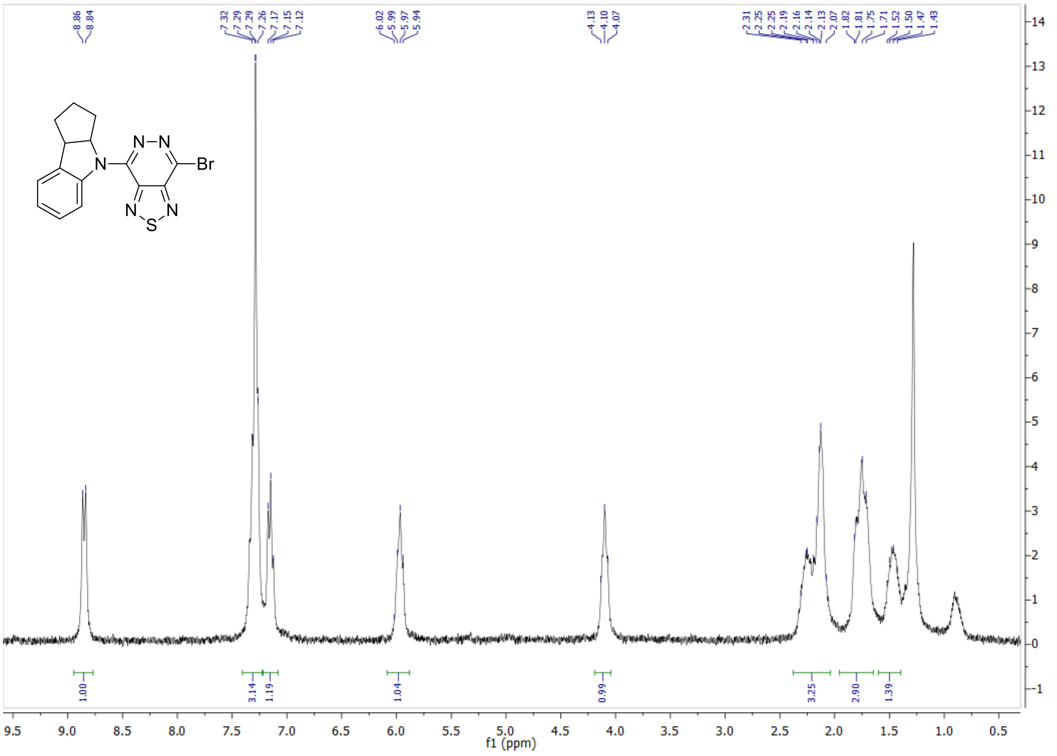

^{13}C-NMR(75 MHz)

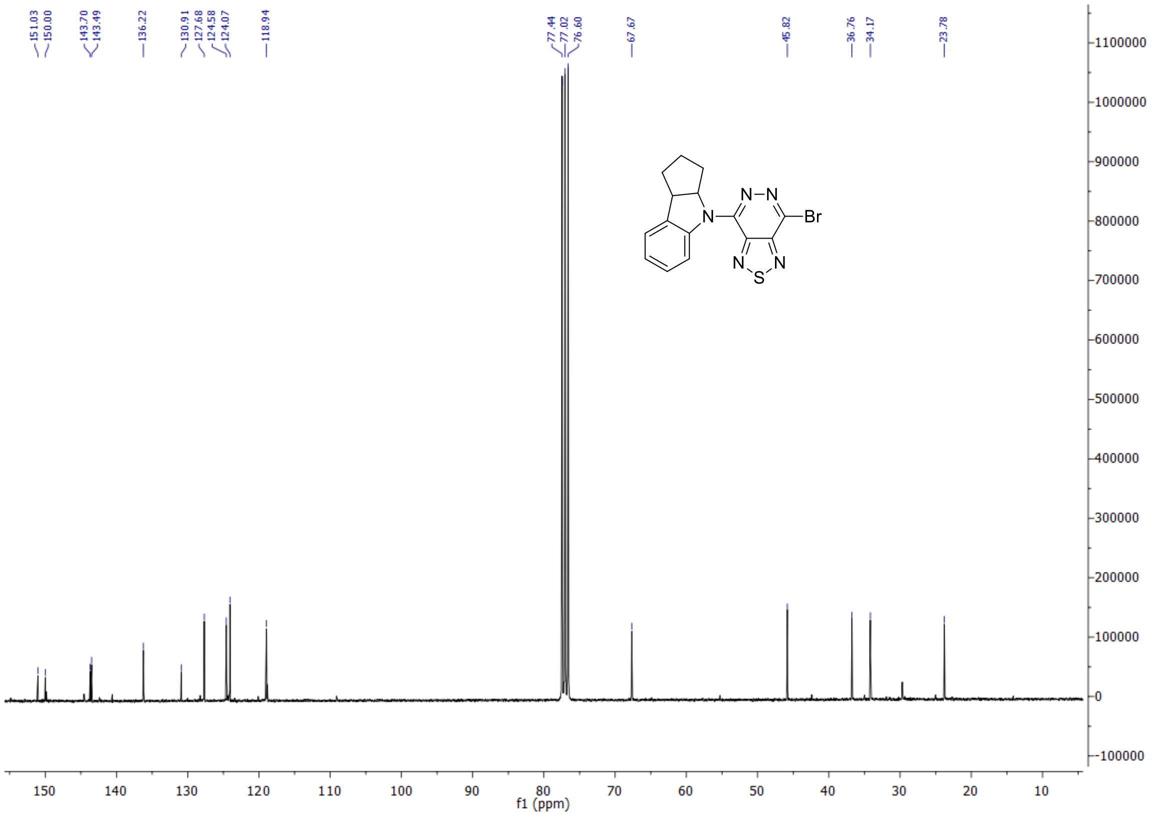

4-Bromo-7-(2,3,4,4a-tetrahydro-1H-1,4-methanocarbazol-9(9aH)-yl)-[1,2,5]thiadiazolo[3,4-d]pyridazine (11f)

^{1}H-NMR(300 MHz)

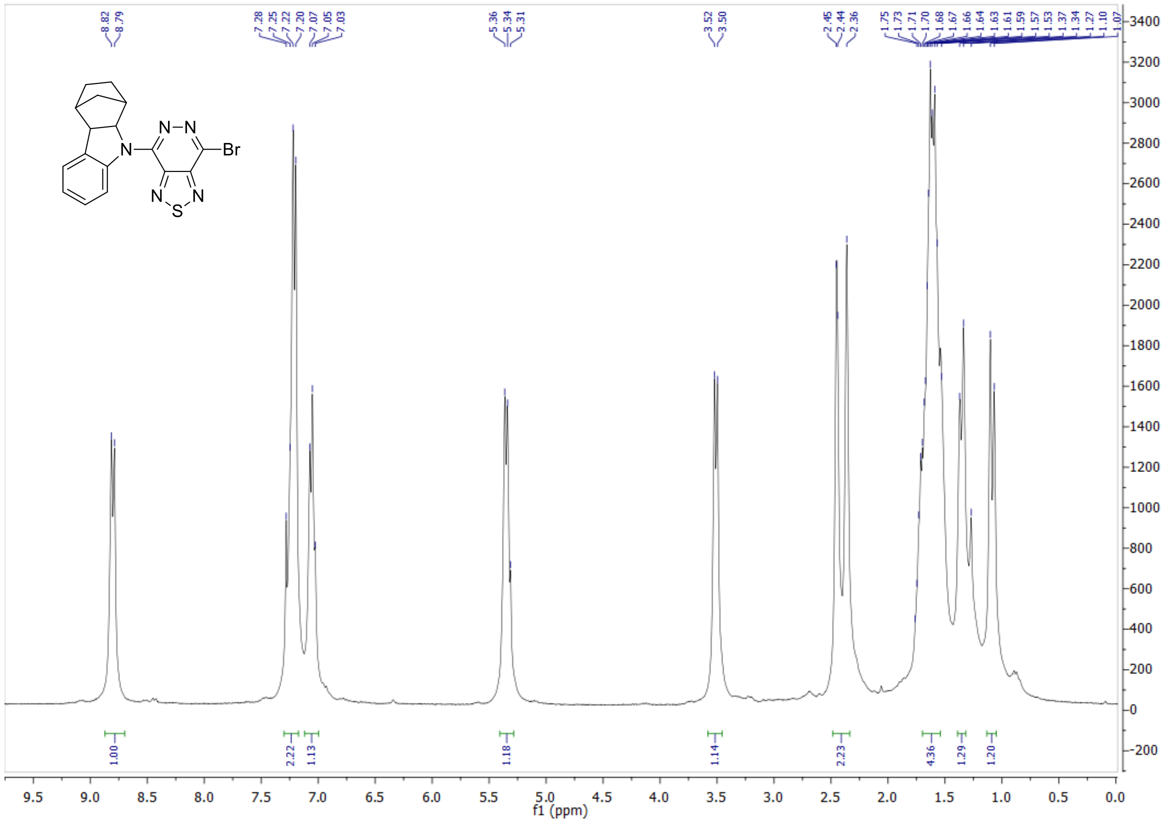

^{13}C-NMR(75 MHz)

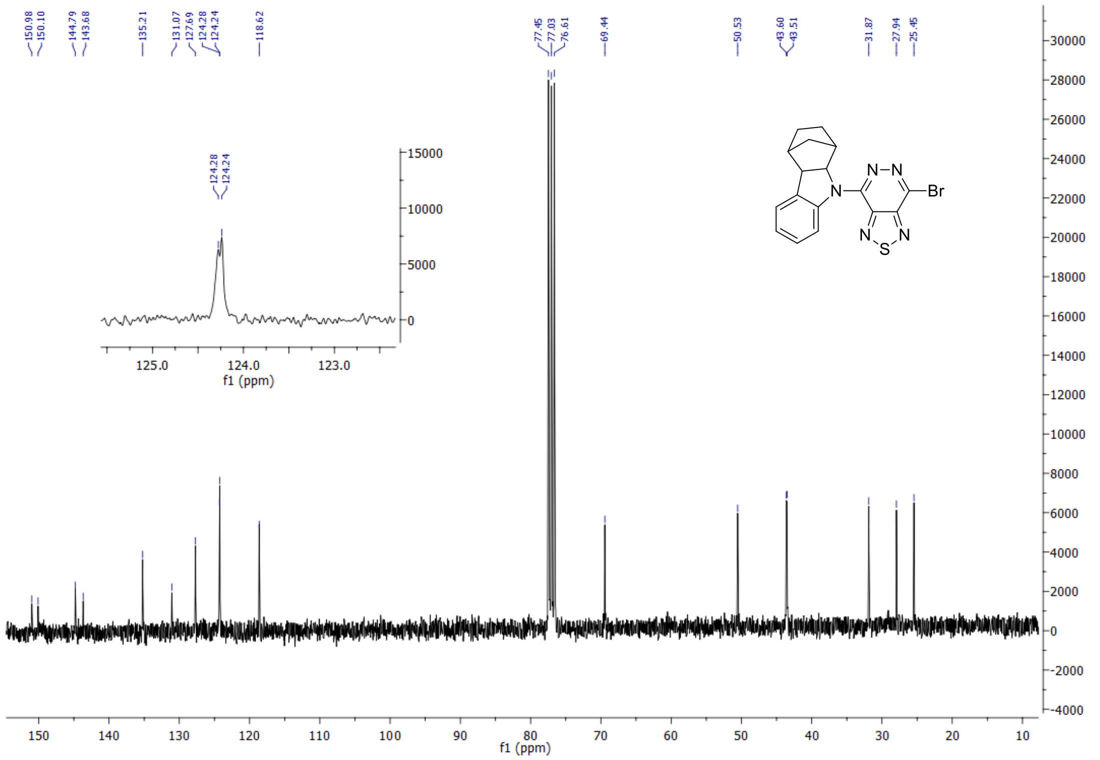

7-Bromo-*N*-methyl-*N*-phenyl-[1,2,5]thiadiazolo[3,4-d]pyridazin-4-amine (11g)

^{1}H-NMR(300 MHz)

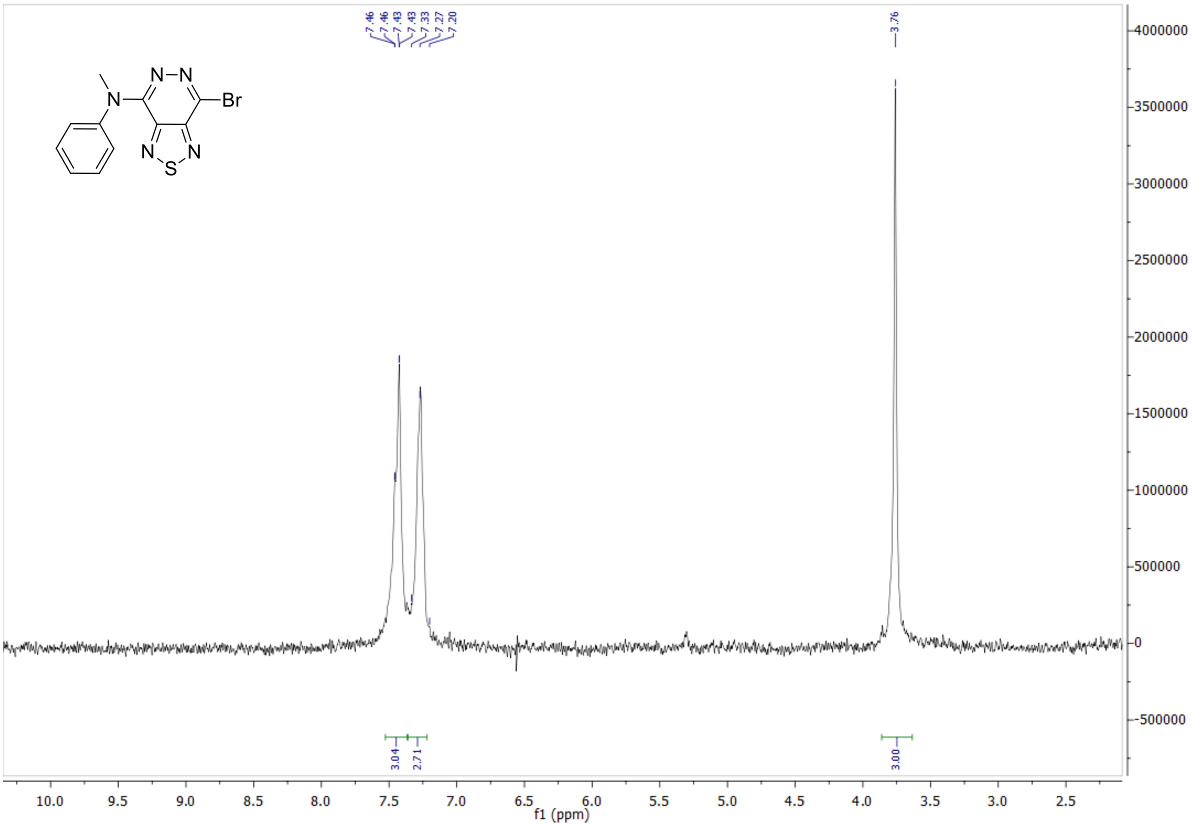

^{13}C-NMR(75 MHz)

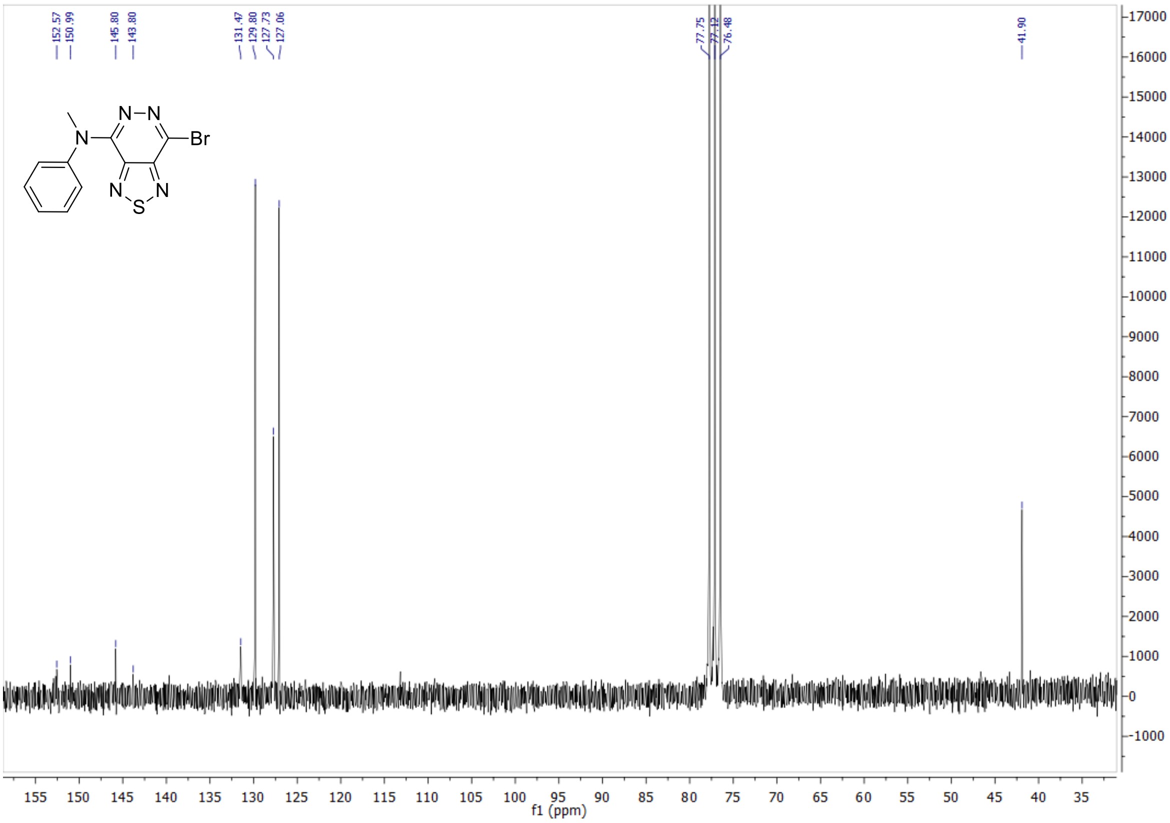

7-Bromo-*N*-cyclohexyl-[1,2,5]thiadiazolo[3,4-d]pyridazin-4-amine (11h)

^{1}H-NMR(300 MHz)

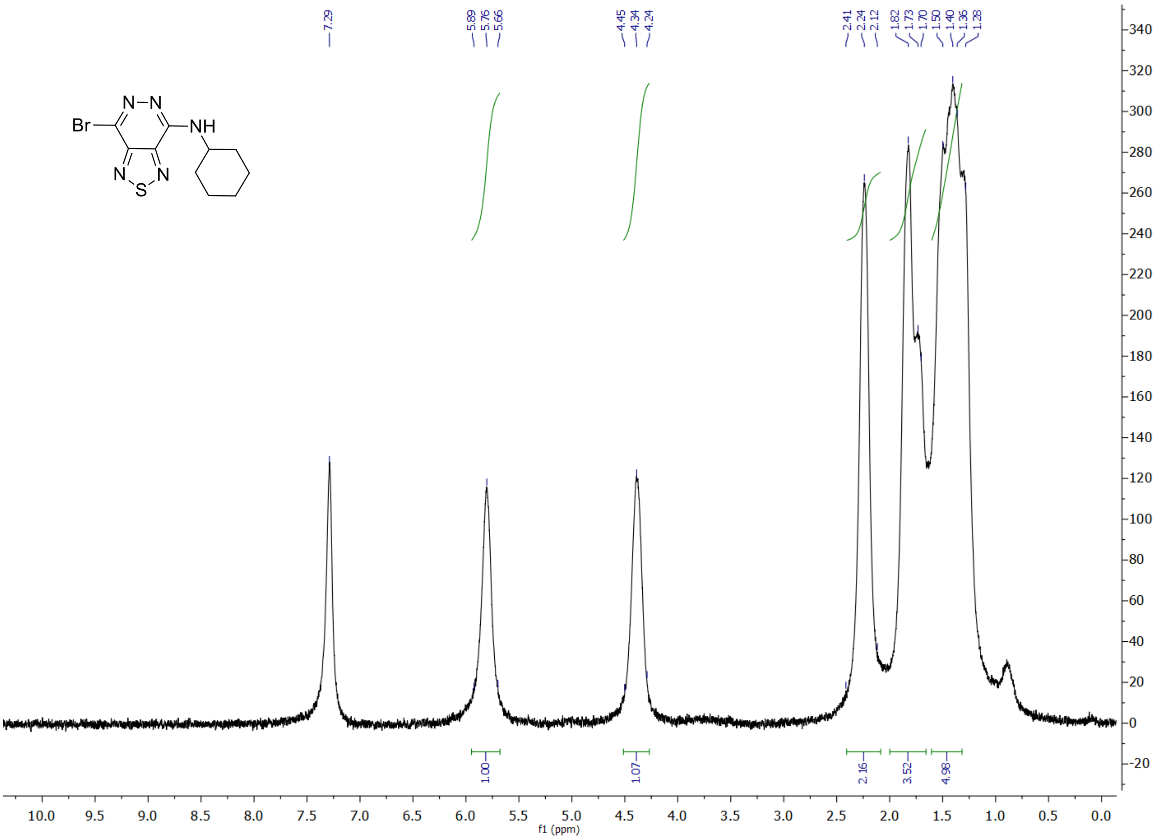

^{13}C-NMR(75 MHz)

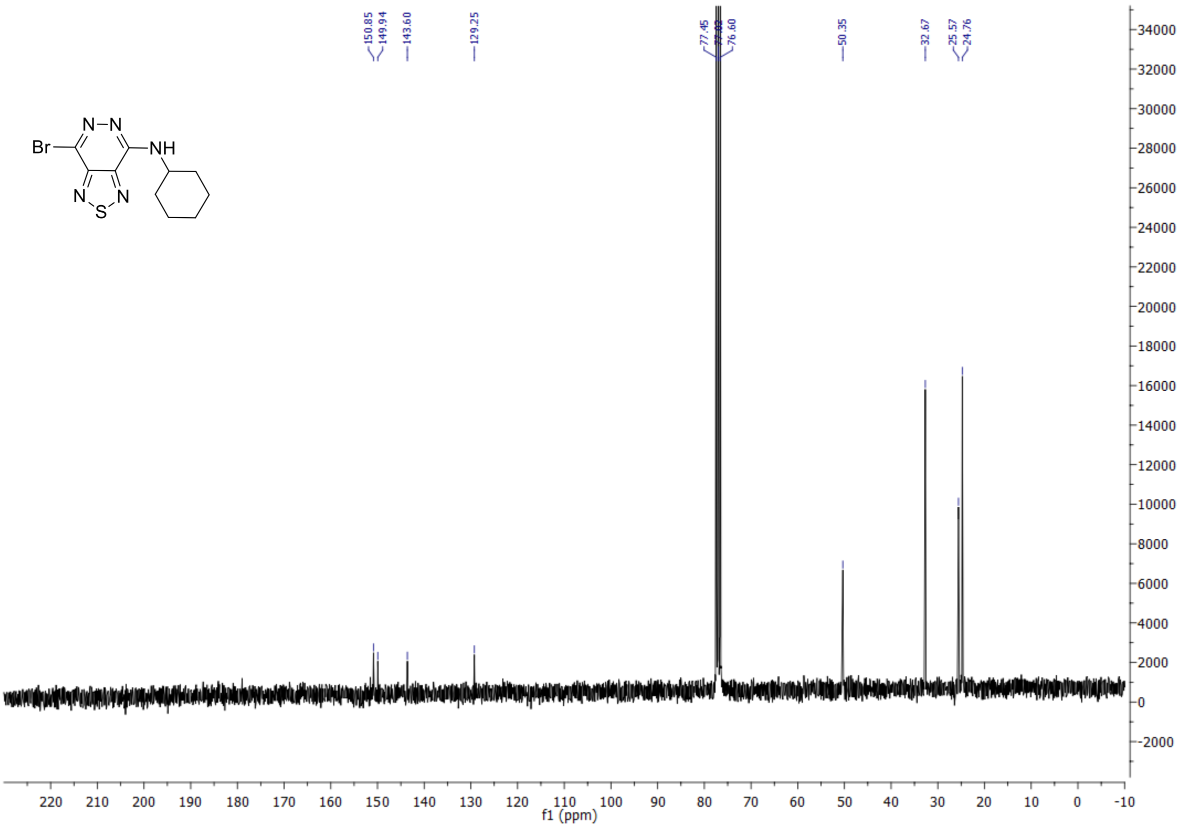

7-Bromo-*N*-phenyl-[1,2,5]thiadiazolo[3,4-d]pyridazin-4-amine (11i)

^{1}H-NMR(300 MHz)

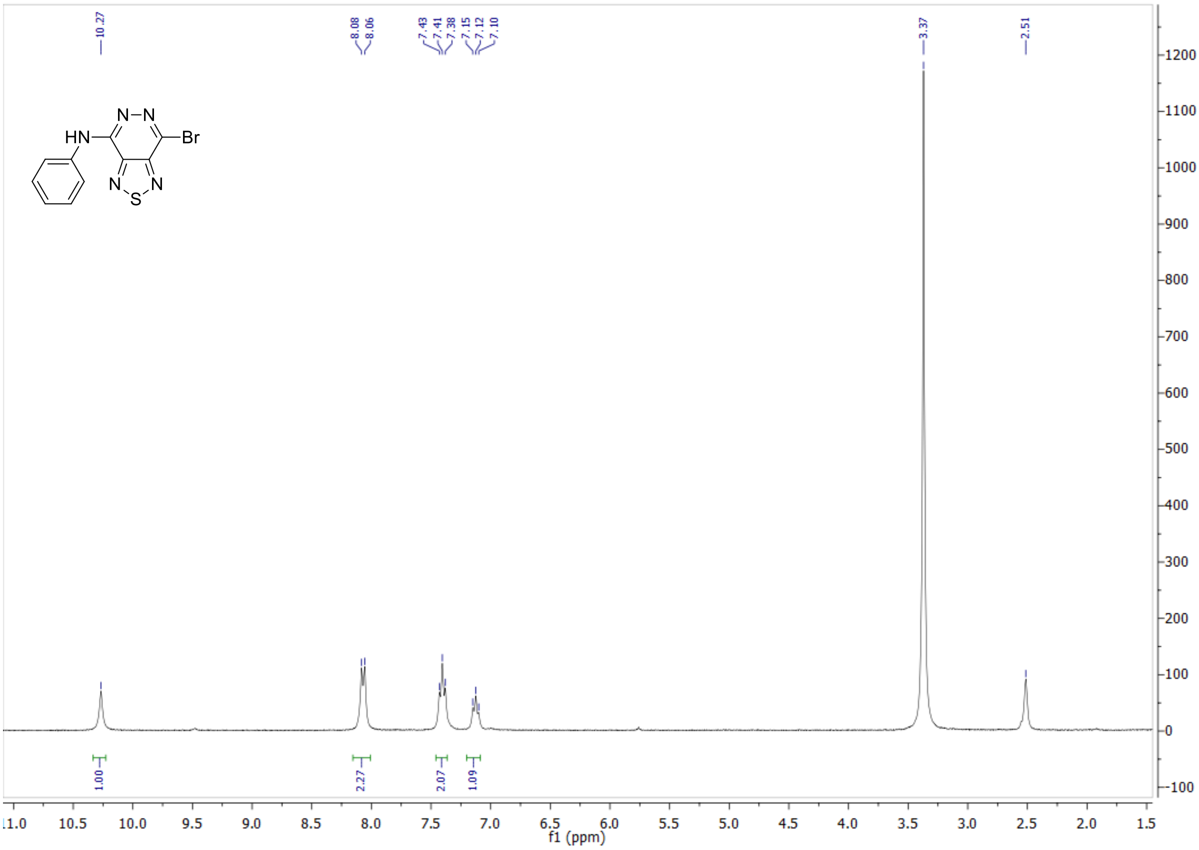

^{13}C-NMR(75 MHz)

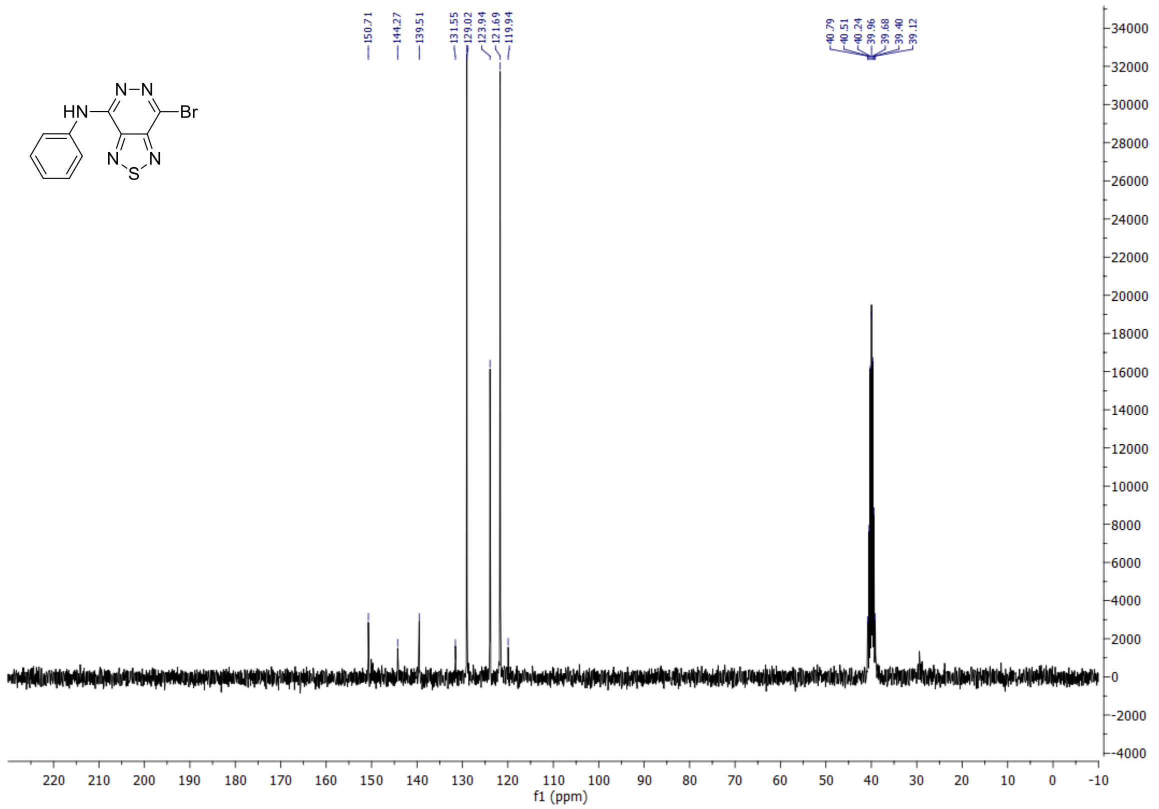

7-Bromo-*N*-(tert-butyl)-[1,2,5]thiadiazolo[3,4-d]pyridazin-4-amine (11j)

^{1}H-NMR(300 MHz)

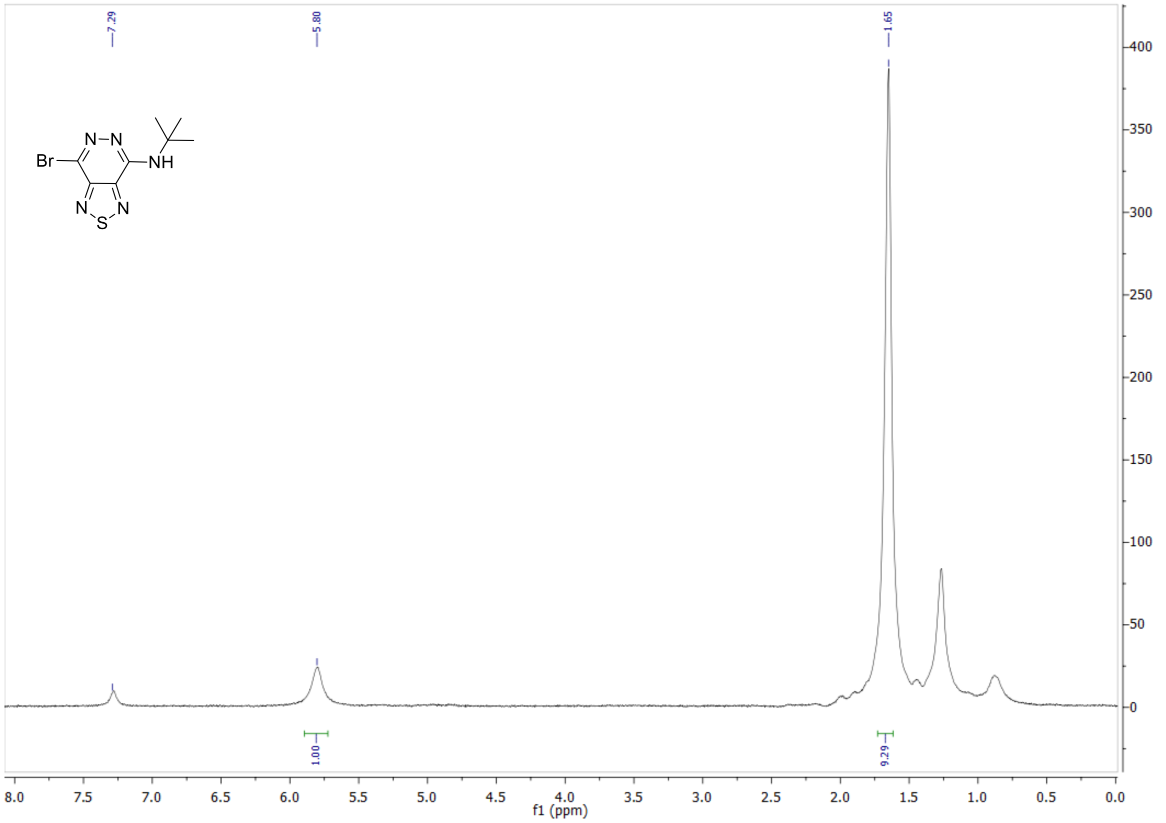

^{13}C-NMR(75 MHz)

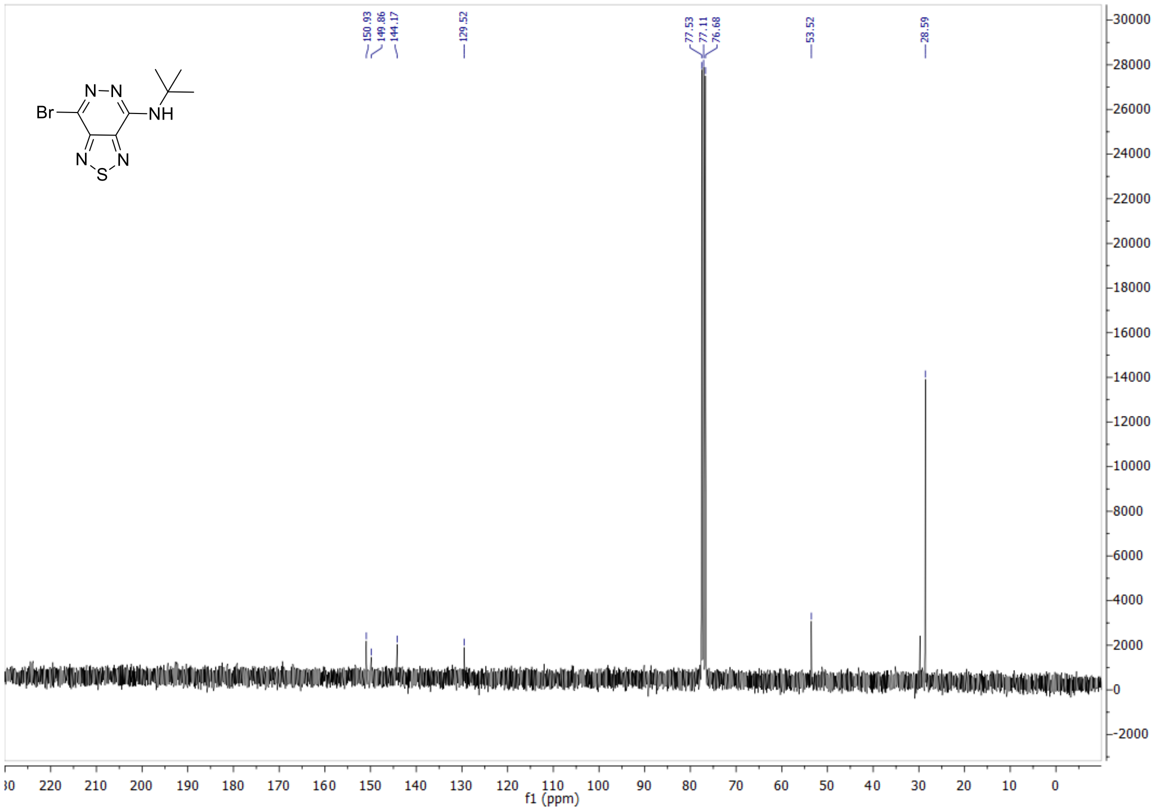

4,7-Dimorpholino-[1,2,5]thiadiazolo[3,4-d]pyridazine (12a)

^{1}H-NMR(300 MHz)

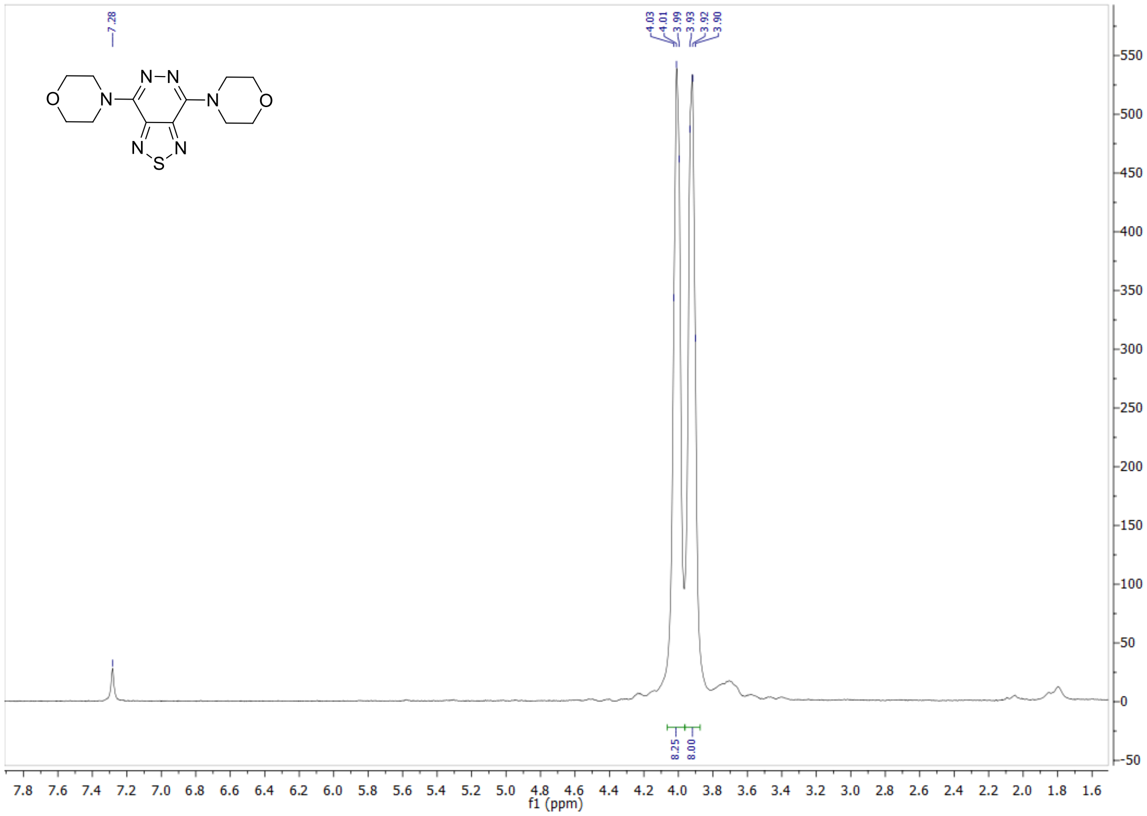

^{13}C-NMR(75 MHz)

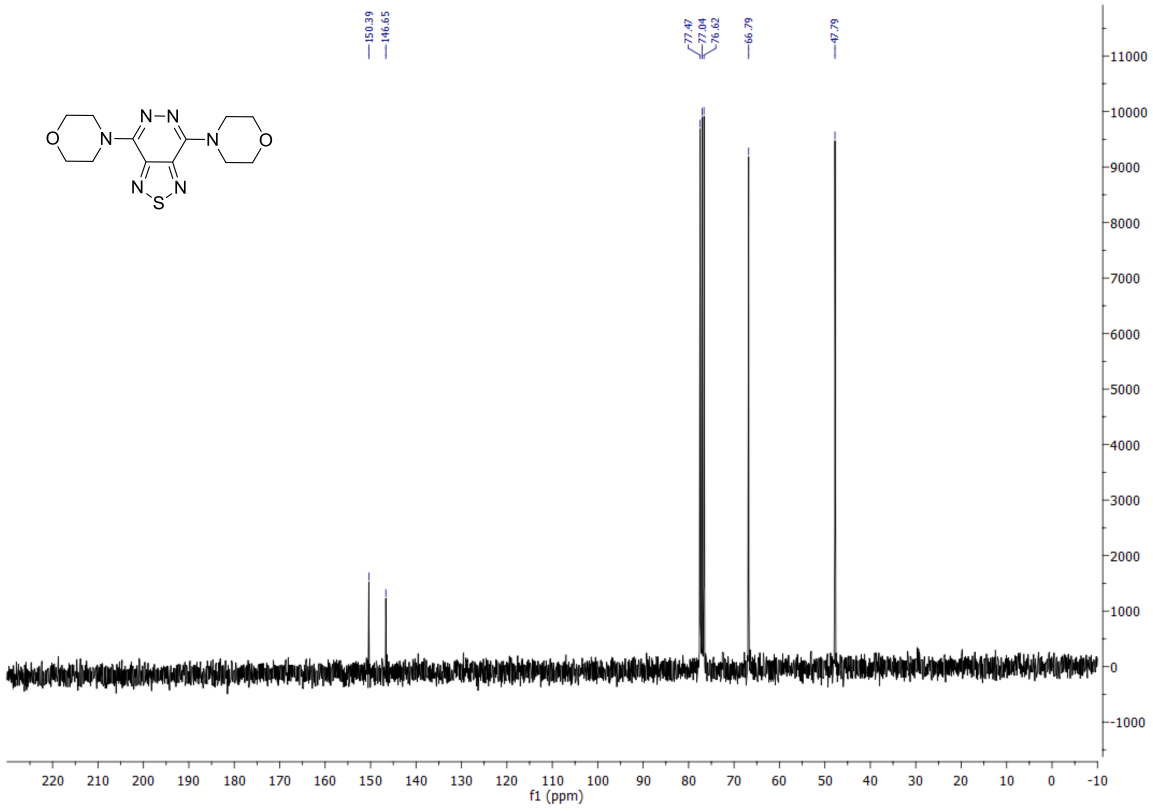

4,7-Di(piperidin-1-yl)-[1,2,5]thiadiazolo[3,4-d]pyridazine (12b)

^{1}H-NMR(300 MHz)

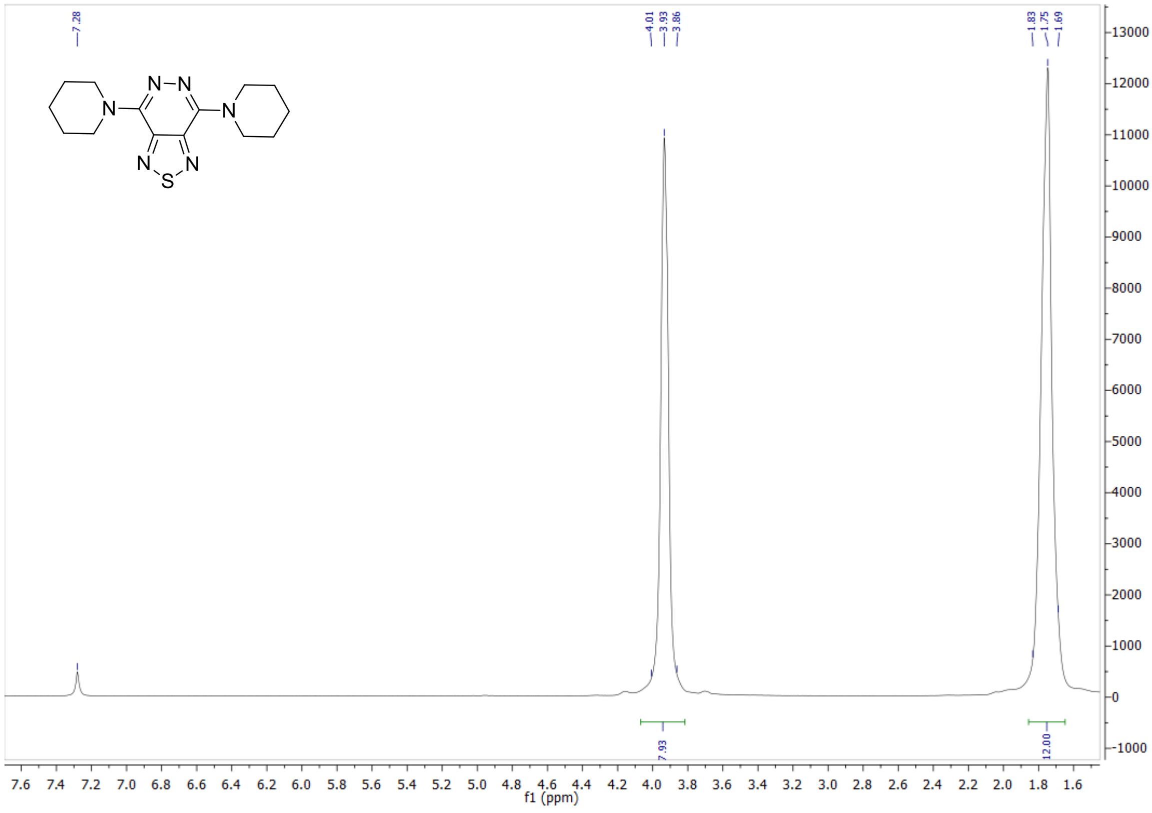

^{13}C-NMR(75 MHz)

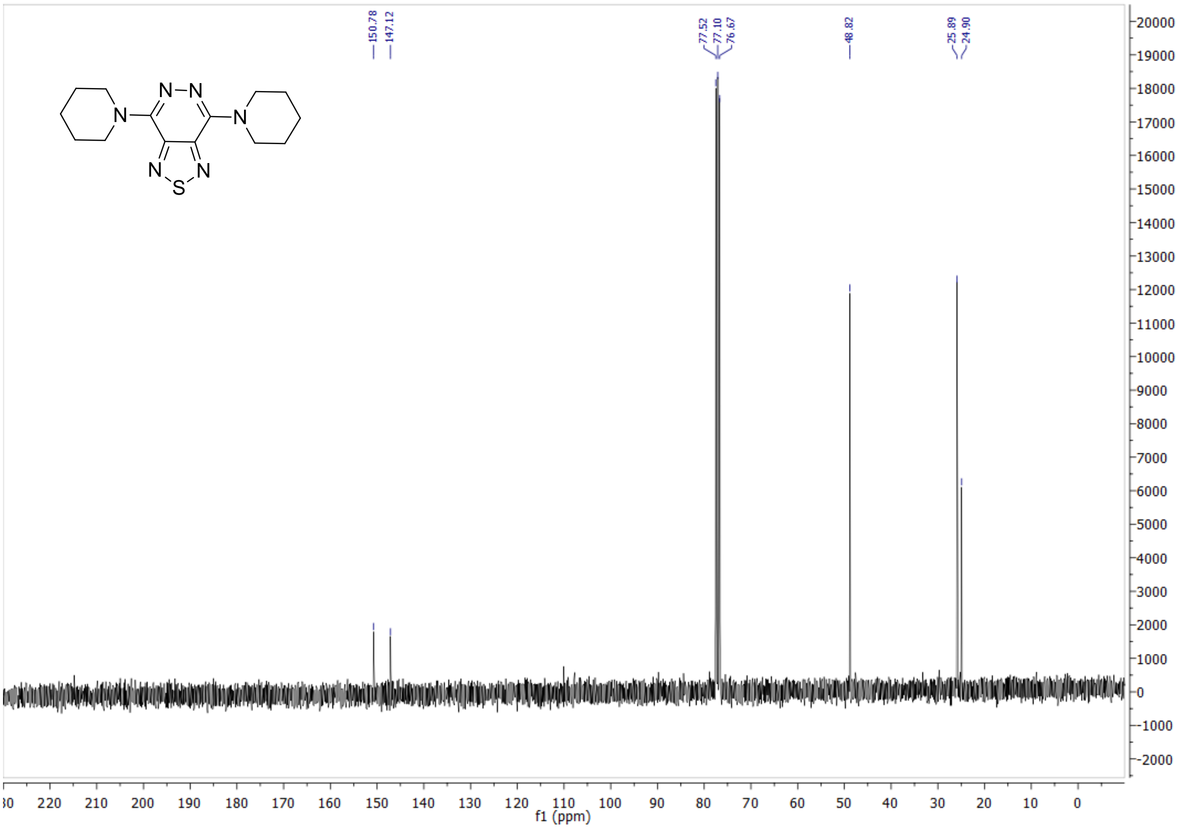

4,7-Bis(2,3,4,4a-tetrahydro-1H-carbazol-9(9aH)-yl)-[1,2,5]thiadiazolo[3,4-d]pyridazine (12d)

^{1}H-NMR(300 MHz)

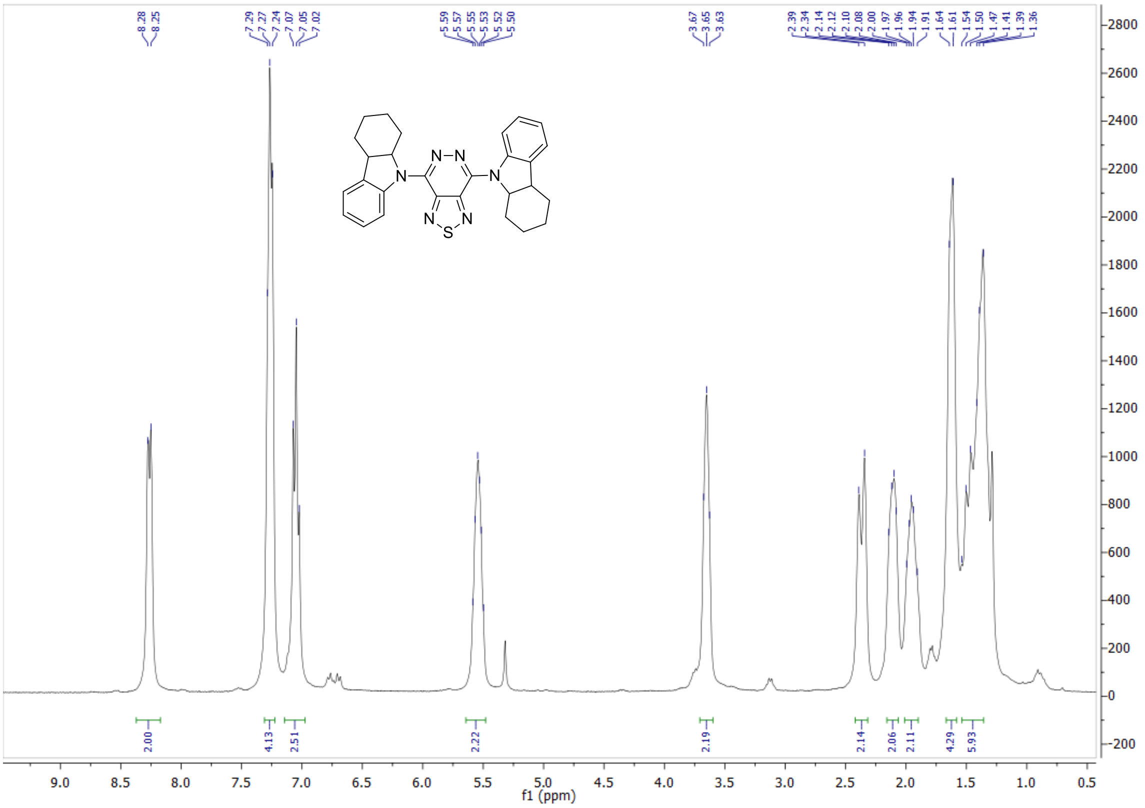

^{13}C-NMR(75 MHz)

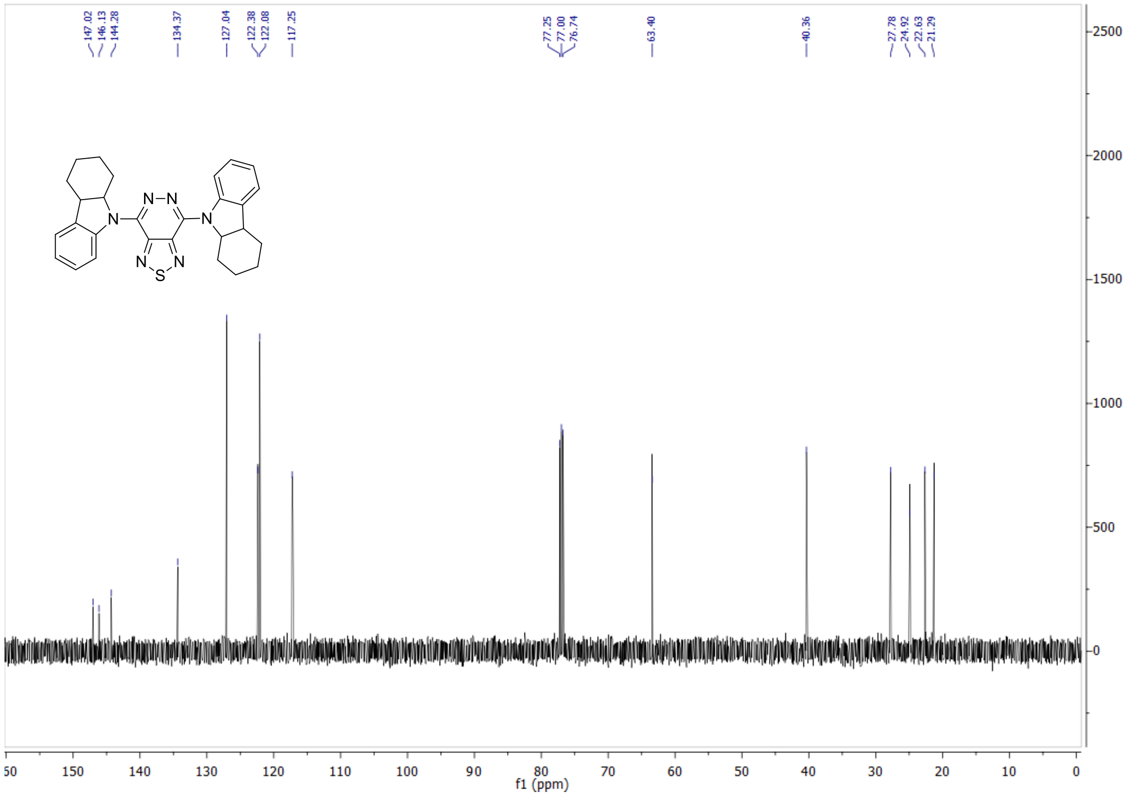

***N*^{4},*N*^{7}-Diphenyl-[1,2,5]thiadiazolo[3,4-d]pyridazine-4,7-diamine (12i)**

^{1}H-NMR(300 MHz)

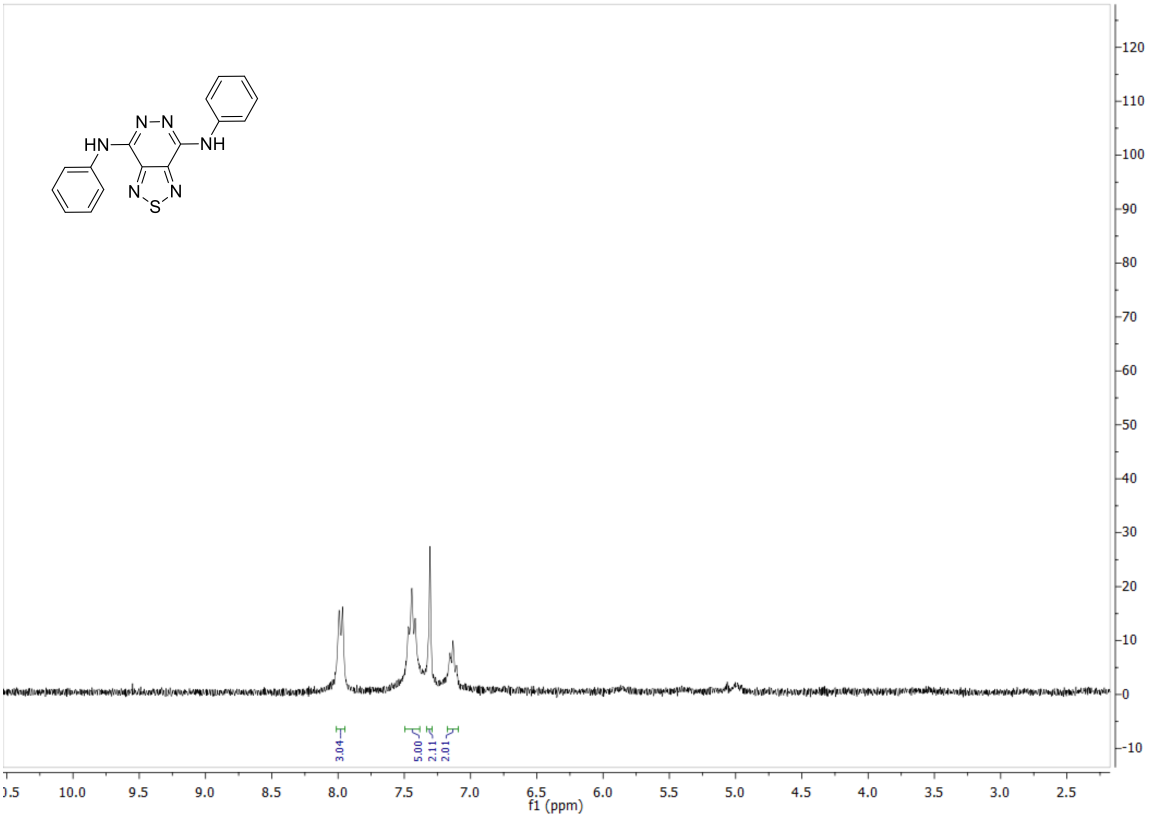

^{13}**C-NMR(75 MHz)**

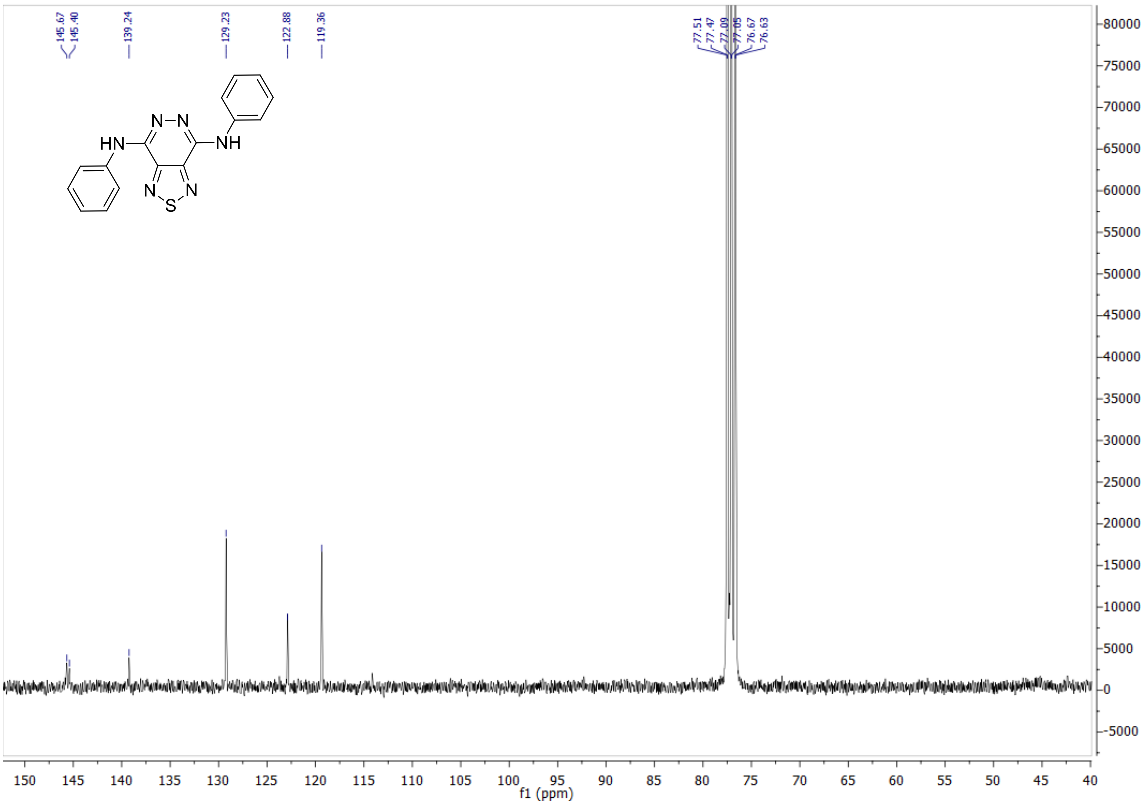

4-(7-(Pyrrolidin-1-yl)-[1,2,5]thiadiazolo[3,4-d]pyridazin-4-yl)morpholine (13)

^{1}**H-NMR(300 MHz)**

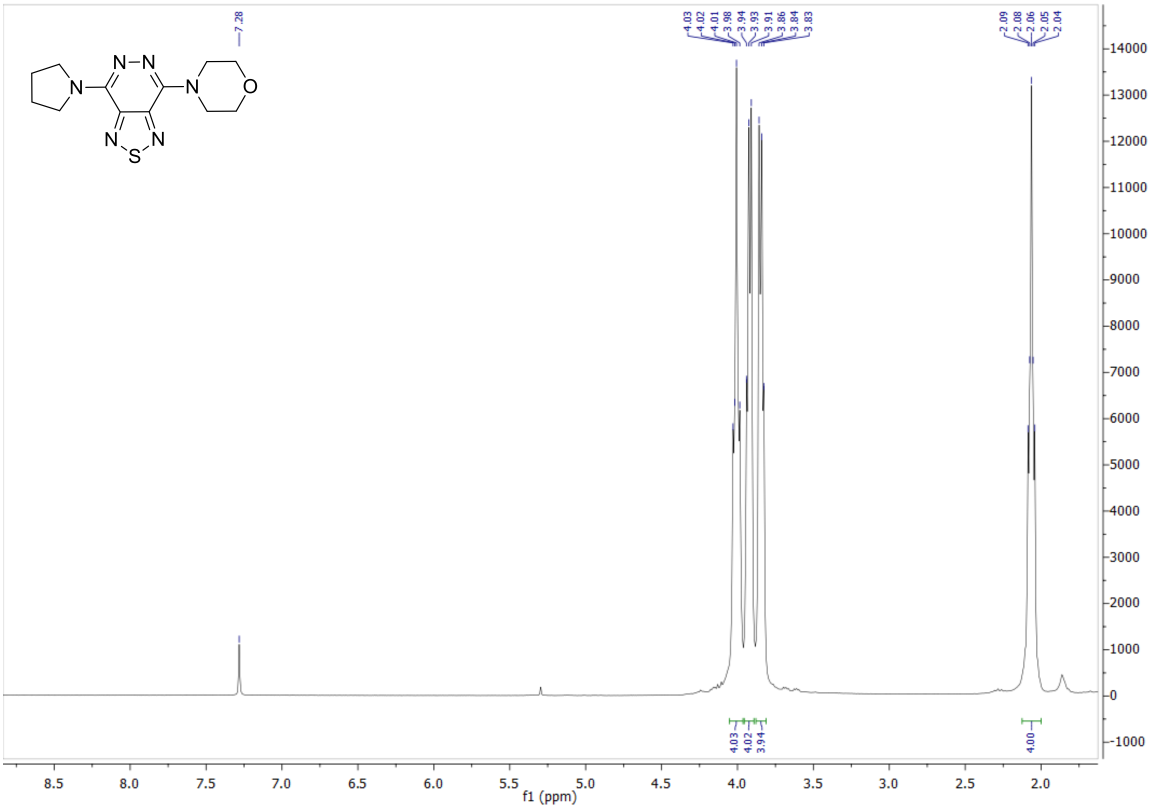

^{13}C-NMR(75 MHz)

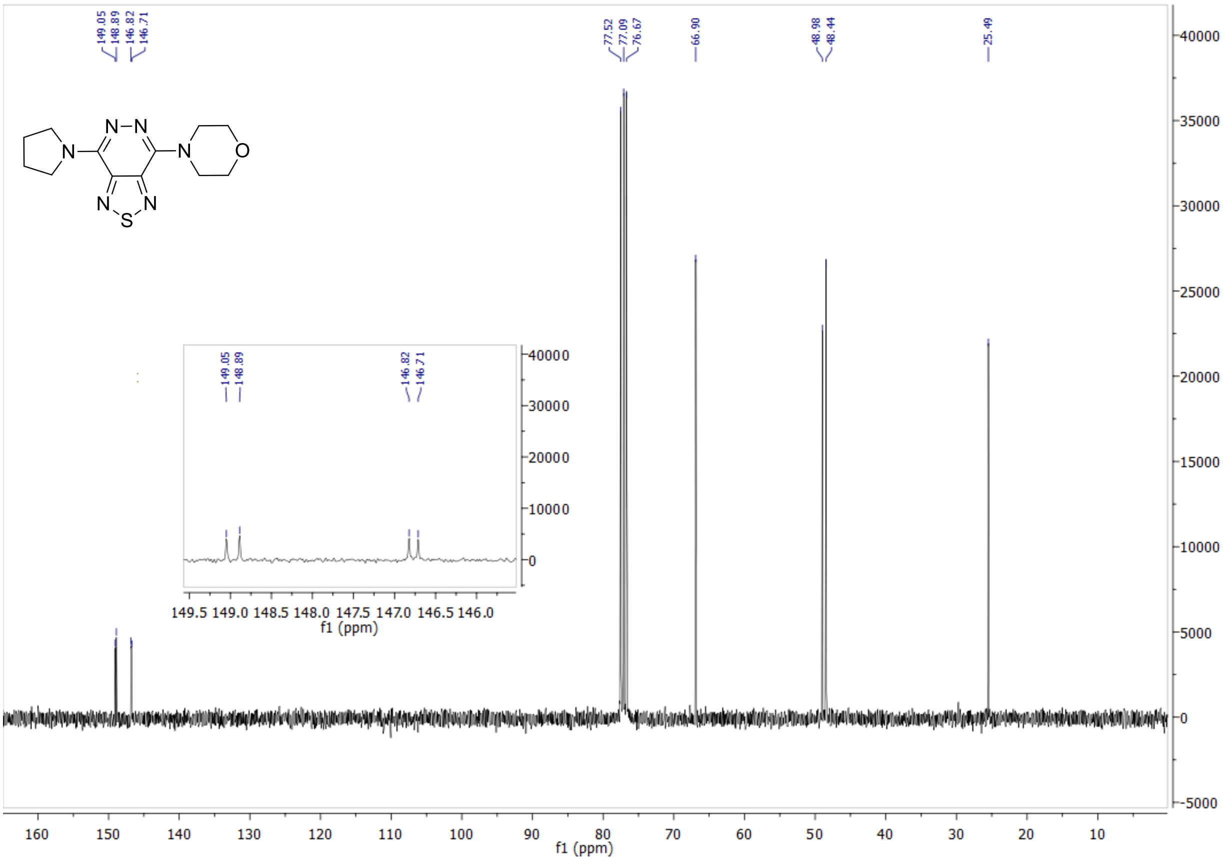

4-(7-(Phenylthio)-[1,2,5]thiadiazolo[3,4-d]pyridazin-4-yl)morpholine (14)

^{1}H-NMR(300 MHz)

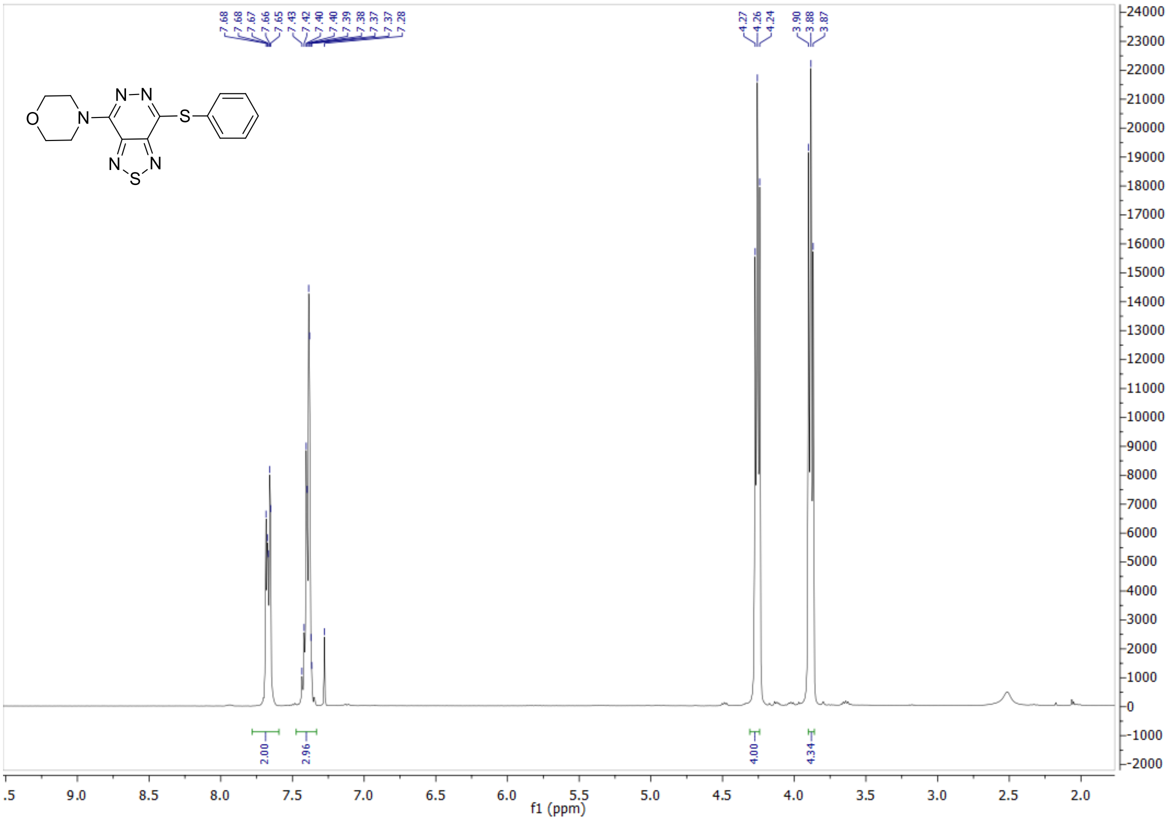

^{13}C-NMR(75 MHz)

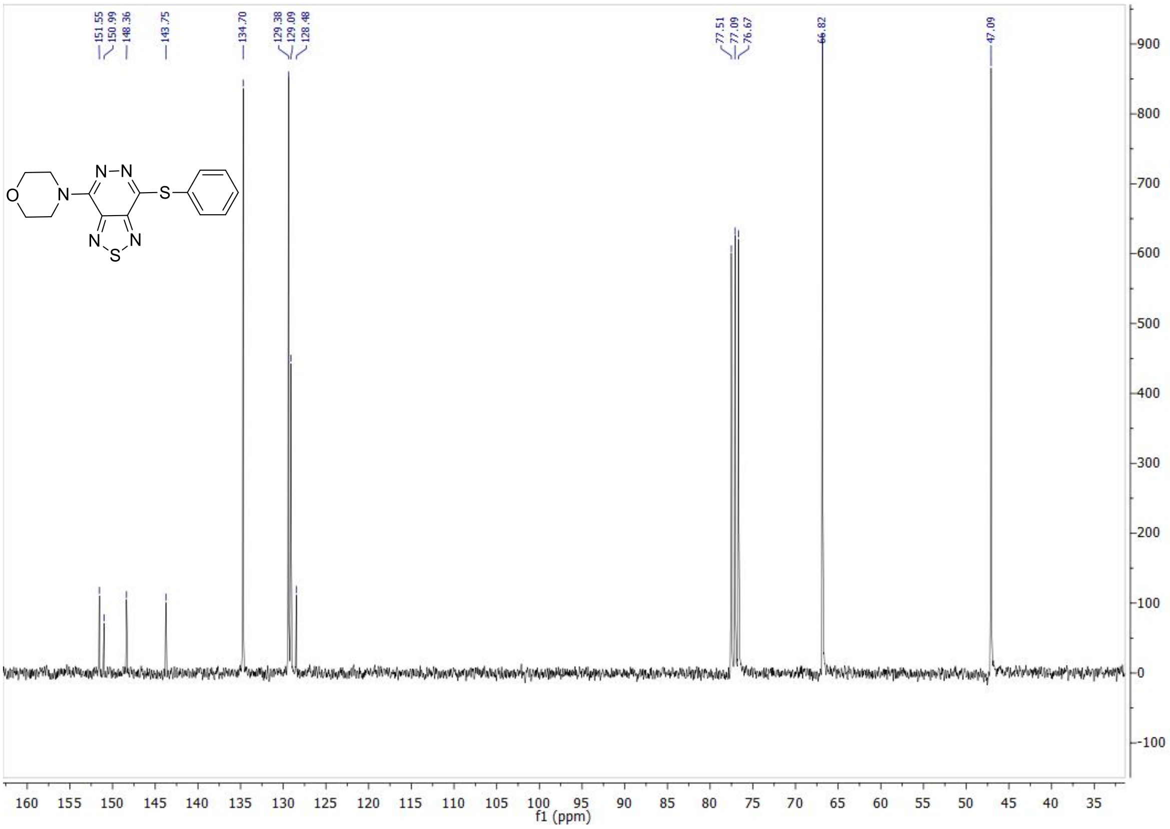

4-(7-(9H-Carbazol-9-yl)-[1,2,5]thiadiazolo[3,4-d]pyridazin-4-yl)morpholine (15)

^{1}H-NMR(300 MHz)

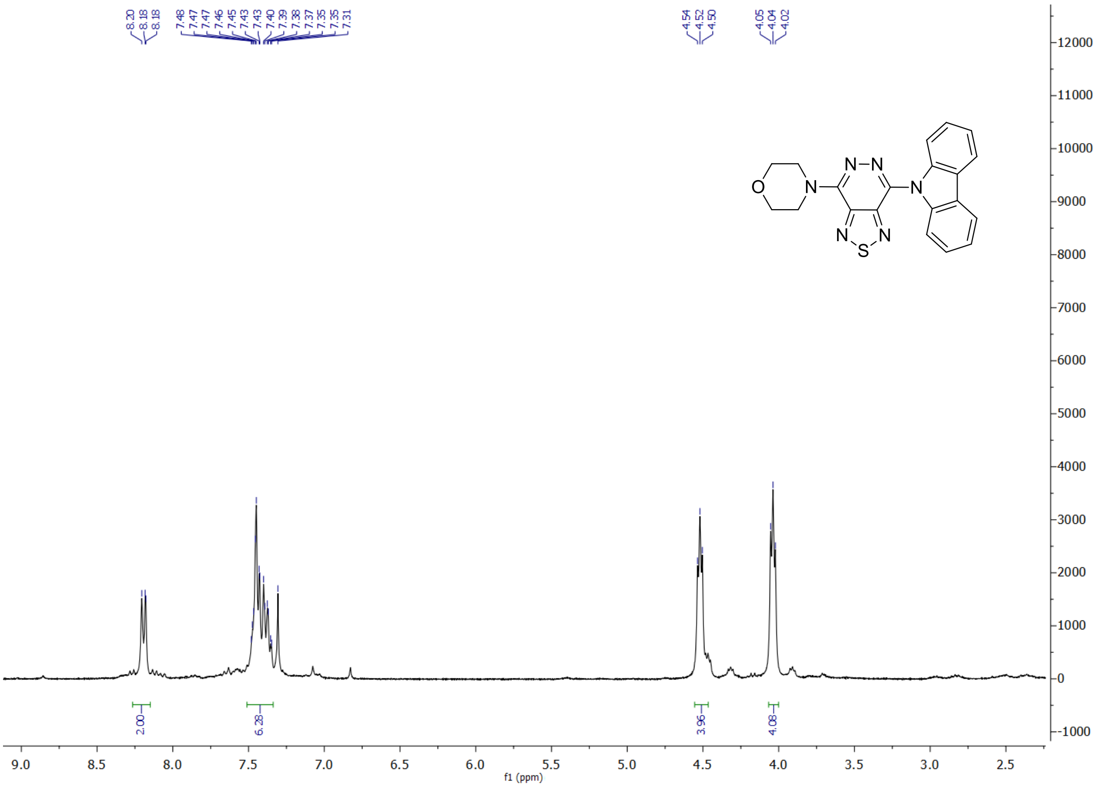

^{13}C-NMR(75 MHz)

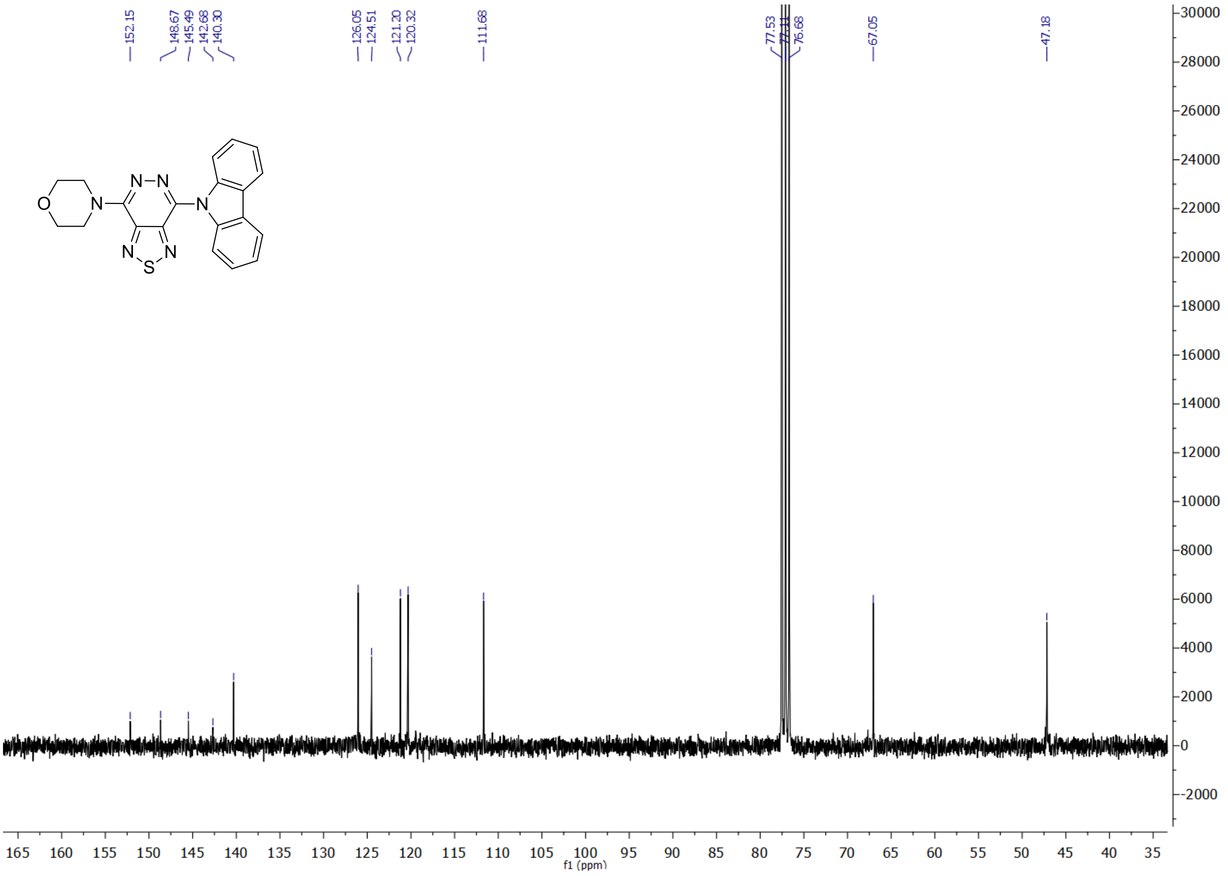

4,7-di(9H-Carbazol-9-yl)-[1,2,5]thiadiazolo[3,4-d]pyridazine (16)

^{1}H-NMR(300 MHz)

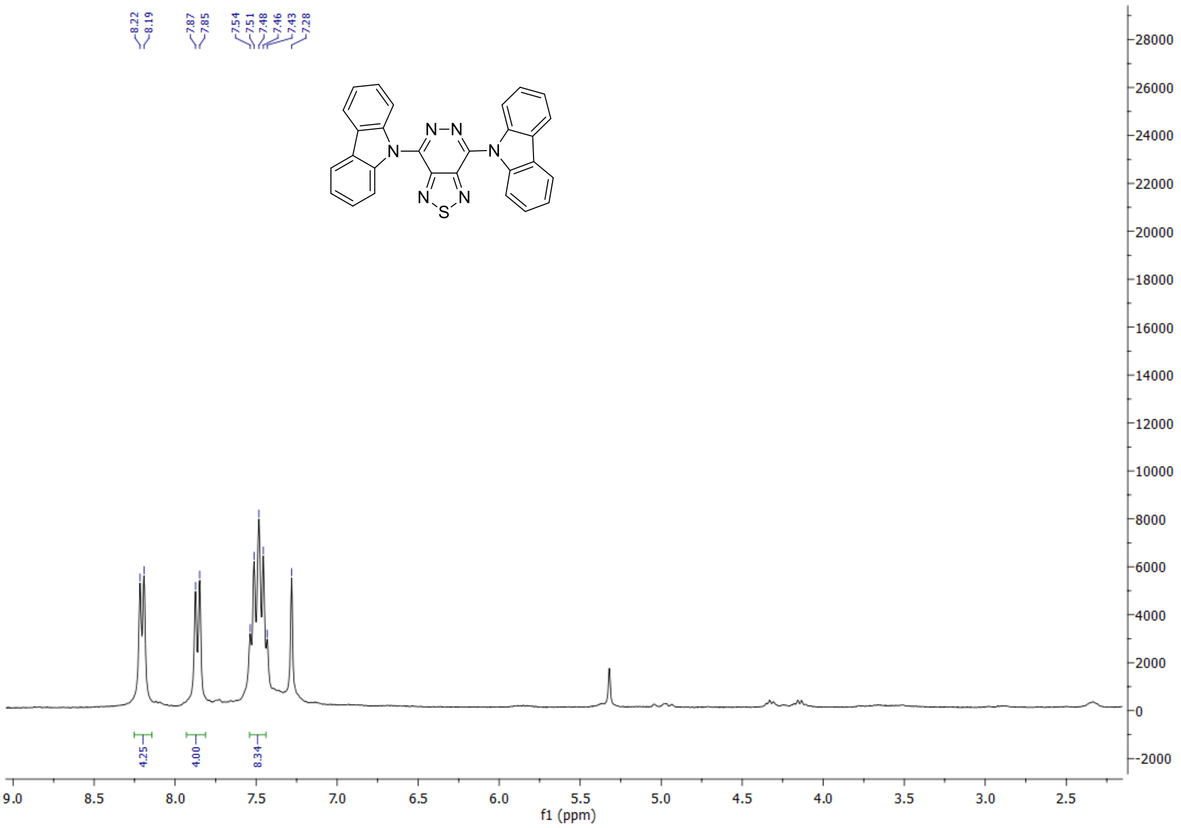

^{13}C-NMR(75 MHz)

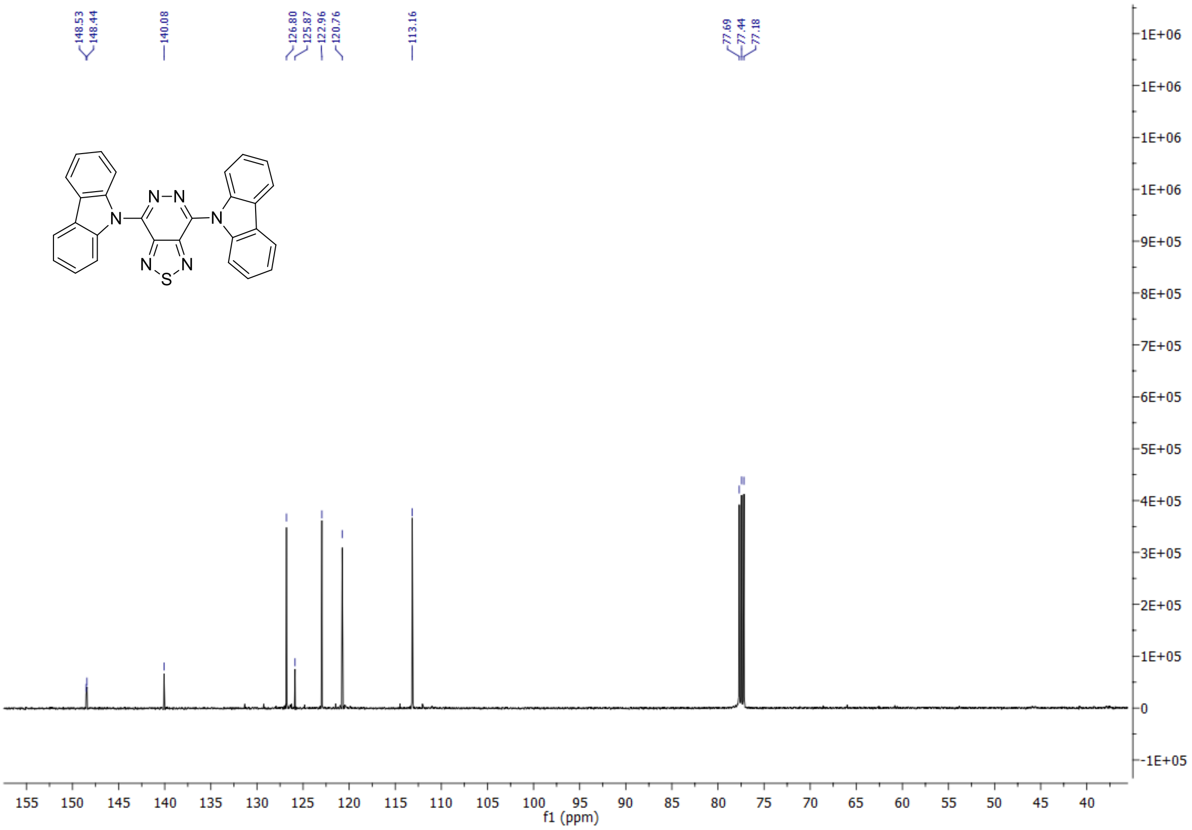

2. X-ray crystallography

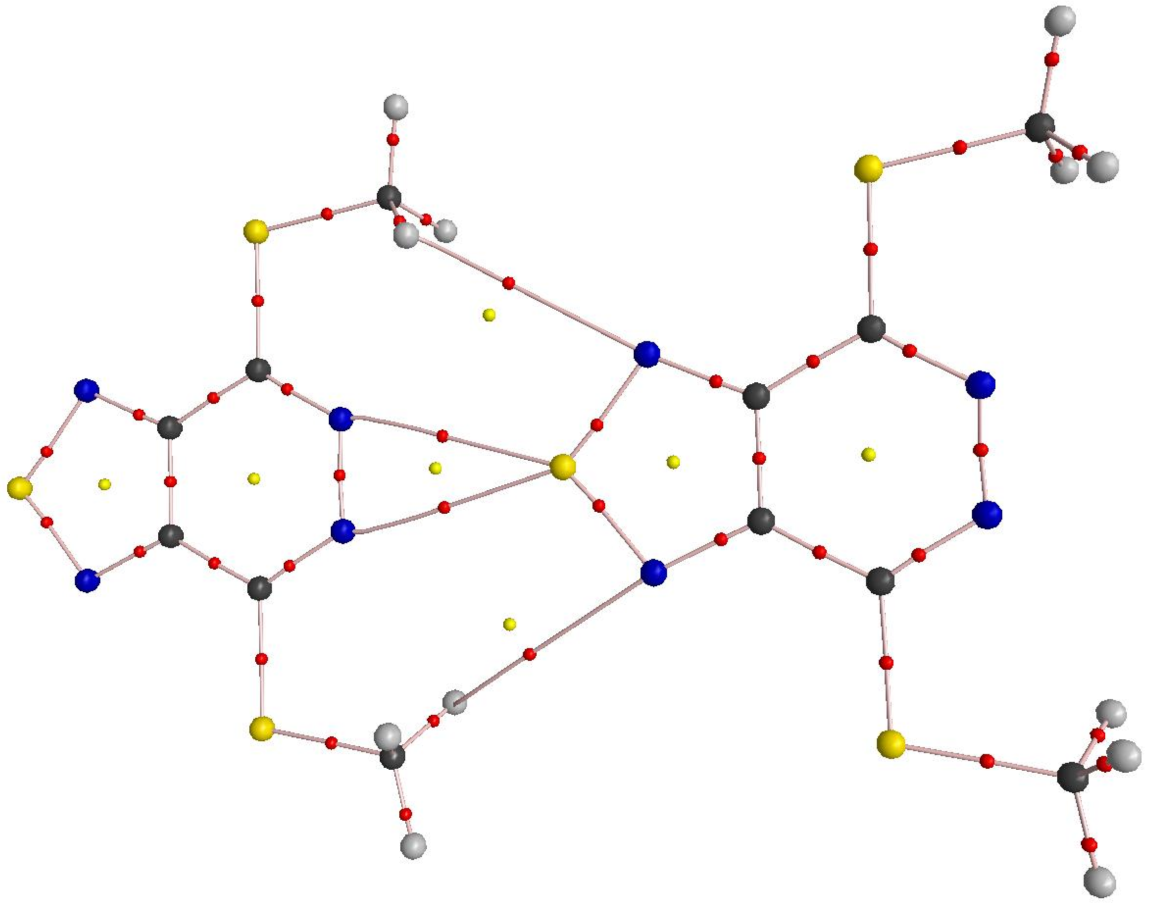

Figure S1. The molecular graph in the S…η^{2}-(N=N) bound dimer from the crystal **10b**. CP (3,-1) and (3,+1) are shown by small red and yellow balls, respectively.

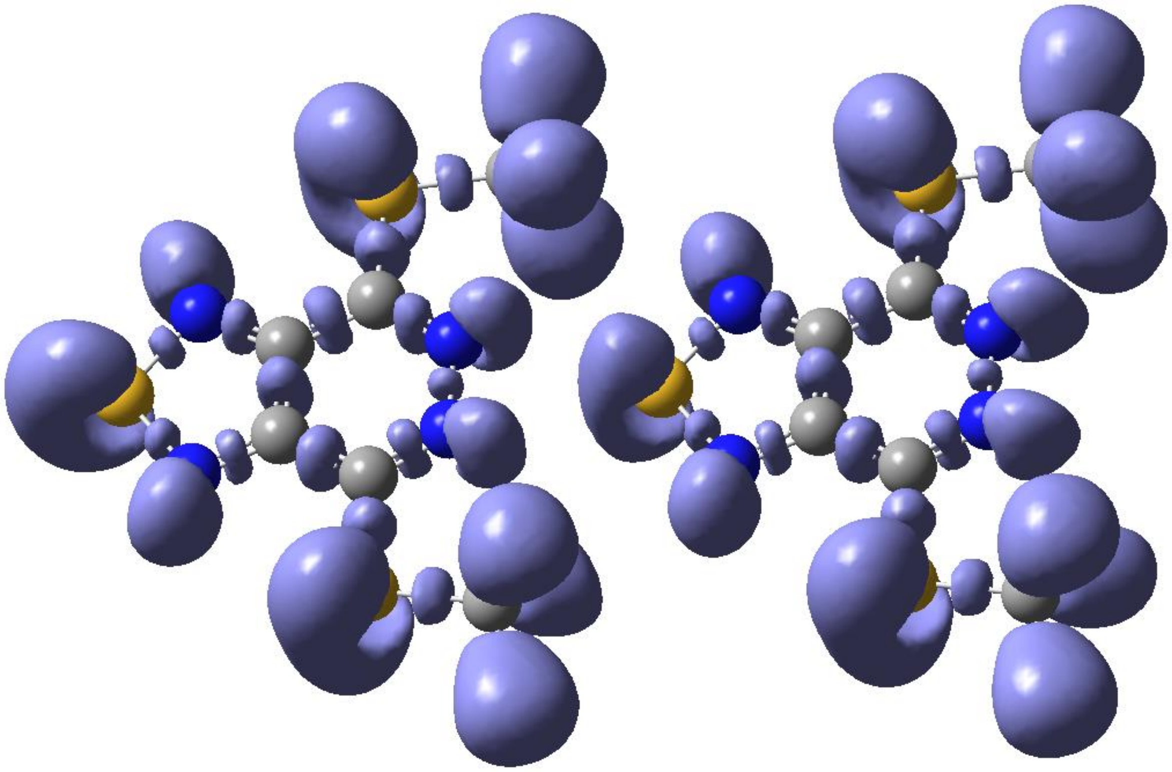

Figure S2. ELF distribution in the dimer from the crystal of **10b**. Isosurface for ELF =0.85 is shown.

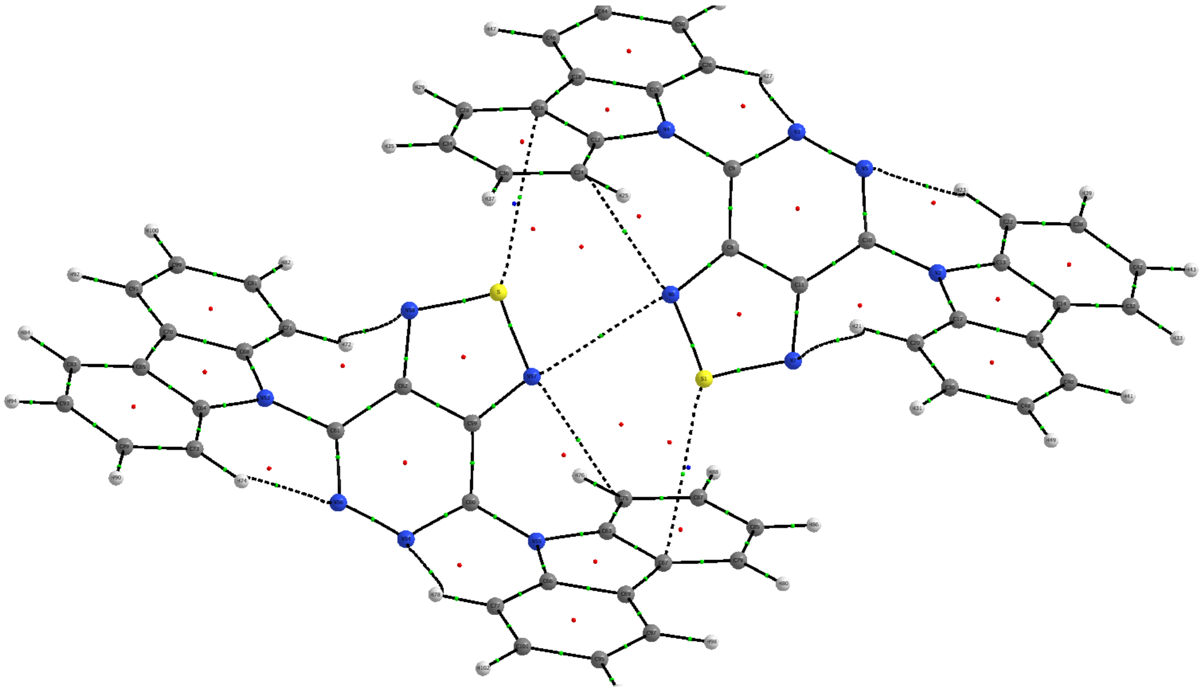

Figure S3. Molecular graph according to the topological analysis of electron density in the dimer from the crystal **16**. The bonding path are shown by the dotted lines. The CP (3,+1) are shown by small red balls.

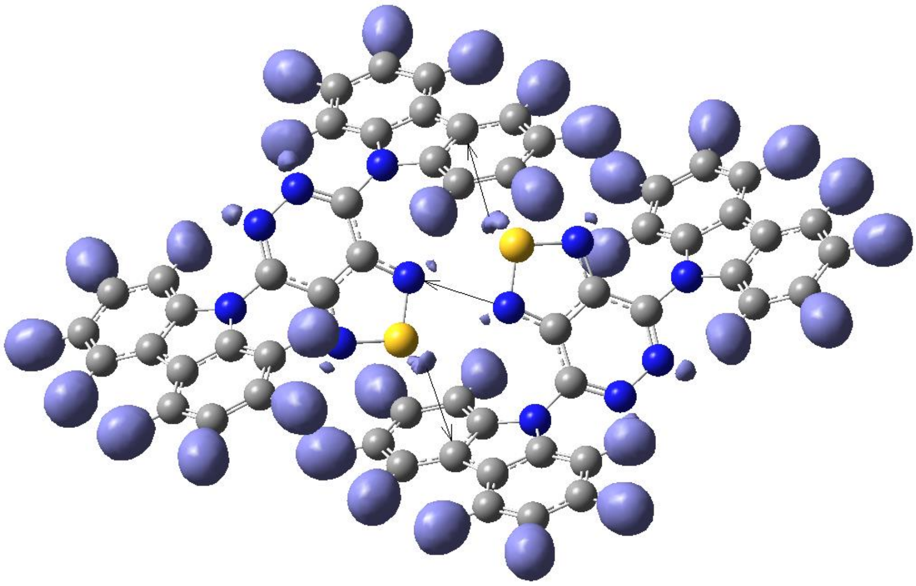

Figure S4. ELF distribution in the dimer from the crystal of **16**. Isosurface for ELF =0.95 is shown.
